# 3D DNA origami-enabled molecularly addressable optical nanocircuit

**DOI:** 10.1126/sciadv.aeb2001

**Published:** 2026-10-07

**Authors:** Jaewon Lee, Hayun Ahn, Kyung Hun Rho, Shelley F. J. Wickham, William M. Shih, Seungwoo Lee

**Affiliations:** ^1^ KU-KIST Graduate School of Converging Science and Technology, Korea University, Seoul 02841, Republic of Korea.; ^2^ School of Chemistry, School of Physics, The University of Sydney Nano Institute, The University of Sydney, Sydney, NSW 2006, Australia.; ^3^ Wyss Institute for Biologically Inspired Engineering, Harvard University, Boston, Massachusetts 02115, United States.; ^4^ Department of Cancer Biology, Dana-Farber Cancer Institute; Department of Biological Chemistry and Molecular Pharmacology, Harvard Medical School, Boston, Massachusetts 02215, United States.; ^5^ Department of Integrative Energy Engineering (College of Engineering) and Department of Biomicrosystem Technology, Korea University, Seoul 02841, Republic of Korea.; ^6^ Center for Opto-Electronic Materials and Devices, Post-Silicon Semiconductor Institute, Korea Institute of Science and Technology (KIST), Seoul 02792, Republic of Korea.

## Abstract

Clusters of nanoparticles enable electric and magnetic resonances and strong light–molecule interactions, including plasmonic resonance energy transfer (PRET). The optical nanocircuit concept provides a predictive RLC framework for these responses, but experimental realization has been limited by insufficient control over nanogaps and molecular placement. Here, we introduce a molecularly addressable optical nanocircuit based on a robust three-dimensional (3D) DNA origami. We demonstrate that gold nanoparticles and dye-loaded origami function as distinct circuit elements, with the latter acting as a resistor-coupled capacitor that directly participates in resonance. This capability enables deterministic assembly of nanocircuits with controlled symmetry and tunable nanogaps. The multilayer barrel scaffold yields a non–close-packed, symmetry-broken trimer with a magnetic resonance exhibiting a *Q*-factor of ∼19.2, exceeding values reported for comparable plasmonic clusters. Selective dye loading onto origami enables predictive light–molecule coupling, producing a 100-fold PRET enhancement in dimers over monomers. This approach provides a versatile route to designer optical resonances for nanophotonic applications.

## INTRODUCTION

Localized surface plasmon resonance (LSPR) of noble metal nanoparticles (NPs) has transformed our ability to manipulate light at the deep-subwavelength nanoscale by confining electromagnetic fields below the diffraction limit. This capability enables strong light-matter interactions, driving advances in molecular sensing, nonlinear optics, and quantum photonics (*1*–*6*). When plasmonic NPs are assembled into clusters, they provide additional control over electric and magnetic resonances (*7*–*10*). By tuning cluster configuration and interparticle spacing, resonant modes can be precisely tuned, thereby facilitating applications such as plasmonic resonance energy transfer (PRET) (*1*, *11*–*13*). A powerful theoretical framework guiding the design of these plasmonic clusters is the optical nanocircuit model, which establishes an analogy between nanoscale optical components and classical RLC circuit elements (*14*, *15*). RLC electrical circuits consist of inductors (*L*), capacitors (*C*), and resistors (*R*), and play a critical role in filtering and selectively tuning signals at radio frequencies. In the optical model, plasmonic NPs, nanogaps, and material losses (Ohmic losses) can be considered as optical analogs of idealized (i.e., lumped) circuit elements including *L*, *C*, and *R*, respectively. This approach enables the modular design of plasmonic clusters with precisely tailored electric and magnetic resonances.

The first experimental validation of an optical nanocircuit was demonstrated using dielectric nanorod arrays fabricated by electron-beam lithography (EBL), which functioned as two-dimensional (2D) optical circuit elements at mid-infrared wavelengths (*16*). Subsequent experimental approaches have included metallic and dielectric NP clusters assembled via atomic force microscope (AFM) manipulation, demonstrating circuit-like optical behavior at visible wavelengths (*17*). However, these existing methods lack nano-level accuracy in gap size and cluster geometry (e.g., ∼75 nm feature size for EBL and 3–8 nm gap size variation for AFM manipulation) and are not scalable. More critically, none of the reported optical nanocircuits to date have demonstrated the molecular addressability required for deterministic coupling between light and specific molecules, or practical applications such as PRET.

In this work, we introduce a DNA origami-enabled optical nanocircuit that is molecularly addressable and scalable. DNA origami is a technique in which a single-stranded DNA ‘scaffold’ strand is designed to self-assemble into custom 2D and 3D nanoscale shapes by binding of many short DNA ‘staple’ strands (*18*–*20*). The staple strands are uniquely addressable and can act as ‘handles’ on a programmable pegboard for high-precision spatial arrangement of plasmonic NPs and emitter molecules (*21*–*25*). In previous studies, dye molecules placed on such DNA origami-plasmonic NP hybrids acted purely as emitters or near-field reporters (*26*–*30*); they did not participate in the optical resonance itself, nor did they mediate resonance energy transfer as functional elements within an integrated optical circuit. Here, we demonstrate that DNA origami can also act as an idealized *R*-coupled *C* optical element. We show that the resistance of this *R* element can be precisely tuned by adjusting the loading of dye molecules on the DNA origami. Taking advantage of the molecular addressability of DNA origami, this *R* element can be integrated with plasmonic NP-based *L* and *C* elements in a highly programmable way. To enable such integration, we designed and used a barrel-shaped 3D DNA origami pegboard. This 3D DNA origami provides increased intrinsic mechanical robustness and a $C_{4}$-symmetric, molecularly addressable surface, allowing it to accommodate larger and ultrasmooth plasmonic NPs (up to 100 nm) necessary for strong plasmonic resonance. This platform enabled geometry-resolved assembly of addressable nanocircuit architectures and one-to-one structural–optical characterization at the individual-device level. As a result, these optical nanocircuits were able to support engineered magnetic resonances with a *Q*-factor of ∼19.2, a value higher than the 4–10 range reported for comparable plasmonic clusters, matching well with the circuit-model-based theoretical predictions. Importantly, the DNA origami-enabled coupling of dye molecules into this optical nanocircuit allowed programmable enhancement of light–molecule interactions. For example, the dimeric optical nanocircuits showed a 100-fold stronger PRET response than the monomeric counterpart.

## RESULTS

### Concept of molecularly addressable optical nanocircuit

A barrel-shaped DNA origami structure (henceforth referred to as barrel origami) was used to implement the optical nanocircuit designs (Fig. 1A). The dye-loaded barrel origami is designed to function as a lossy *C*, where the light-absorbing properties of the dye molecules result in non-radiative loss analogous to the resistive element in an electrical circuit (i.e., *R_nrad_*, where ‘*nrad*’ suffix refers to non-radiative loss within the coupled *C*). YOYO-3 was used as the dye molecule due to its ability to intercalate along DNA duplexes of the barrel through simple mixing and incubation. Importantly, the barrel origami possesses a hollow core; YOYO-3 dye molecules can be incorporated on both inner and outer surfaces, expanding the accessible dynamic range of *R*.

**Fig. 1.**
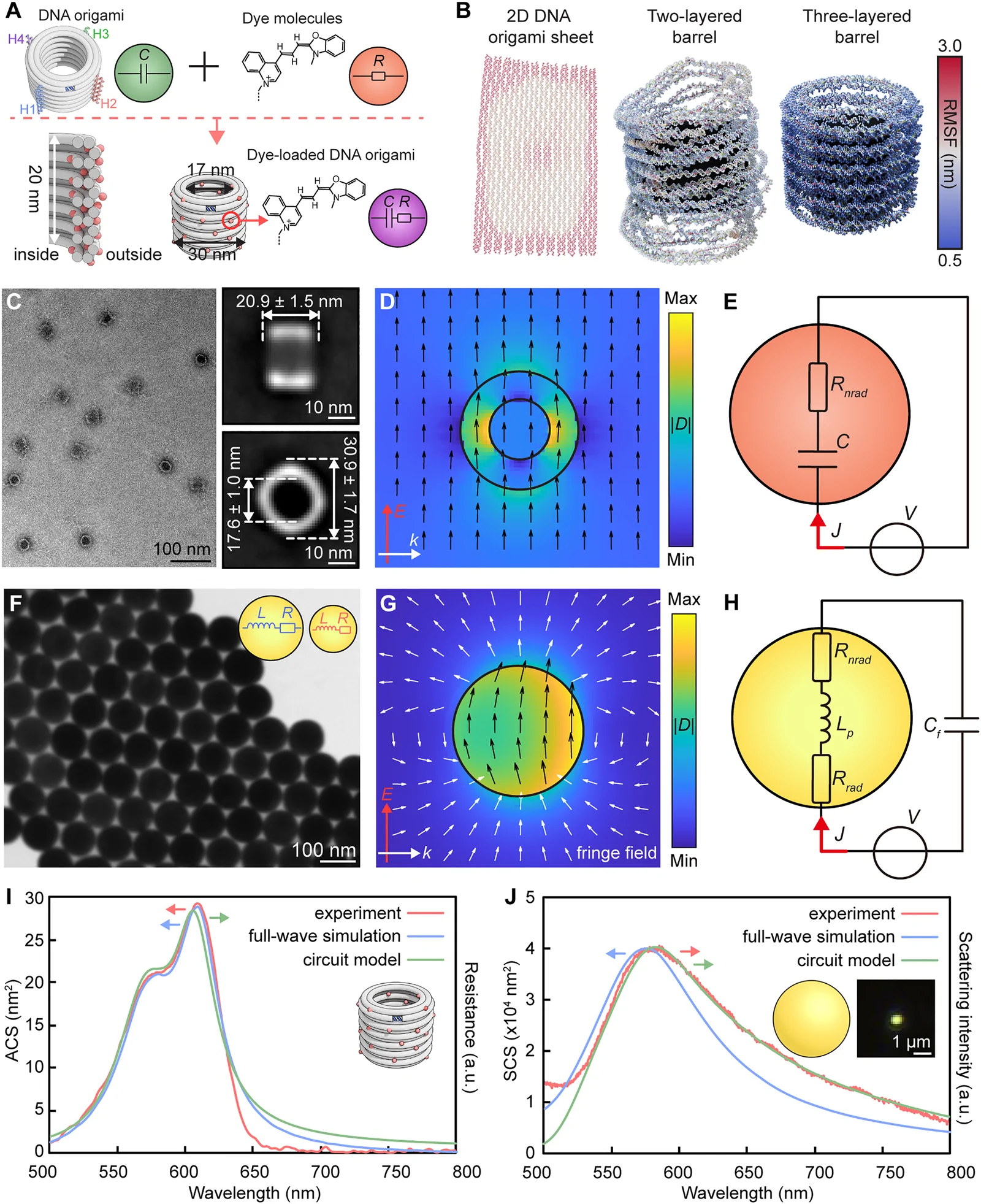
Basic elements of the molecularly addressable optical nanocircuit. (**A**) Barrel-shaped 3D DNA origami as a capacitive element (*C*); YOYO-3 loading adds nonradiative loss to produce an *R*-coupled *C* element. (**B**) oxDNA mean structures and RMSF maps for a 2D sheet, a two-layer barrel, and a three-layer barrel. (**C**) TEM overview and Scipion/Xmipp class averages generated from 100 side views and 100 top views of Au-NS-free three-layer barrels. Individual-particle measurements yielded a height of 20.9 ± 1.5 nm, an outer diameter of 30.9 ± 1.7 nm, and an inner diameter of 17.6 ± 1.0 nm (mean ± SD; *N* = 100 per orientation); the averages show the mean projected shape only. (**D** and **E**) Simulated electric-displacement field and equivalent *R*-coupled *C* circuit for the dye-loaded barrel. (**F**) TEM image of 100 nm Au NSs; inset, inductor-resistor representation. (**G** and **H**) Simulated dipolar fringe field and equivalent single-particle circuit with radiative and nonradiative losses. (**I**) Absorption cross section (ACS) spectra and resistive loss of dye-loaded barrels from experiment, full-wave simulation, and circuit model. (**J**) Scattering cross section (SCS) spectra of a single Au NS from the same three approaches; inset, DF image.

The barrel origami is composed of DNA duplex rings coaxially stacked to form outer, middle and inner layers, yielding a wall thickness of ∼7 nm. These layers are interconnected through a honeycomb lattice (also see fig. S1, Supplementary Materials). In this configuration, the concave side of the single-layer corrugated sheet faces outward, and each double helix is arranged horizontally with a length of 24 turns. The strand-nick sites located along each concave DNA duplex act as outer pixels for uniquely programmed single-stranded DNA (ssDNA) handles. Thus, these pixels are regularly and continuously spaced by ∼10 nm laterally and ∼ 5 nm vertically, while maintaining a central $C_{n}$ rotational symmetry (*n*-fold rotational axis). In this work, we adopt $C_{4}$ symmetry as a representative example. This modular, symmetric, and continuously addressable pixel array can be programmed to attach plasmonic NPs of either identical or heterogeneous sizes, enabling the assembly of symmetric or asymmetric 2D plasmonic clusters. Furthermore, individual pixels can be intentionally left unoccupied to introduce broken-symmetry into the plasmonic clusters.

Previous studies have used multilayer DNA molds with cuboid cavities, whose sidewalls consist of two or three layers of parallel DNA helices arranged on tetragonal or honeycomb lattices, to assemble plasmonic NPs (*31*, *32*). However, such molds inherently lack a central $C_{n}$ rotational symmetry and cannot support continuous pixelation around a symmetric wall. This limitation arises because their sidewalls consist of two or three layers of parallel DNA helices arranged on tetragonal or honeycomb lattices: each facet is either corrugated or relatively flat rather than forming a smoothly discretized cylindrical surface.

Meanwhile, the barrel origami offers substantial design versatility: its top and bottom lids can be functionalized with unique ssDNA handles, allowing programmable multimerization or binding of DNA-functionalized plasmonic NPs along the *x*-, *y*-, and *z*-directions. This modular connectivity enables not only 2D multi-barrel assemblies that reinforce structural precision and expand planar circuit motifs, but also true 3D optical nanocircuits formed through vertical stacking of barrels. Such 2D and 3D configurations greatly broaden the accessible resonant behaviors and further highlight the scalability of the barrel-origami–based optical nanocircuit platform.

Gold nanospheres (Au NSs) were chosen as representative plasmonic NPs due to their ultrasmooth surfaces, monodispersity, and high chemical stability, which suppress oxidation-induced optical losses (*33*–*36*). To accommodate more than three relatively large Au NSs (up to 100 nm) around the $C_{4}$-symmetric outer wall (H1–H4), a diameter ratio of ∼3.3 between the Au NSs and the barrel is required. Accordingly, the barrel origami was designed with an outer diameter of 30 nm (with an inner diameter of 17 nm and a height of 20 nm) (Fig. 1A).

Because even nanometer-scale structural deviations can strongly perturb the resonant modes of optical circuits (*37*, *38*)—for example, a few nanometers of nanogap variation can determine whether a Fano resonance is activated (*39*, *40*)—the barrel origami must exhibit low intrinsic fluctuation. To quantify its structural stability, we performed coarse-grained molecular dynamics simulations (oxDNA; fig. S2, Supplementary Materials) and analyzed the root-mean-square fluctuation (RMSF) of individual DNA bases within origami barrels (Fig. 1B). The simulation results, visualized as a colormap on the computed mean structure, reveal that most DNA bases exhibit sub-nanometer RMSF values. Notably, RMSF values are approximately 0.5 nm at the center of the sidewall where Au NSs are programmed to attach, demonstrating low intrinsic sidewall fluctuation for the three-layer barrel under the simulated solution condition. This analysis characterizes the unloaded scaffold; loaded, dried-grid geometry is evaluated separately from the measured Au NS positions and interparticle gaps.

These results were compared with those obtained for a two-layer barrel origami of similar outer diameter but a thinner ∼5 nm wall, as well as for conventional 2D DNA origami sheets commonly used for plasmonic NP assembly (*18*, *41*). As previously reported, the three-layer barrel is a 3D analog of a 2D DNA origami rectangle/pegboard (*21*). The 2D-sheet–to–two-layer-barrel–to–three-layer-barrel series is therefore a controlled within-design comparison of how closing a sheet into a barrel and subsequently reinforcing its wall by multilayering affects rigidity; it is not intended as a universal quantitative benchmark against every published 3D DNA origami. Both the two-layer barrel and the 2D sheet exhibit substantially larger RMSF values, often exceeding 1 nm (Fig. 1B). Representative 3D cages, molds, and packed bundles can also provide substantial rigidity. A matched oxDNA comparison among unrelated architectures would require harmonized dimensions, lattice density, crossover topology, and simulation protocols and is beyond the scope of this study. Accordingly, table S1, Supplementary Materials, reports quantitative RMSF only for the matched 2D-sheet/barrel design series and uses a qualitative, design-level comparison across unrelated architectures. Within that scope, the present barrel integrates $C_{4}$-symmetric z-parallel exterior handle rows, equivalent top/bottom modularity, a hollow PRET-active molecular reservoir, base-level RMSF ≤0.5 nm at the attachment wall, and deterministic assembly of multiple 100 nm Au NSs.

Figure 1C shows TEM images of the assembled Au-NS-free barrel origami, with close agreement to the designed dimensions (Fig. 1A and fig. S1, Supplementary Materials). The right panel presents class averages generated independently from 100 high-magnification top-view images and 100 high-magnification side-view images using the Scipion (Xmipp) software framework, consistent with established DNA origami structural characterization approaches (*42**–**44*). The 2D classification and averaging workflow is described in Supplementary Materials, section S25. To avoid extracting dimensions from the blurred edges of an averaged image, the outer and inner diameters were measured from the 100 individual top-view barrels, whereas the height was measured from the 100 individual side-view barrels before classification and averaging. The resulting projected dimensions were 30.9 ± 1.7 nm for the outer diameter, 17.6 ± 1.0 nm for the inner diameter, and 20.9 ± 1.5 nm for the height (mean ± SD; *N* = 100 per orientation; Supplementary Materials, section S30). The class averages are used only to visualize the mean projected shape; the individual-particle images and measurements provide the evidence for dimensional variability. Together, these data support the intrinsic structural uniformity of the unloaded barrel. They do not, by themselves, establish the barrel contour after Au NS loading and grid drying. Additional low-magnification TEM images are provided in fig. S3, Supplementary Materials.

Simulated electric displacement fields show slight convergence of the incident field into the YOYO-3-loaded barrel DNA origami upon dye excitation, resulting in electric-dipole (ED) oscillation and emission (Fig. 1D). The electric field distribution and power loss density are shown in fig. S4, Supplementary Materials. The corresponding circuit elements can be represented by a Thevenin equivalent nanocircuit diagram, as shown in Fig. 1E. Here, the polarization of the incident light is assumed to be vertical, so that the *R* and *C* elements are connected in series under the vertically applied voltage. Each YOYO-3-loaded barrel origami can be viewed as an “*R_nrad_*–*C*” component, where the specific values for these elements are determined from the effective permittivity of the YOYO-3-loaded origami (see details in Supplementary Materials). The experimentally estimated number of YOYO-3 dyes intercalated per barrel was ∼423 (see details in Supplementary Materials).

Next, we chemically synthesized ultrasmooth, uniform, and large (100 nm) Au NSs (see TEM images in Fig. 1F) using iterative growth and etching methods (see details in Supplementary Materials) (*33*–*35*). Low-magnification optical (dark-field, DF) and electron microscopy analyses, in conjunction with statistical analyses, were used to characterize the uniformity of Au NSs used in this study (see figs. S5 and S6, Supplementary Materials). We note that for such large Au NSs, the resonantly induced scattering, quantified by scattering cross section (SCS) in ED-based bright mode, can outperform absorptive loss (absorption cross section, ACS) (fig. S7, Supplementary Materials).

The numerically simulated fringe electric-displacement field induced by the ED mode is shown in Fig. 1G. Under illumination with vertically polarized light, the divergence and convergence of the induced fields (white arrows) at the top and bottom of the Au NS are referred to as the fringe field. Thus, in the corresponding nanocircuit model, *L_P_* coupled with *R* can be oriented along the light polarization direction (Fig. 1H). Also, note that *R* can be divided into two contributions: (i) *R_nrad_*, stemming from Ohmic loss under ED oscillation and (ii) radiative loss (*R_rad_*), originating from scattering. Thus, each Au NS can be viewed as “*R_nrad_*– *L_P_* – *R_rad_*.” As EDs, formed at the top and bottom of Au NSs, are coupled to the surrounding air medium, the “*R_nrad_*– *L_P_* – *R_rad_*” elements are connected in series with a fringe-field capacitor (*C_f_*). Note that this *C_f_* corresponds to the fringe in Fig. 1G.

We then experimentally validated the nanocircuit model predictions of spectral responses for the YOYO-3-loaded origami barrels (Fig. 1I) and Au NSs (Fig. 1J). Spectral dispersions of the resistance and scattering were analytically predicted using the nanocircuit model for YOYO-3-loaded barrels and Au NSs (green lines in Fig. 1, I and J; details in the Supplementary Materials). These analytical predictions were in good agreement with full-3D numerical calculations of ACS and SCS (see blue line in Fig. 1, I and J) using the finite element method (FEM, see details in Supplementary Materials). YOYO-3-loaded origami barrels (Fig. 1I) were experimentally characterized using UV-Vis absorption spectroscopy, showing absorption peaks at 613 and 575 nm wavelengths (red line, Fig. 1I). The experimental results were in good agreement with both analytical and numerical theoretical predictions.

For an Au NS, resonant energy exchange with the environment includes radiative fields and the associated radiative resistance (*R_rad_*). *R_rad_* is connected in series with *L_P_*– *R_nrad_*– *C_f_*, as shown in Fig. 1H. The analytical nanocircuit model predicts an ED resonance and a corresponding SCS peak near 570 nm wavelength. This was in good agreement with the numerically predicted FEM results (see green and blue lines of Fig. 1J). We used DF microspectroscopy to experimentally measure the ED-resonant scattering for individual Au NSs (red line of Fig. 1J and corresponding scattering image in inset of Fig. 1J, see Supplementary Materials). The measured scattering spectrum matched well with both the nanocircuit and FEM predictions. The nanocircuit parameters used in Fig. 1 are summarized in table S2. Overall, the two core components of our nanocircuit, the dye-loaded origami barrels and Au NSs, were confirmed to act as first-order optical nanocircuits.

### Programmable self-assembly of higher-order optical nanocircuits

Following characterization of the individual components, we next increased the complexity of the optical nanocircuit using the molecular addressability of the outer walls of DNA origami barrel. As noted above, the outer origami barrel can be symmetrically divided into four quadrants (inset of Fig. 2A), each of which can be programmed with a unique ssDNA handle sequence (H1–H4). The molecular addressability of each ssDNA handle was confirmed by super-resolution optical imaging (DNA-PAINT), as shown in Fig. 2A (fig. S8, details in Supplementary Materials). We assembled symmetric and asymmetric dimeric nanocircuits (second order) and experimentally quantified their resonant optical properties, as summarized in Fig. 2. In particular, we assembled two 100 nm Au NSs into a symmetric dimer configuration (Fig. 2B), while a 40 nm Au NS and a 100 nm Au NS were assembled at adjacent barrel quadrants (Fig. 2C).

**Fig. 2.**
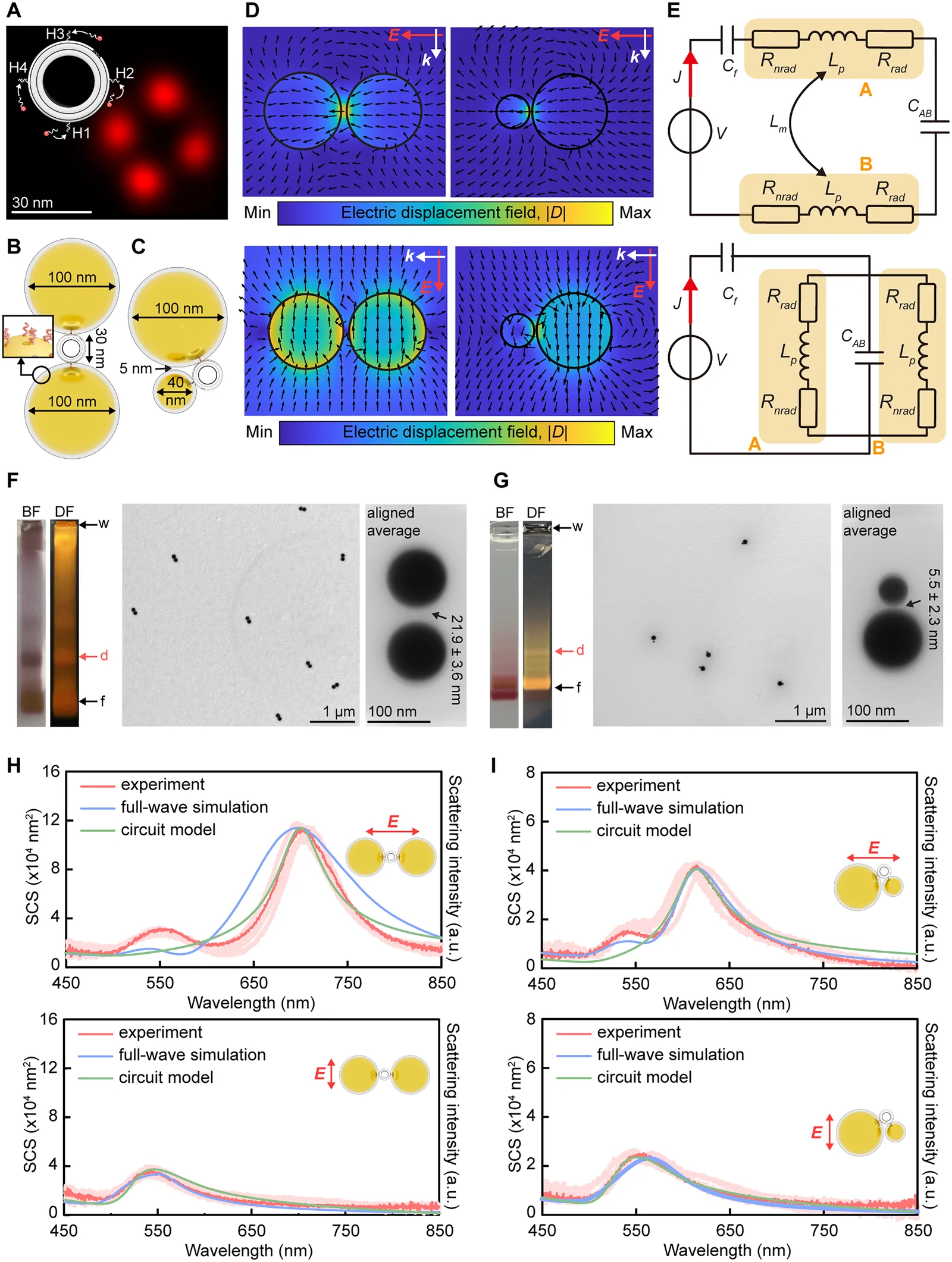
Dimeric optical nanocircuits assembled with barrel DNA origami. (**A**) Fourfold-symmetric exterior handle sites (H1-H4) and a DNA-PAINT image confirming molecular addressability. (**B** and **C**) Nominal designs of a symmetric 100/100 nm dimer with a ∼30 nm barrel-defined separation and a heterogeneous 100/40 nm dimer with a ∼5 nm gap. (**D**) Simulated electric-displacement fields for gap-parallel and gap-perpendicular excitation. (**E**) Corresponding lumped-element circuits. (**F** and **G**) Bright-field and dark-field gel images, representative low-magnification TEM images, and aligned averages (*N* = 100 per motif) for purified symmetric and heterogeneous dimers. (**H** and **I**) Polarization-resolved DF spectra compared with full-wave and circuit calculations. Reported gaps are edge-to-edge measurements from 100 stoichiometrically correct structures; paired FEM curves use the corresponding device TEM geometry. Device-specific inputs and gap-reading sensitivity envelopes are provided in Supplementary Materials, section S27.

Theoretical spatial distributions of the electric displacement field at ED modes under horizontal (top panel of Fig. 2D) and vertical (bottom panel of Fig. 2D) illuminations were then numerically simulated. Thus, the equivalent nanocircuit model (top panel of Fig. 2E) has to include both *C_f_*, responsible for the fringe of ED, and also *C_AB_*, responsible for capacitive light confinement at center-positioned dielectric barrel origami, denoted as hot spots. As the ED is formed along the direction of light polarization, these *C_f_* and *C_AB_* need to be arranged in a series circuit. Also, *C_AB_* is located between the two Au NSs (inductors) to reflect the capacitive coupling. As mentioned above, each Au NS, highlighted by “A” and “B” (yellow box in top panel of Fig. 2E), acts as “*R_nrad_*– *L_P_* – *R_rad_*.” In contrast, vertically polarized illumination enables independent ED resonances in each Au NS without coupling; accordingly, “*R_nrad_*– *L_P_* – *R_rad_*” element of Au NS and *C_AB_* can be arranged in a parallel circuit (yellow box in bottom panel of Fig. 2E).

We then assembled symmetric and asymmetric dimers with the barrel origami positioned at the center, as shown in Fig. 2, B and C. Symmetric dimers consisting of two 100 nm Au NSs and a nominal 30 nm barrel-defined separation were isolated by gel electrophoresis and characterized by low- and high-magnification TEM after purification. For visualization of the mean projected geometry, we generated an aligned average from 100 high-magnification images of structures with the correct stoichiometry (Fig. 2F); all individual images are shown in fig. S9, Supplementary Materials. Here, individual crops were translationally and rotationally aligned to a common principal axis in Fiji (ImageJ) and averaged pixel by pixel; no unsupervised classification was performed. The alignment and processing workflow is detailed in Supplementary Materials, section S25. We therefore use the term ‘aligned average’ rather than ‘class average’ for Au NS assemblies. The reported gap, 21.9 ± 3.6 nm, was calculated from individual edge-to-edge measurements rather than from the averaged image and is reported as a projected dry-state TEM gap. For this symmetric 100 nm dimer, the contraction relative to the nominal separation can be understood through a support-limited top-rim contact model. With barrel height *H* = 20.9 nm, outer radius *R_b_* = 15.45 nm, and Device A (Supplementary Materials, section S27) particle radius *r* = 48.3 nm, the projected gap is bracketed in this idealized model by $g_{\text{upright}}=2R_{b}=30.9\;\text{nm}$ for equatorial sidewall contacts and by $g_{\text{min}}=2\left\{R_{b}+\sqrt{r^{2}-{\left(r-H\right)}^{2}}-r\right\}=13.9\;\text{nm}$ for sphere-to-top-rim contact on the support film. The device-specific gap of 17.4 ± 0.2 nm and the population mean of 21.9 ± 3.6 nm fall within this idealized support-limited geometric range; thus, the observed contraction is consistent with—and can be quantitatively accounted for by—the top-rim contact model without requiring barrel collapse as its primary explanation (Supplementary Materials, section S28).

Asymmetric dimers were likewise isolated and characterized (Fig. 2G). The nominal geometry places the 40 nm and 100 nm Au NSs at adjacent $C_{4}$ sites and gives a ∼5 nm design gap. Individual measurements from 100 stoichiometrically target-positive structures yielded a projected dry-state gap of 5.5 ± 2.3 nm; the corresponding aligned average is shown only as a compact visualization, and all individual images are provided in fig. S10, Supplementary Materials. The geometric derivation of the nominal gaps for this and the higher-order motifs is provided in Supplementary Materials, section S26.

The SCS of the assembled dimers was predicted using the circuit model (Fig. 2, H and I, green) and FEM simulations (Fig. 2, H and I, blue) and compared with polarization-resolved DF spectra of individually correlated dimers (Fig. 2, H and I, red). For each displayed optical device, its geometry and orientation were determined from the TEM image of that same device (figs. S11 to S13, Supplementary Materials). Thus, the nominal design gap, the population-level projected dry-state gap, and the device-specific FEM input gap are distinct quantities. The FEM curves paired with the experimental spectra use the third quantity—the dimensions of the individually correlated device—and not the gap label in the ensemble schematic (Supplementary Materials, section S27). In this correlative framework, isolated, stoichiometry-verified nanocircuits were identified on TEM grids prior to optical acquisition, enabling the direct evaluation of intrinsic optical reproducibility independently of bulk assembly yields. A comprehensive summary of all measured devices (*N* = 10 per motif), mode occurrence rates, and representative mode-deviant exceptions is provided in Supplementary Materials, section S29.

The capacitive coupling between the two 100 nm Au NSs makes the ED mode highly sensitive to the nanogap, with stronger coupling leading to a redshifted ED mode at 700 nm wavelength and a higher-order quadrupolar mode (EQ) at 550 nm wavelength. Consistent with the structural analysis, the 3.6 nm standard deviation (s.d.) in gap size resulted in a correspondingly narrow spread in the ED resonance peak under horizontal illumination. The *Q*-factor of ED scattering was found to be ∼5.9 (details in fig. S14, Supplementary Materials). In contrast, under vertical illumination, the ED mode is uncoupled and therefore minimally affected by nanogap variations. The corresponding *Q*-factor was found to be approximately 3.51 (details in fig. S14, Supplementary Materials). Similarly to the horizontal case, the vertical illumination nanocircuit model and FEM simulation both agreed well with the DF scattering measurement (Fig. 2H).

Note that the nanocircuit model only captures the ED resonance, not the higher-order EQ peak. This is a fundamental limitation, as the lumped circuit elements for each NP are derived from a dipolar quasistatic approximation. Therefore, the model cannot describe the EQ mode in this dimer, which involves non-dipolar charge oscillations within the individual NPs (see fig. S15A, Supplementary Materials). The full-wave FEM simulation correctly predicts the EQ peak, although its position appears at a slightly shorter wavelength than the measured value, likely due to minor geometric differences in the model compared to the assembled structures.

For the asymmetric system, the ED resonances occur at shorter wavelengths than the symmetric system (for horizontal polarization: from 700 nm to 633 nm wavelength), due to the smaller size of one of the Au NSs. The asymmetric dimer showed higher measured ED-scattering *Q*-factors than the symmetric dimer under both polarizations (9.75 versus 5.9 for gap-parallel excitation and 4.62 versus 3.51 for gap-perpendicular excitation; fig. S14, Supplementary Materials). This increase in *Q*-factor of ED scattering could be due to both decreased radiative loss from the smaller Au NS and the reduced gap between the Au NSs. Indeed, *R_rad_* of the asymmetric dimer was found to be smaller than for the symmetric dimer (see details on nanocircuit parameters, summarized in table S3, Supplementary Materials). Similar to the case of the symmetric dimer, however, the dipolar circuit model does not capture the higher-order EQ mode (see fig. S15B, Supplementary Materials).

Third- and fourth-order nanocircuits consisting of trimeric and tetrameric assemblies were then assembled. A combinatorial approach using 40 nm and 100 nm Au NSs allowed for assembly of trimers and tetramers with controlled symmetry and heterogeneity (Fig. 3, A to D). The four motifs were: (i) two 100 nm Au NSs and one 40 nm Au NS with mirror symmetry and heterogeneity (Fig. 3A); (ii) two 40 nm Au NSs and one 100 nm Au NS with mirror symmetry and heterogeneity (Fig. 3B); (iii) three 100 nm Au NSs with mirror symmetry without heterogeneity (Fig. 3C); (iv) two 100 nm Au NSs and two 40 nm Au NSs with mirror symmetry (Fig. 3D).

**Fig. 3.**
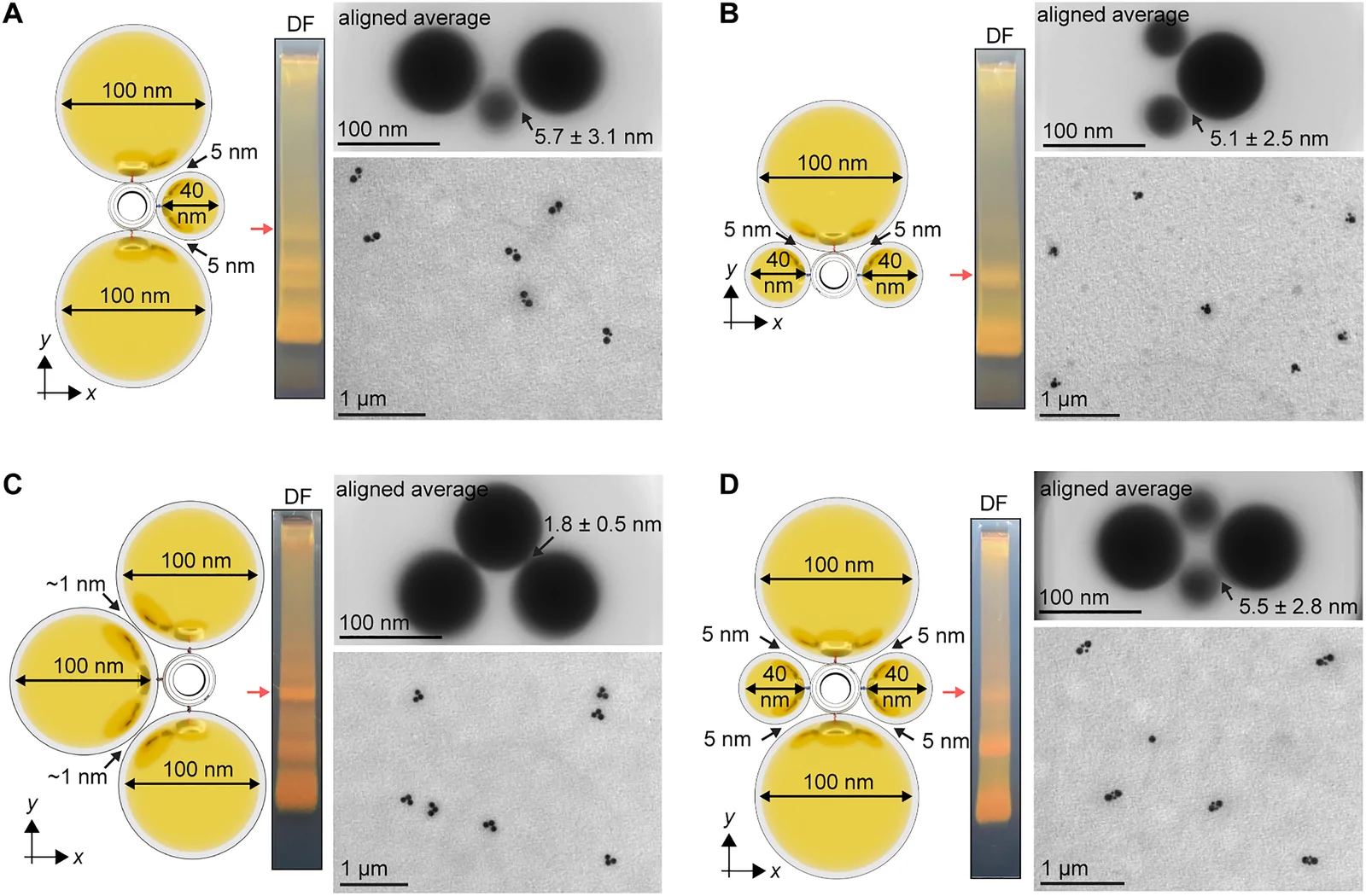
Assembly of higher-order Au NS optical nanocircuits using barrel origami. (**A** and **D**) Nominal design schematics of the nanocircuits (left), DF gel images highlighting the purified bands (red arrows), aligned-average image of high-magnification TEM images (top right in each panel), and representative low-magnification TEM images (bottom right) for each motif. (A) Heterogeneous trimer composed of two 100 nm and one 40 nm Au NSs, with the barrel programmed to create ∼5 nm nominal gaps on both sides of the 40 nm NS; individual analysis of 100 stoichiometrically target-positive structures yields a mean ± SD gap of 5.7 ± 3.1 nm. (**B**) Heterogeneous trimer consisting of one 100 nm and two 40 nm Au NSs with nominal ∼5 nm gaps; analysis of 100 stoichiometrically target-positive structures gives a mean ± SD gap of 5.1 ± 2.5 nm. (**C**) Symmetric trimer of three 100 nm Au NSs with nominal ∼1 nm gaps; individual analysis of 100 stoichiometrically target-positive structures gives a mean ± SD gap of 1.8 ± 0.5 nm. (D) Symmetric tetramer comprising two 100 nm and two 40 nm Au NSs with nominal ∼5 nm gaps; individual analysis of 100 stoichiometrically target-positive structures yields a mean ± SD gap of 5.5 ± 2.8 nm. Across all motifs, these *N* = 100 stoichiometrically target-positive datasets quantify projected dry-state geometry conditional on the intended stoichiometry; they are not unbiased total fabrication-yield measurements.

As with the dimers, the trimeric and tetrameric assemblies were characterized by low- and high-magnification TEM after gel purification. For Fig. 3, A to D, aligned averages were generated from 100 stoichiometrically target-positive high-magnification images per motif (figs. S16 to S19, Supplementary Materials) to visualize the mean projected geometry; no classification was performed. The reported gaps were calculated from individual structures and are 5.7 ± 3.1 nm (Fig. 3A), 5.1 ± 2.5 nm (Fig. 3B), 1.8 ± 0.5 nm (Fig. 3C), and 5.5 ± 2.8 nm (Fig. 3D). These values are projected dry-state TEM gaps. Detailed measurement protocols for these projected dry-state gaps, including calibrated pixel size, boundary criteria, and repeat-measurement precision, are reported in Materials and Methods and Supplementary Materials, section S27. Differences between these measured gaps and the corresponding nominal design values are construct dependent rather than uniformly contracting or expanding, so capillary drying is treated as one component of a broader grid-preparation and projection perturbation (Supplementary Materials, section S28).

Higher order nanocircuits enable access to two important optical modes that would otherwise be impossible to achieve. Firstly, clusters of more than two NPs can complete a ring of three capacitors, which can induce unnatural magnetic resonance via capacitively induced circulation of the electric field displacement (Fig. 4A). As representative examples, we include the MD mode analyses for cases (iii) and (iv) in Fig. 4A, showing the circulating electric displacement field. Regardless of the polarization of illumination, ring-like configurations of Au NS inductors (“*R_nrad_*– *L_P_* – *R_rad_*” elements, labeled A, B, and C for trimers; A, B, C, and D for tetramers) and capacitors (*C_ab_*, *C_ac_*, and *C_bc_*, for trimers; *C_ac_*, *C_ad_*, *C_bc_*, and *C_bd_*, for tetramers) occur in both trimeric and tetrameric nanocircuits (Fig. 4B).

**Fig. 4.**
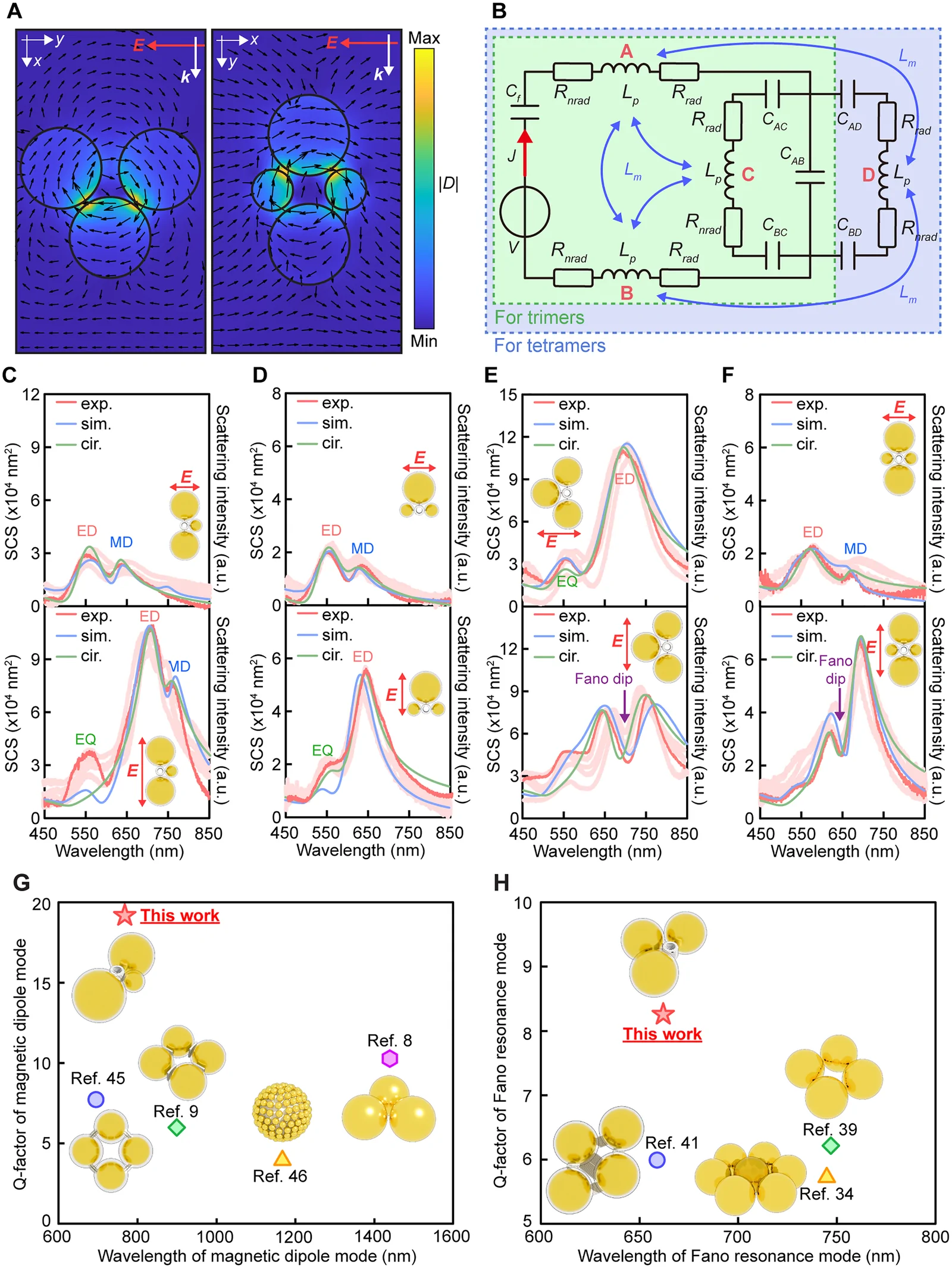
Optical responses of barrel-origami-templated trimeric and tetrameric nanocircuits. (**A**) Full-wave electric-displacement fields for representative ring-type trimers and tetramers under two orthogonal polarizations, showing circulating displacement currents associated with MD excitation. (**B**) Lumped-element circuits for the trimeric and tetrameric ring resonators. (**C** to **F**) Polarization-resolved DF spectra of the four motifs in Fig. 3 compared with full-wave and circuit calculations; ED, MD, EQ, and Fano features are indicated. (**G**) MD-mode *Q* factor versus resonance wavelength for the present trimer (red star) and reported plasmonic clusters (*8*, *9*, *51*, *64*). (**H**) Fano-resonance *Q* factor versus wavelength for the symmetry-broken trimer (red star) and reported Fano-type clusters (*34*, *39*, *41*). Device-specific particle diameters and gaps used for each paired FEM curve, together with gap-reading sensitivity envelopes based on technical measurement repeatability, are provided in Supplementary Materials, section S27.

For each design, five one-to-one TEM/DF examples are shown in figs. S20 to S23, Supplementary Materials. Experimental DF spectra from the ring resonators show well-resolved MD modes in the visible range and agree with the theoretical predictions (Fig. 4, C to F; fig. S24 and tables S4 and S5, Supplementary Materials). These paired data establish structure–spectrum correspondence for the displayed devices possessing the correct stoichiometry. Because the correlative workflow pre-screens isolated structures with correct stoichiometry, these measurements evaluate optical reliability rather than overall chemical fabrication yields. The complete paired TEM–DF datasets (*N* = 10 per motif), including every device counted in the mode summary, and their scope are detailed in Supplementary Materials, section S29. The geometric parameters used for these numerical calculations and their standardized positions (z-scores) within the corresponding *N* = 100 population distributions are reported in Supplementary Materials, section S27. In cases (i), (ii), and (iv), distinct scattering shoulders in addition to the ED-driven peaks (Fig. 4, C, D, and F) provide evidence that the MD resonance is excited independently rather than only through ED coupling. Depending on incident polarization, the ED and MD resonances appear at different wavelengths and amplitudes, consistent with the calculated current circulation, supporting reproducible optical fidelity across stoichiometrically correct nanocircuits.

A key factor underlying this unusually high *Q*-factor (Fig. 4G) is the non–close-packed, symmetry-broken configuration enabled by our barrel origami scaffold. This architecture supports resonators composed of the minimal number of Au NSs and achieves the smallest overall form factor. For example, the barrel origami allows the assembly of a trimer consisting of two large (100 nm) and one small (40 nm) Au NSs with a deterministic and precisely controlled symmetry break. Introducing a smaller perturbing Au NS suppresses retardation and reduces ohmic losses across the entire cluster, which is analogous to the role of an asymmetric gap in a split-ring resonator used in left-handed metamaterials (*45*). Notably, even this subtle perturbation within an otherwise symmetric 100 nm Au NS dimer is sufficient to induce a stable current circulation. As a result, a small but deliberately engineered asymmetry becomes the key to achieving the strongest MD resonance, even among the smallest Au NS clusters available. Using two small (40 nm) and one large (100 nm) Au NSs with the same symmetry-broken geometry [case (ii)] further reduces the form factor. However, the ED of each Au NS and the capacitive coupling required to drive current circulation are weaker in this configuration than in case (i) due to two small Au NSs, resulting in a lower MD-mode *Q*-factor. Similarly, the symmetric cluster composed of two large (100 nm) and two small (40 nm) Au NSs [case (iv)] exhibits a smaller *Q*-factor than case (i), owing to its larger overall size, which strengthens retardation, and its preserved symmetry.

In addition to the independently excitable ED and MD modes described above, ring-type plasmonic resonators can also support energy transfer from the “bright” ED mode to the “dark” MD mode, giving rise to a Fano resonance (*39*, *46*). For this to occur, the combined phase shift associated with coupling from the bright to the dark mode must approach π (*47*), and the spectral bands of the ED and MD modes must partially overlap. This typically requires a broadband ED response and a spectrally sharp MD mode (*39*). Consequently, previous studies have relied on clusters composed exclusively of large Au NSs (e.g., ∼200 nm) to generate a sufficiently broad ED resonance and to spectrally match it with the MD mode (*8*). However, in symmetric clusters, the ED and MD modes remain orthogonal and therefore contribute additively to the total SCS, as observed in cases (i), (ii), and (iv). To induce Fano interference, this orthogonality must be broken. Numerous studies have attempted to introduce asymmetry into large-Au-NS clusters to achieve this condition (*8*, *39*, *46*, *47*). Although cases (i), (ii), and (iv) incorporate particle-size heterogeneity, with (iv) exhibiting a weak Fano signature under gap-perpendicular excitation, the inclusion of a small 40 nm Au NS produces an ED mode that is not sufficiently broadband, preventing the formation of a well-defined Fano lineshape.

Traditionally, achieving simultaneous broadband ED excitation and π-phase-matched MD coupling was thought to require at least four or more large Au NSs arranged in a ring-like inclusion (tetramer or heptamer as summarized in fig. S25, Supplementary Materials) (*8*, *39*, *46*, *47*). In contrast, our work demonstrates that a high-quality Fano resonance was achieved even with only three large (100 nm) Au NSs, as realized in case (iii). In this trimer, the mirror symmetry is deterministically broken under horizontal polarization because the 1–2 and 2–3 gaps are approximately 1 nm, whereas the 1–3 separation is ∼20 nm. This engineered asymmetry yields strong ED–MD interference and results in a Fano resonance with a *Q*-factor above those in the cited comparison set (Fig. 4, E and H) (*34*, *39*, *41*). For the paired numerical simulation in Fig. 4E, the geometric inputs were measured from this individual case (iii) device (measured gaps of 3.2 and 3.1 nm, corresponding to +2.8 and +2.6 s.d. relative to the population mean of 1.8 ± 0.5 nm; table S8, Supplementary Materials), establishing a deterministic structure–spectrum correspondence for this specific characterized geometry alongside the independently reported population statistics. This Fano resonance was also well predicted by the circuit model with the appropriate parameters (summarized in table S4, Supplementary Materials). We attribute the improved *Q*-factors to the use of a relatively small number of Au NSs and the correspondingly reduced overall cluster size, which together minimize retardation effects and lower radiative losses, thereby increasing the *Q*-factor. The high rigidity and nanoscale assembly accuracy of the DNA barrel, combined with the ultrasmooth surfaces of the Au NSs, enabled the reliable construction of large-Au NS-based trimers with well-defined gaps. The Fano feature was observed in 8 of the 10 measured 3 × 100 nm trimer devices; five representative one-to-one TEM–DF examples are shown in fig. S22, Supplementary Materials.

### Multi-barrel and 3D optical nanocircuit

In addition to the single-barrel–based 2D optical nanocircuits, the inherent modularity and hierarchical expandability of the barrel-origami enable the reliable construction of multi-barrel optical nanocircuits. Because each barrel origami provides well-defined, symmetrically arranged attachment sites and exhibits robust mechanical rigidity, multiple barrels can be programmably linked to form larger and more complex nanocircuit motifs. This multi-barrel architecture extends the accessible geometric design space beyond that of a single barrel, allowing the assembly of higher-order, symmetry-engineered clusters as follows.

First, we co-assembled two 30 nm DNA barrel origami scaffolds with two 60 nm Au NSs to form a structurally reinforced dimeric nanocircuit (Fig. 5A; figs. S26 and S27, Supplementary Materials). We intentionally used 60 nm, rather than 100 nm, Au NSs as a pivoting-sensitive stress test because their smaller contact footprint makes projected rotational variability easier to visualize. The 60 nm stress-test system is therefore distinct from the 100 nm nanocircuits analyzed in Figs. 2 to 4. To quantify loaded-state cross-sectional geometry, we measured the projected aspect ratio across the 100-image gallery. The loaded barrel exhibited an aspect ratio of 1.16 ± 0.11 (mean ± s.d., N = 100), compared with 1.08 ± 0.07 for the Au-NS-free barrel origami (Supplementary Materials, section S28). This modest increase and broadening indicate limited load- and drying-associated deformation, while the mean projected shape remains close to circular. In the dual-barrel design, two independent constraints reduce the 60 nm dimer gap dispersion, pivoting angle, and lateral displacement (fig. S28 and table S7). The aligned averages in Fig. 5A are mean-geometry visualizations only; the quantitative comparison is based on individual structures. The improved geometric constraint is reflected in the DF spectra (Fig. 5B; figs. S29 and S30, Supplementary Materials), which show a more reproducible gap-mode ED peak and closer agreement with FEM and the circuit model.

**Fig. 5.**
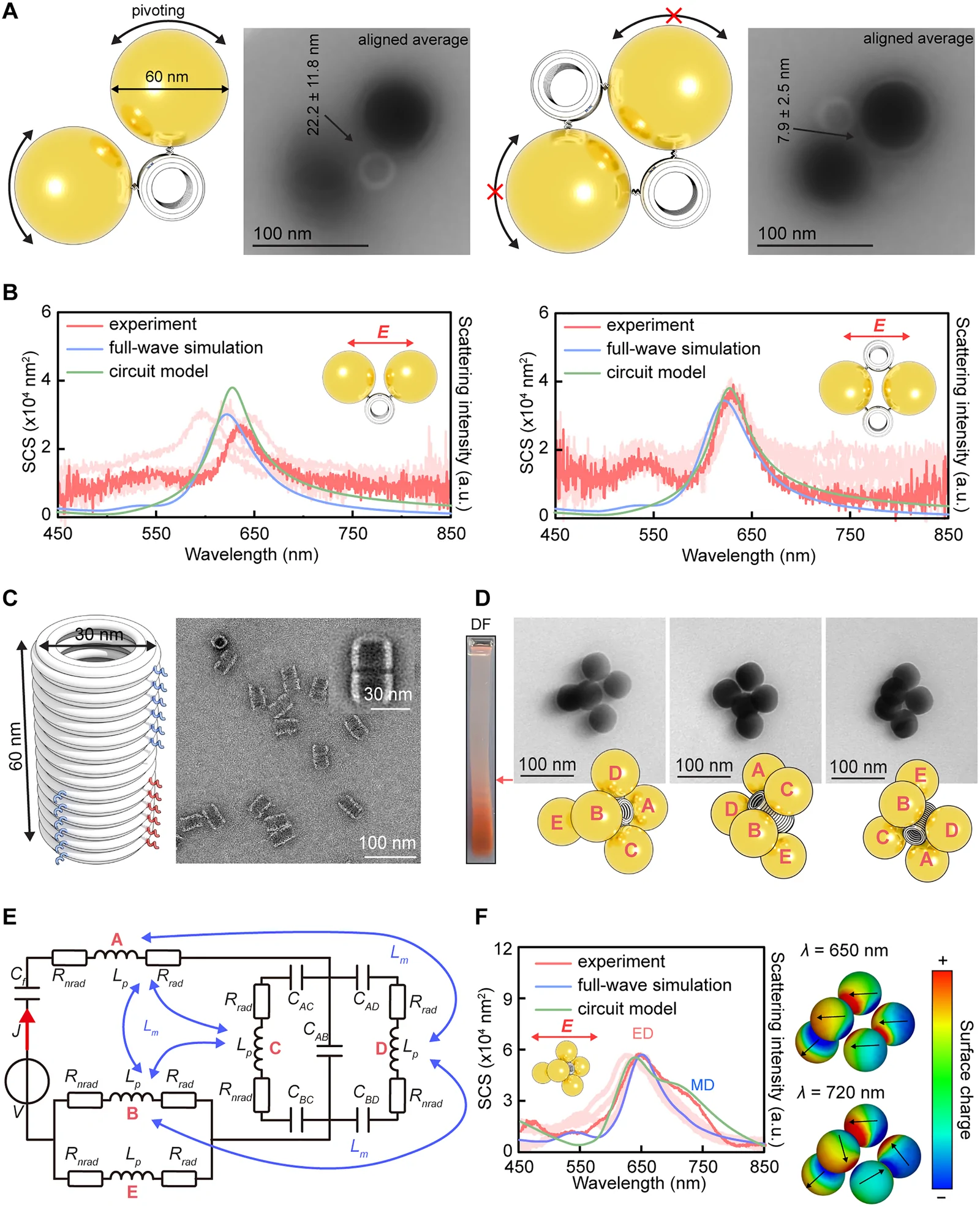
Multi-barrel-enabled stabilization and 3D extension of optical nanocircuits. **A**) Schematics and aligned averages (*N* = 100 per condition) of 60 nm Au NS dimers assembled with one or two barrels; dual-barrel attachment reduces projected pivoting and gap variability. (**B**) Gap-parallel DF spectra compared with full-wave and circuit calculations. (**C**) Schematic and TEM images of a 60 nm scaffold formed by vertical stacking of two 30 nm barrels. (**D**) DF gel and TEM images of five-particle clusters assembled around the stacked scaffold. (**E**) Lumped-element model of the 3D pentamer. (**F**) Experimental, full-wave, and circuit-model spectra showing ED and MD resonances; charge maps at 650 and 720 nm identify the modes. Aligned averages in (A) visualize mean geometry; individual distributions and the 100 nm reference are provided in fig. S28 and table S7. Device-specific inputs and gap-reading sensitivity envelopes for the dual-barrel dimer and pentamer are provided in Supplementary Materials, section S27; the single-barrel curve in (B) is a reference calculation without a same-device envelope.

Second, beyond improving 2D assemblies, the multi-barrel strategy enables the construction of 3D optical nanocircuits by vertically stacking two 30 nm barrels to form taller barrel origami scaffolds (Fig. 5C). The TEM images confirm that this vertical dimerization yields a vertically extended 60 nm-tall template capable of hosting Au NSs (e.g., 60 nm) in true 3D geometries rather than restricting them to the equatorial plane.

Third, these taller 3D scaffolds allow the assembly of higher-order 3D plasmonic motifs such as symmetry-broken pentamers (Fig. 5D): see more TEM images in fig. S31, Supplementary Materials. DF gel purification cleanly isolates the pentamer band, and representative TEM images show the intended five-particle arrangement around the vertically extended barrel scaffold. This architecture enables deliberate control of 3D symmetry, either preserved or selectively broken, providing access to a broader range of resonant modes than achievable with planar designs.

Fourth, the corresponding 3D Thevenin-equivalent nanocircuit (Fig. 5E) captures inductive, capacitive, and mutual-inductive coupling among all five nanoparticles in the pentamer. Each Au NS is represented by an *L*–*R* branch, and interparticle interactions are mapped through capacitances ($C_{\mathrm{AB}}$, $C_{\mathrm{AC}}$, …) and mutual inductances $L_{m}$. This model accurately predicts the experimentally observed scattering features.

Finally, the measured scattering spectra of the 3D pentamer (Fig. 5F, see also fig. S32, Supplementary Materials for correlative TEM-DF analysis) exhibit well-resolved ED and MD resonances. Full-wave simulations and circuit-model calculations show excellent agreement with experiment. Surface-charge maps (right) clearly identify the modal nature of the resonances at λ = 650 nm (ED mode) and λ = 720 nm (MD mode), confirming that the multi-barrel strategy supports complex 3D optical nanocircuits with predictable modal behavior.

### Digitally controlled and boosted PRET using molecular addressability

Lastly, we increased the efficiency of PRET with our molecularly addressable optical nanocircuits. In particular, we contrasted PRET efficiencies between monomeric and dimeric optical nanocircuits assembled with 100 nm Au NSs (Fig. 6A), revealing a large difference in electric field confinement and light-molecule coupling (Fig. 6, B and C). To achieve this, we experimentally and theoretically quantified the threshold molecular loading of YOYO-3 (i.e., *R_nrad_*), beyond which PRET can be induced. To precisely control the amount of YOYO-3 loading, we immobilized the Au NS monomers and assembled symmetric Au NS dimers in a microfluidic chamber and adjusted the concentration of YOYO-3 dye molecules (see Supplementary Materials). The Au NS monomer was conjugated with double-stranded DNA (dsDNA) to facilitate the loading of YOYO-3 on the Au NS through the dye’s strong affinity for intercalation into dsDNA. In contrast, the Au NS surfaces in the dimeric circuits were modified with only ssDNA, such that the YOYO-3 dye would preferentially load on the dsDNA of the origami barrel for this sample. Importantly, our study required a very broad loading range, from only a few molecules to approximately 6500 per structure, in order to systematically evaluate PRET responses in monomeric versus dimeric configurations. Achieving such a wide dynamic range would not be feasible using conventional handle–anti-handle labeling strategies, which typically saturate at a few hundred dye molecules per origami (*48*). For this reason, we adopted an intercalation-based approach, which allowed reliable and deterministic adjustment of dye loading. As detailed in the Supplementary Materials, the number of intercalated YOYO-3 molecules bound to DNA duplexes can be precisely controlled, and our theoretical estimates closely match the experimentally measured values (*49*, *50*).

**Fig. 6.**
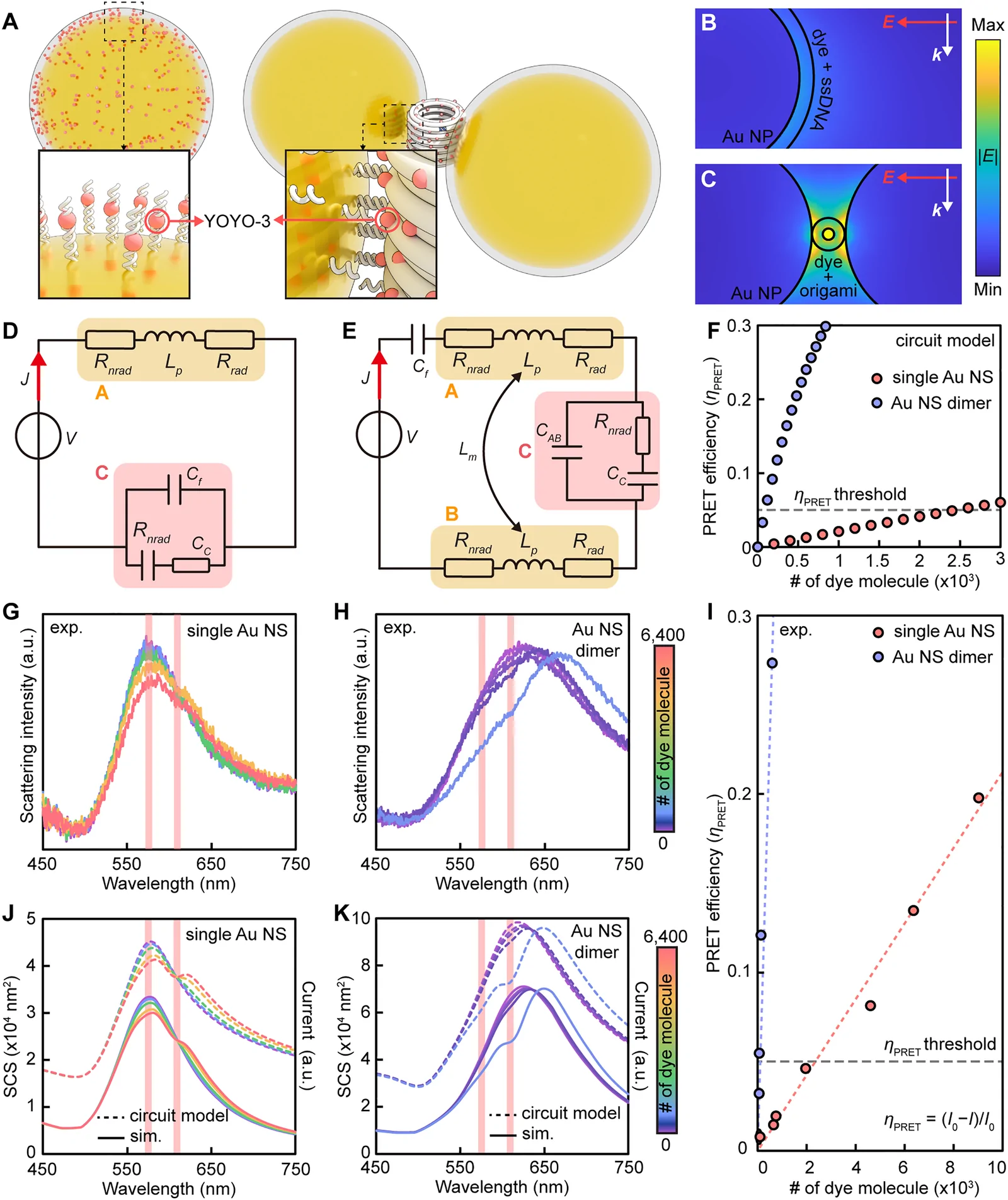
Plasmon resonance energy transfer (PRET) in molecularly addressable optical nanocircuits. (**A**) YOYO-3-loaded monomeric and dimeric architectures. (**B** and **C**) Simulated electric-field magnitude near a single Au NS and a dimer with a dye-loaded barrel in the nanogap. (**D** and **E**) Corresponding lumped-element models, with the dye-loaded DNA component represented as an *R-*coupled *C* element. (**F**) Circuit-model PRET efficiency versus dye number for the monomer and dimer. (**G** and **H**) Experimental DF spectra as YOYO-3 loading increases; the shaded band marks the dye-absorption region. (**I**) Extracted PRET efficiency versus dye number, showing an approximately 100-fold lower dye requirement for the dimer to produce a comparable spectral dip. (**J** and **K**) Circulating-current spectra from circuit-model and SCS from full-wave simulation for different dye loadings. The model and simulation reproduce the measured thresholds and trends.

To fairly compare monomeric and dimeric PRET responses, the number of YOYO-3 molecules residing within the near-field region needed to be as similar as possible in both configurations. For a given total amount of duplex DNA, the number of intercalated YOYO-3 molecules remains effectively constant regardless of whether the duplexes exist as free dsDNA, are conjugated to an Au NS, or are incorporated into the DNA origami barrel scaffold. This allows us to ensure equal YOYO-3 loading across the two nanocircuit motifs. It is also important to note that the spatial distribution of the electromagnetic field differs fundamentally between the two configurations. In the dimeric optical nanocircuit, the near field is strongly confined within the nanogap created by capacitive coupling between the two Au NSs, leading to highly localized light–matter interactions. In contrast, the monomeric nanocircuit produces an extended near-field pattern distributed across the entire Au NS surface. For this reason, YOYO-3 dyes must be uniformly distributed over the monomeric NS surface to maximize interaction with the broader near-field region. Taken together, these considerations justify our experimental design, in which PRET behavior was evaluated by comparing a YOYO-3-loaded single Au NS with a YOYO-3-loaded dimeric system templated by DNA origami, ensuring both structural and optical comparability between the two cases.

The Thevenin equivalent nanocircuit diagrams for dye-loaded monomeric and dimeric circuits are shown in Fig. 6, D and E, respectively. Note that the sub-circuit, where *R_nrad_* is connected with *C_C_* and *C_f_* or *C_AB_*, needs to be additionally connected in series with *L*. The *R_nrad_* and *C_C_* values were determined by modeling the dsDNA shell of the Au NS monomer and the barrel of the Au NS dimer as effective spheres with varying amounts of YOYO-3 loading (see Supplementary Materials). According to the nanocircuit model (Fig. 6, D and E), the YOYO-3 dyes introduce a non-radiative loss channel into the plasmonic resonator. This manifests as a distinct dip in the scattering spectra. We therefore define the PRET-threshold as the minimum number required to produce a spectral dip that is reliably detectable above our measurement noise (corresponding to a PRET efficiency ∼0.04). For dimers, this threshold was predicted to be ∼60 molecules, which was much lower than the ∼2500 predicted for monomers (Fig. 6F) (circuit parameters summarized in table S6, Supplementary Materials). This theoretical prediction was confirmed using DF microspectroscopy (Fig. 6, G to I). Here, a clear PRET dip was observed with ∼70 YOYO-3 molecules for the dimeric nanocircuit, whereas the monomeric counterpart required a 100-fold greater number of molecules (∼ 6400) to produce a comparable spectral dip, in agreement with both FEM- and nanocircuit model-based theoretical predictions (Fig. 6, J and K).

## DISCUSSION

Taken together, our study has achieved three key advances. First, we demonstrated that DNA origami and Au NSs can each be interpreted as idealized lumped optical circuit elements, enabling the programmable and modular design of plasmonic resonators. This conceptually bridges molecular nanofabrication with circuit-based photonic design, offering a promising framework for building functional optical nanostructures with molecular precision.

Second, the three-layer barrel enabled geometry-resolved assembly of optical nanocircuits supporting bright ED/MD modes and a dark-mode Fano resonance. We distinguish three evidence levels: oxDNA and Au-NS-free TEM characterize intrinsic barrel stability; individual projected Au NS positions and gaps characterize loaded dry-state geometry; and paired TEM/DF spectra test the optical behavior of specific devices. Finite rotation at the handle–anti-handle duplex can occur and is not claimed to be eliminated. It is reduced and constrained by the low-fluctuation three-layer barrel, the larger contact footprint and multivalent anchoring of 100 nm Au NSs, and, where used, a second barrel. Direct segmentation of the central barrel in 100 nm Au NS assemblies is not reliable because the mass-thickness contrast of two to four adjacent Au NSs overwhelms the DNA signal in projection. We therefore use the spatial distribution of the Au NSs—particularly edge-to-edge gaps and center-position residuals—as the operational readout of loaded geometry. Expanded individual-particle statistics (fig. S28 and table S7, Supplementary Materials, section S22) and the nominal-versus-dry-state gap comparison (Supplementary Materials, section S28), together with one-to-one TEM/DF data, support this constrained, but not perfectly invariant, structural picture. The ultrasmooth, highly spherical Au NSs further reduce particle-shape variability relative to randomly faceted particles used in many previous DNA-origami plasmonic assemblies (*41*, *51*–*53*). This combination enabled the observed high-*Q* visible resonances without requiring a claim of a perfect macroscopic yield, while demonstrating high structural accuracy and high optical fidelity across the individual stoichiometrically correct devices examined here.

Third, the molecular addressability enabled by DNA origami substantially enhanced the interaction between dye molecules loaded onto DNA origami and plasmonic clusters, greatly increasing PRET efficiency. In the dimeric optical nanocircuit, PRET was observed with a few tens of dye molecules, requiring approximately 100-fold fewer molecules than the monomeric counterpart to produce a comparable spectral dip. This demonstrates the potential for low-copy-number molecular detection beyond conventional PRET-based sensing (*11*, *12*, *54*).

While this work highlights several representative motifs (Figs. 2 to 6) of higher-order optical nanocircuits, our approach is broadly extensible. By varying the size of the Au NSs and reprogramming the arrangement of ssDNA handles on the DNA origami barrel, a vast array of nanocircuit geometries can be constructed, enabling continuous increases in structural and functional complexity.

Thus, our DNA origami–based strategy enables access to optical resonances that have been challenging to realize through bottom-up assembly, owing to its capacity for precise and modular control at the molecular level. This level of addressability supports applications such as PRET-based spectroscopy and other molecule–plasmon interaction studies, where deterministic nanoscale spacing and well-defined geometries are critically important. Moreover, the ability to tune optical characteristics, including *Q*-factors, PRET responses, and Fano line shapes, facilitates highly controlled light confinement in nanoscale volumes. While these capabilities are distinct from and complementary to those offered by top-down nanofabrication, they provide a complementary route for integrating molecular components directly into optical architectures with nanometer precision. In particular, when combined with emerging DNA-origami placement and lithographic registration techniques (*55*–*58*), our approach offers a promising framework for positioning quantum emitters or other optically active molecules in designed plasmonic environments on chip. Such integration may enable hybrid quantum systems and scalable quantum optical architectures where molecular-level precision plays a central role.

## MATERIALS AND METHODS

### Folding DNA origami

Barrel-shaped DNA origami structures were folded using a p7249 scaffold ssDNA strand (Guild Bioscience Inc.) and a set of complementary staple ssDNA strands (Bioneer) (*21*, *59*). The folding mixture was prepared with 20 nM scaffold, a 10-fold excess of staple strands (200 nM), 16 mM MgCl_2_, 5 mM Tris-HCl (pH 8.0), and 1 mM EDTA. This mixture was thermally annealed in a Tetrad 2 Peltier thermal cycler (Bio-Rad) using the following protocol: an initial step at 80°C for 15 minutes, followed by a slow cooling ramp from 60°C to 24°C over 36 hours, and a final hold at 4°C. After annealing, the folded DNA origami structures were purified by agarose gel electrophoresis. Samples were mixed with a 6X agarose gel loading buffer (Bioneer) and loaded onto a 1.5 wt % agarose gel prepared with 0.5X Tris-Borate-EDTA (TBE) buffer (Sigma-Aldrich) and 10 mM MgCl_2_. Electrophoresis was performed in a horizontal system (Bio-Rad) at 60 V for 3 hours. The desired bands, visualized with SYBR Gold Nucleic Acid Gel Stain, were excised from the gel. Finally, the purified origami structures were extracted from the gel slices using Freeze ‘N Squeeze DNA Gel Extraction Spin Columns (Bio-Rad).

### Synthesis of Au NSs

Au NSs with diameters of 40 nm and 100 nm were synthesized using a multi-step method based on iterative reductive growth and oxidative dissolution (*33*–*35*). The process began with the preparation of an Au seed solution by reducing Gold (III) chloride trihydrate (HAuCl_4_) with ice-cold sodium borohydride (NaBH_4_). These seeds were then used to grow single-crystal Au nanorods (NRs) in the presence of silver nitrate (AgNO_3_) as a catalyst and L-ascorbic acid as a reducing agent. The core of the method involves an iterative growth and dissolution process. The Au NRs were first subjected to an oxidative etching step with a controlled concentration of HAuCl_4_ to form intermediate seeds. These seeds were subsequently used for a further growth step with L-ascorbic acid to form concave rhombic dodecahedra (CRDs). A final etching step, again with HAuCl_4_, transformed the CRDs into highly uniform and spherical 40 nm Au NSs. To achieve the larger 100 nm size, this growth-and-etch cycle was repeated using the 40 nm Au NSs as seeds. The complete, detailed protocol is available in the Supplementary Materials.

### Preparation of DNA functionalized Au NSs

The surfaces of the Au NSs were functionalized with thiolated anti-handle ssDNA, which is complementary to the handle strands on the DNA origami scaffold. After washing the as-synthesized Au NSs to remove residual surfactant, they were incubated with the thiolated DNA. To maximize the DNA loading density on the nanoparticle surface, a gradual salt-aging process was employed over two days, during which the salt concentration was slowly increased to 750 mM to screen electrostatic repulsion (*35*, *60*, *61*). Successful conjugation was confirmed by observing a characteristic redshift in the plasmon resonance peak of the Au NSs. Finally, the DNA-functionalized Au NSs were washed via centrifugation to remove excess, unbound DNA strands before use. The full, detailed protocol is available in the Supplementary Materials.

### Assembly and purification of Au NS clusters

To assemble the Au NS clusters, the washed DNA-functionalized Au NSs (DNA-Au NSs) were first redispersed in a buffer containing 10 mM MgCl_2_, 0.05 wt % SDS, and 10 mM Tris-HCl. These DNA-Au NSs were then mixed with purified barrel DNA origami. The stoichiometric ratio of DNA-Au NSs to barrel DNA origami was set to 10:1, 15:1, and 20:1 for the assembly of dimers, trimers, and tetramers, respectively. These ratios were kept consistent regardless of the Au NS diameter. The concentration of the barrel DNA origami was fixed at 0.5 nM for all experiments. The resulting mixture was incubated overnight at room temperature in a mixer to facilitate cluster formation.

Following incubation, the assembled Au NS clusters were analyzed and purified by agarose gel electrophoresis, as shown in Figs. 3 and 5 of the main manuscript. A 0.85% agarose gel was prepared with a buffer containing 0.5X TBE, 10 mM MgCl_2_, and 1x SYBR Gold Nucleic Acid Gel Stain. Before loading, samples were mixed with a 6x gel loading buffer containing 40% sucrose, 0.05% bromophenol blue, and 0.05% xylene cyanol FF. Electrophoresis was performed at 60 V for 3 hours. The separated bands were visualized with a white LED lamp and imaged using a Gel Doc XR+ gel documentation system (Bio-Rad). The bands corresponding to the well-assembled Au NS clusters were then physically excised from the gel with a razor blade. Finally, the clusters were recovered from the gel slice by squeezing it between two glass slides covered with clean Parafilm.

#### TEM sample preparation for Au NS clusters characterization

To obtain sufficient surface density of the assembled nanocircuits for TEM characterization, a specialized sampling protocol was employed. Following purification via gel electrophoresis, the samples were stored at 4°C for at least 48 hours to allow for the sedimentation of the Au NS clusters. The supernatant was then carefully removed to increase the local concentration of the assemblies. TEM grids (carbon-supported) were pre-cleaned via O_2_ plasma treatment (i.e., glow discharge) immediately prior to use. 2.5 μL of the concentrated sample was placed onto the grid and incubated for 5 minutes to facilitate the adsorption of the structures. Subsequently, 2.0 μL of the solution was carefully withdrawn using a micropipette, leaving a residual thin film of solution on the grid surface. The grids were then allowed to air-dry completely. This method minimized the potential loss or displacement of assemblies that often occurs with complete liquid removal and produced a high surface density of Au NS clusters for TEM imaging. Supporting analyses are organized in the Supplementary Materials as follows: image alignment and processing (section S25); nominal and device-specific geometries and gap-reading sensitivity (sections S26 to S27, figs. S33 to S35, and table S8); loaded-state gap geometry, dataset denominators, and mode-deviant examples (sections S28 to S29, figs. S36 to S38, and tables S9 and S10); individual barrel dimensions and TEM fixed-pattern controls (sections S30 to S1, figs. S39 to S41, and table S11); and gap/dielectric sensitivity (section S32 and fig. S42).

### TEM image analysis and gap quantification

High-magnification TEM micrographs of isolated Au NS assemblies were acquired at 80,000x magnification (calibrated sampling: 0.604 nm/pixel). For planar assemblies (dimers, trimers, and tetramers), interparticle edge-to-edge gaps were quantified from line intensity profiles across the narrowest interparticle gap in Fiji (ImageJ), with boundaries assigned to the maximum intensity gradient. Because line-profile interpolation across the pixel array enables sub-pixel edge localization, the maximum gradient inflection points were determined at sub-pixel resolution. Five technical gap-reading replicates were performed by one operator on the same TEM image, with the preceding numerical values hidden until all measurements were completed (Supplementary Materials, section S27). The resulting standard deviations of 0.1–0.4 nm quantify analysis repeatability under this protocol rather than the instrumental resolution of the TEM. For the non-planar pentamer, 3D-to-2D projection matching was employed: a 3D coordinate model with uniform surface-to-surface gaps was rotated across three Euler angles until its orthographic projection matched the experimental TEM contour.

### Polarization-resolved DF scattering spectra measurement

The scattering spectra of individual nanoclusters were measured using a custom-built DF microspectroscopy setup. To enable direct correlation between the geometry of each cluster and its optical response, samples were placed on a Formvar-coated TEM grid, which allowed for TEM imaging of a specific cluster before its spectrum was measured. To investigate the resonant scattering behavior of the Au NS assemblies upon differently polarized light illumination, polarization-resolved spectra were acquired by rotating a linear polarizer in the illumination and detection path relative to the cluster’s main axis. For the PRET experiments, a microfluidic chamber was used to immobilize the nanostructures on a glass surface, which permitted the in-situ exchange of solutions with varying dye concentrations during spectral acquisition. The full details of the optical setup and sample preparation are provided in the Supplementary Materials.

### Estimation of YOYO-3 loading

The number of YOYO-3 dye molecules loaded onto the DNA nanostructures was estimated using the McGhee-von Hippel model (*49*). To use this model accurately, we first experimentally determined the association constant of the dye (*K_a_*) for our specific buffer conditions. This was achieved by measuring the absorption spectra of solutions containing a fixed concentration of dsDNA that were titrated with varying amounts of YOYO-3. The concentration of bound dye at each step was quantified by deconvoluting the distinct spectral contributions of the free and bound dye populations. The resulting *K*_a_ value was then used to precisely calculate the dye loading for the main PRET experiments. The full details of this procedure are provided in the Supplementary Materials.

### FEM-based electromagnetic numerical calculation

The optical properties of the Au NS clusters were simulated using the finite element method in CST Microwave Studio. For clarity, three geometric quantities are distinguished throughout this work: the nominal design gap derived from barrel/handle geometry; the population-level projected dry-state TEM gap obtained from 100 stoichiometrically target-positive structures; and the device-specific FEM input gap measured from the same individual nanocircuit used for the corresponding DF spectrum. The FEM geometry, including each particle diameter and interparticle gap, was taken from the third quantity rather than from an ensemble-averaged schematic. The one-to-one TEM geometries and numerical inputs are provided in Supplementary Materials, section S27, fig. S34, and table S8. The clusters were illuminated with a polarization-controlled plane wave at a 53° angle of incidence. The dielectric function of Au was taken from literature values (*62*). For dry-state DF simulations on Formvar-coated TEM grids, the ambient environment was modeled with an effective background permittivity of *ε*_eff_ = 1.56 (*n*_eff_ ≈ 1.25), whereas a rounded effective permittivity of ε_eff_ ≈ 2.00 was used for in situ PRET simulations in aqueous microfluidic channels. The local dielectric shell comprising the DNA origami template and ssDNA handles was parameterized from their molecular compositions. For PRET simulations, dye-loaded DNA components were modeled with a Lorentzian oscillator function parameterized from the experimental absorption spectra. Full simulation parameters, dielectric models, and sensitivity analyses are provided in Supplementary Materials, Sections 7 and 32.

### Analytical analysis of optical nanocircuit model

To analyze the scattering and resonant behavior of the Au NS clusters and the PRET system, an optical nanocircuit model was used based on established frameworks (*14*, *17*). Following the simulated electric displacement fields, individual Au NSs were modeled as an inductor (*L*) with radiative and non-radiative resistors (*R*), while the dye-loaded DNA origami was modeled as a lossy capacitor (*R*_nrad_-*C* component). These components were integrated into a larger circuit to model a full cluster, with additional capacitors and mutual inductors accounting for the near-field coupling between nanoparticles. The final scattering response of the system was then estimated by calculating the total current flowing through the equivalent circuit. The specific details for parameter determination and all circuit parameter values are summarized in the Supplementary Materials (tables S2 to S6).

### DNA-PAINT analysis

For DNA-PAINT imaging, a custom-built total internal reflection fluorescence (TIRF) microscope was used. For imaging, the barrel DNA origami was functionalized with docking strands for super-resolution imaging and with biotin molecules for surface attachment. The samples were immobilized within a fluidic chamber via a standard biotin-streptavidin linkage. Imaging was performed by introducing a solution containing 5 nM of fluorescently-labeled imager strands, which are complementary to the docking strands on the origami. The transient binding of these imager strands creates the blinking signal required for super-resolution imaging. Image sequences of 10,000 frames were recorded and then processed using the Picasso software package to reconstruct the final images from the accumulated single-molecule localizations (*63*). The full details of the optical setup and imaging parameters are provided in the Supplementary Materials.

## Supplementary Materials

### This PDF file includes:

Supplementary Text Figs. S1 to S42 Tables S1 to S11 References

## References

[1] H. Xin, B. Namgung, L. P. Lee, Nanoplasmonic optical antennas for life sciences and medicine. Nat. Rev. Mater. 3, 228–243 (2018).

[2] M. Kauranen, A. V. Zayats, Nonlinear plasmonics. Nat. Photonics 6, 737–748 (2012).

[3] J. Butet, P.-F. Brevet, O. J. F. Martin, Optical second harmonic generation in plasmonic nanostructures: From fundamental principles to advanced applications. ACS Nano 9, 10545–10562 (2015). 10.1021/acsnano.5b04373 26474346

[4] C. Lee, B. Lawrie, R. Pooser, K. G. Lee, C. Rockstuhl, M. Tame, Quantum plasmonic sensors. Chem. Rev. 121, 4743–4804 (2021). 10.1021/acs.chemrev.0c01028 33787252

[5] J.-M. Nam, J.-W. Oh, H. Lee, Y. D. Suh, Plasmonic nanogap-enhanced raman scattering with nanoparticles. Acc. Chem. Res. 49, 2746–2755 (2016). 10.1021/acs.accounts.6b00409 27993009

[6] J. Langer, D. Jimenez De Aberasturi, J. Aizpurua, R. A. Alvarez-Puebla, B. Auguié, J. J. Baumberg, G. C. Bazan, S. E. J. Bell, A. Boisen, A. G. Brolo, J. Choo, D. Cialla-May, V. Deckert, L. Fabris, K. Faulds, F. J. García De Abajo, R. Goodacre, D. Graham, A. J. Haes, C. L. Haynes, C. Huck, T. Itoh, M. Käll, J. Kneipp, N. A. Kotov, H. Kuang, E. C. Le Ru, H. K. Lee, J.-F. Li, X. Y. Ling, S. A. Maier, T. Mayerhöfer, M. Moskovits, K. Murakoshi, J.-M. Nam, S. Nie, Y. Ozaki, I. Pastoriza-Santos, J. Perez-Juste, J. Popp, A. Pucci, S. Reich, B. Ren, G. C. Schatz, T. Shegai, S. Schlücker, L.-L. Tay, K. G. Thomas, Z.-Q. Tian, R. P. Van Duyne, T. Vo-Dinh, Y. Wang, K. A. Willets, C. Xu, H. Xu, Y. Xu, Y. S. Yamamoto, B. Zhao, L. M. Liz-Marzán, Present and future of surface-enhanced raman scattering. ACS Nano 14, 28–117 (2020). 10.1021/acsnano.9b04224 PMC6990571 31478375

[7] A. Alu, N. Engheta, The quest for magnetic plasmons at optical frequencies. Opt. Express 17, 5723–5730 (2009). 10.1364/oe.17.005723 19333341

[8] J. A. Fan, C. Wu, K. Bao, J. Bao, R. Bardhan, N. J. Halas, V. N. Manoharan, P. Nordlander, G. Shvets, F. Capasso, Self-assembled plasmonic nanoparticle clusters. Science 328, 1135–1138 (2010). 10.1126/science.1187949 20508125

[9] J. Lee, J.-H. Huh, K. Kim, S. Lee, DNA origami-guided assembly of the roundest 60-100 nm gold nanospheres into plasmonic metamolecules. Adv. Funct. Mater. 28, 1707309 (2018).

[10] J. H. Huh, K. Kim, E. Im, J. Lee, Y. D. Cho, S. Lee, Exploiting colloidal metamaterials for achieving unnatural optical refractions. Adv. Mater. 32, e2001806 (2020). 10.1002/adma.202001806 33079414

[11] G. L. Liu, Y.-T. Long, Y. Choi, T. Kang, L. P. Lee, Quantized plasmon quenching dips nanospectroscopy via plasmon resonance energy transfer. Nat. Methods 4, 1015–1017 (2007). 10.1038/nmeth1133 18026109

[12] Y. Choi, T. Kang, L. P. Lee, Plasmon resonance energy transfer (PRET)-based molecular imaging of cytochrome C in living cells. Nano Lett. 9, 85–90 (2009). 10.1021/nl802511z PMC2771226 19093833

[13] G. M. Akselrod, C. Argyropoulos, T. B. Hoang, C. Ciracì, C. Fang, J. Huang, D. R. Smith, M. H. Mikkelsen, Probing the mechanisms of large Purcell enhancement in plasmonic nanoantennas. Nat. Photonics 8, 835–840 (2014).

[14] N. Engheta, A. Salandrino, A. Alù, Circuit elements at optical frequencies: Nanoinductors, nanocapacitors, and nanoresistors. Phys. Rev. Lett. 95, 095504 (2005). 10.1103/PhysRevLett.95.095504 16197226

[15] N. Engheta, Circuits with light at nanoscales: Optical nanocircuits. Science 317, 1698–1702 (2007). 10.1126/science.1133268 17885123

[16] Y. Sun, B. Edwards, A. Alù, N. Engheta, Experimental realization of optical lumped nanocircuits at infrared wavelengths. Nat. Mater. 11, 208–212 (2012). 10.1038/nmat3230 22286335

[17] J. Shi, F. Monticone, S. Elias, Y. Wu, D. Ratchford, X. Li, A. Alù, Modular assembly of optical nanocircuits. Nat. Commun. 5, 3896 (2014). 10.1038/ncomms4896 24871450

[18] P. W. K. Rothemund, Folding DNA to create nanoscale shapes and patterns. Nature 440, 297–302 (2006). 10.1038/nature04586 16541064

[19] S. M. Douglas, H. Dietz, T. Liedl, B. Högberg, F. Graf, W. M. Shih, Self-assembly of DNA into nanoscale three-dimensional shapes. Nature 459, 414–418 (2009). 10.1038/nature08016 PMC2688462 19458720

[20] S. Dey, C. Fan, K. V. Gothelf, J. Li, C. Lin, L. Liu, N. Liu, M. A. D. Nijenhuis, B. Saccà, F. C. Simmel, H. Yan, P. Zhan, DNA origami. Nat. Rev. Methods Primer 1, 13 (2021).

[21] S. F. J. Wickham, A. Auer, J. Min, N. Ponnuswamy, J. B. Woehrstein, F. Schueder, M. T. Strauss, J. Schnitzbauer, B. Nathwani, Z. Zhao, S. D. Perrault, J. Hahn, S. Lee, M. M. Bastings, S. W. Helmig, A. L. Kodal, P. Yin, R. Jungmann, W. M. Shih, Complex multicomponent patterns rendered on a 3D DNA-barrel pegboard. Nat. Commun. 11, 5768 (2020). 10.1038/s41467-020-18910-x PMC7666213 33188187

[22] J. Lee, J. Kim, G. Posnjak, A. Ershova, D. Hayakawa, W. M. Shih, W. B. Rogers, Y. Ke, T. Liedl, S. Lee, DNA origami colloidal crystals: Opportunities and challenges. Nano Lett. 25, 16–27 (2025). 10.1021/acs.nanolett.4c05041 39704025

[23] F. N. Gür, F. W. Schwarz, J. Ye, S. Diez, T. L. Schmidt, Toward self-assembled plasmonic devices: High-yield arrangement of gold nanoparticles on DNA origami templates. ACS Nano 10, 5374–5382 (2016). 10.1021/acsnano.6b01537 27159647

[24] C. Hartl, K. Frank, H. Amenitsch, S. Fischer, T. Liedl, B. Nickel, Position accuracy of gold nanoparticles on DNA origami structures studied with small-angle X-ray Scattering. Nano Lett. 18, 2609–2615 (2018). 10.1021/acs.nanolett.8b00412 PMC6544511 29498287

[25] Z. Zhao, E. L. Jacovetty, Y. Liu, H. Yan, Encapsulation of gold nanoparticles in a DNA origami cage. Angew. Chem. Int. Ed. Engl. 50, 2041–2044 (2011). 10.1002/anie.201006818 PMC3083863 21344547

[26] R. Yaadav, K. Trofymchuk, M. Dass, V. Behrendt, B. Hauer, J. Schütz, C. Close, M. Scheckenbach, G. Ferrari, L. Mäurer, S. Sebina, V. Glembockyte, T. Liedl, P. Tinnefeld, Bringing attomolar detection to the point-of-care with nanopatterned DNA origami nanoantennas. Adv. Mater. 37, e07407 (2025). 10.1002/adma.202507407 PMC12510279 40714742

[27] N. Siegel, M. Sanz-Paz, J. González-Colsa, G. Serrera, F. Zhu, A. M. Szalai, K. Kołątaj, M. Fujii, H. Sugimoto, P. Albella, G. P. Acuna, Distance-dependent interaction between a single emitter and a single dielectric nanoparticle using DNA origami. Small Struct. 6, 2500299 (2025).

[28] K. Trofymchuk, V. Glembockyte, L. Grabenhorst, F. Steiner, C. Vietz, C. Close, M. Pfeiffer, L. Richter, M. L. Schütte, F. Selbach, R. Yaadav, J. Zähringer, Q. Wei, A. Ozcan, B. Lalkens, G. P. Acuna, P. Tinnefeld, Addressable nanoantennas with cleared hotspots for single-molecule detection on a portable smartphone microscope. Nat. Commun. 12, 950 (2021). 10.1038/s41467-021-21238-9 PMC7878865 33574261

[29] K. Trofymchuk, K. Kołątaj, V. Glembockyte, F. Zhu, G. P. Acuna, T. Liedl, P. Tinnefeld, Gold nanorod DNA origami antennas for 3 orders of magnitude fluorescence enhancement in NIR. ACS Nano 17, 1327–1334 (2023). 10.1021/acsnano.2c09577 36594816

[30] B. Shen, M. A. Kostiainen, V. Linko, DNA origami nanophotonics and plasmonics at interfaces. Langmuir 34, 14911–14920 (2018). 10.1021/acs.langmuir.8b01843 PMC6291805 30122051

[31] Y. Zhang, A. C. Allen, Z. J. Petrek, H. H. Cao, D. Kumar, M. C. Goodlad, V. G. Martinez, J. Singh, J. Z. Zhang, T. Ye, Formation of linear plasmonic heterotrimers using nanoparticle docking to DNA Origami cages. J. Phys. Chem. C 128, 11699–11708 (2024). 10.1021/acs.jpcc.4c02229 PMC11264316 39050926

[32] W. Sun, E. Boulais, Y. Hakobyan, W. L. Wang, A. Guan, M. Bathe, P. Yin, Casting inorganic structures with DNA molds. Science 346, 1258361 (2014). 10.1126/science.1258361 PMC4260265 25301973

[33] J. Lee, S. Y. Lee, D.-K. Lim, D. J. Ahn, S. Lee, Antifreezing gold colloids. J. Am. Chem. Soc. 141, 18682–18693 (2019). 10.1021/jacs.9b05526 31618027

[34] T. M. Nguyen, Y. Cho, J.-H. Huh, H. Ahn, N. Kim, K. H. Rho, J. Lee, M. Kwon, S. H. Park, C. Kim, K. Kim, Y.-S. Kim, S. Lee, Ultralow-loss substrate for nanophotonic dark-field microscopy. Nano Lett. 23, 1546–1554 (2023). 10.1021/acs.nanolett.2c05030 36757958

[35] J. Lee, S. Lee, Non-invasive, reliable, and fast quantification of DNA loading on gold nanoparticles by a one-step optical measurement. Anal. Chem. 95, 1856–1866 (2023). 10.1021/acs.analchem.2c03378 36633590

[36] S. K. Balasubramanian, L. Yang, L.-Y. L. Yung, C.-N. Ong, W.-Y. Ong, L. E. Yu, Characterization, purification, and stability of gold nanoparticles. Biomaterials 31, 9023–9030 (2010). 10.1016/j.biomaterials.2010.08.012 20801502

[37] C. Ciracì, F. Vidal-Codina, D. Yoo, J. Peraire, S.-H. Oh, D. R. Smith, Impact of surface roughness in nanogap plasmonic systems. ACS Photonics 7, 908–913 (2020).

[38] N. Tanjeem, C. Chomette, N. B. Schade, S. Ravaine, E. Duguet, M. Tréguer-Delapierre, V. N. Manoharan, Polyhedral plasmonic nanoclusters through multi-step colloidal chemistry. Mater. Horiz. 8, 565–570 (2021). 10.1039/d0mh01311k 34821272

[39] F. Shafiei, F. Monticone, K. Q. Le, X. X. Liu, T. Hartsfield, A. Alù, X. Li, A subwavelength plasmonic metamolecule exhibiting magnetic-based optical Fano resonance. Nat. Nanotechnol. 8, 95–99 (2013). 10.1038/nnano.2012.249 23353675

[40] K. J. Park, J. H. Huh, D. W. Jung, J. S. Park, G. H. Choi, G. Lee, P. J. Yoo, H. G. Park, G. R. Yi, S. Lee, Assembly of “3D” plasmonic clusters by “2D” AFM nanomanipulation of highly uniform and smooth gold nanospheres. Sci. Rep. 7, 6045 (2017). 10.1038/s41598-017-06456-w PMC5519739 28729629

[41] W. Fang, S. Jia, J. Chao, L. Wang, X. Duan, H. Liu, Q. Li, X. Zuo, L. Wang, L. Wang, N. Liu, C. Fan, Quantizing single-molecule surface-enhanced Raman scattering with DNA origami metamolecules. Sci. Adv. 5, eaau4506 (2019). 10.1126/sciadv.aau4506 PMC6764828 31598548

[42] K. F. Wagenbauer, C. H. Wachauf, H. Dietz, Quantifying quality in DNA self-assembly. Nat. Commun. 5, 3691 (2014). 10.1038/ncomms4691 PMC3997801 24751596

[43] T. Gerling, K. F. Wagenbauer, A. M. Neuner, H. Dietz, Dynamic DNA devices and assemblies formed by shape-complementary, non–base pairing 3D components. Science 347, 1446–1452 (2015). 10.1126/science.aaa5372 25814577

[44] J. J. Funke, H. Dietz, Placing molecules with Bohr radius resolution using DNA origami. Nat. Nanotechnol. 11, 47–52 (2016). 10.1038/nnano.2015.240 26479026

[45] D. R. Smith, J. B. Pendry, M. C. K. Wiltshire, Metamaterials and Negative Refractive Index. Science 305, 788–792 (2004). 10.1126/science.1096796 15297655

[46] M. F. Limonov, M. V. Rybin, A. N. Poddubny, Y. S. Kivshar, Fano resonances in photonics. Nat. Photonics 11, 543–554 (2017).

[47] J. A. Fan, K. Bao, C. Wu, J. Bao, R. Bardhan, N. J. Halas, V. N. Manoharan, G. Shvets, P. Nordlander, F. Capasso, Fano-like interference in self-assembled plasmonic quadrumer clusters. Nano Lett. 10, 4680–4685 (2010). 10.1021/nl1029732 20923179

[48] J. B. Woehrstein, M. T. Strauss, L. L. Ong, B. Wei, D. Y. Zhang, R. Jungmann, P. Yin, Sub–100-nm metafluorophores with digitally tunable optical properties self-assembled from DNA. Sci. Adv. 3, e1602128 (2017). 10.1126/sciadv.1602128 PMC5479647 28691083

[49] K. Gunther, M. Mertig, R. Seidel, Mechanical and structural properties of YOYO-1 complexed DNA. Nucleic Acids Res. 38, 6526–6532 (2010). 10.1093/nar/gkq434 PMC2965214 20511588

[50] S. G. Lopez, M. J. Ruedas-Rama, S. Casares, J. M. Alvarez-Pez, A. Orte, Bulk and single-molecule fluorescence studies of the saturation of the DNA double helix using YOYO-3 intercalator dye. J. Phys. Chem. B 116, 11561–11569 (2012). 10.1021/jp303438d 22947035

[51] E. M. Roller, L. K. Khorashad, M. Fedoruk, R. Schreiber, A. O. Govorov, T. Liedl, DNA-assembled nanoparticle rings exhibit electric and magnetic resonances at visible frequencies. Nano Lett. 15, 1368–1373 (2015). 10.1021/nl5046473 PMC4415903 25611357

[52] N. Liu, T. Liedl, DNA-assembled advanced plasmonic architectures. Chem. Rev. 118, 3032–3053 (2018). 10.1021/acs.chemrev.7b00225 PMC6546600 29384370

[53] A. Kuzyk, R. Jungmann, G. P. Acuna, N. Liu, DNA origami route for nanophotonics. ACS Photonics 5, 1151–1163 (2018). 10.1021/acsphotonics.7b01580 PMC6156112 30271812

[54] J. Ma, M. X. Gao, H. Zuo, Y. F. Li, P. F. Gao, C. Z. Huang, Distance-dependence study of plasmon resonance energy transfer with DNA spacers. Anal. Chem. 92, 14278–14283 (2020). 10.1021/acs.analchem.0c03991 33027589

[55] S. H. Park, J. Lee, C. Kim, B. Kang, A. Gopinath, S. Lee, Selective binding of DNA origami on self-assembled monolayer-patterned gold substrate. ACS Mater. Lett. 7, 2565–2573 (2025).

[56] Y. Cho, S. H. Park, J.-H. Huh, A. Gopinath, S. Lee, DNA as grabbers and steerers of quantum emitters. Nanophotonics 12, 399–412 (2023). 10.1515/nanoph-2022-0602 PMC11501876 39635394

[57] A. Gopinath, C. Thachuk, A. Mitskovets, H. A. Atwater, D. Kirkpatrick, P. W. K. Rothemund, Absolute and arbitrary orientation of single-molecule shapes. Science 371, eabd6179 (2021). 10.1126/science.abd6179 33602826

[58] A. Gopinath, E. Miyazono, A. Faraon, P. W. K. Rothemund, Engineering and mapping nanocavity emission via precision placement of DNA origami. Nature 535, 401–405 (2016). 10.1038/nature18287 27398616

[59] B. Y. Lee, J. Lee, D. J. Ahn, S. Lee, M. K. Oh, Optimizing protein V untranslated region sequence in M13 phage for increased production of single-stranded DNA for origami. Nucleic Acids Res. 49, 6596–6603 (2021). 10.1093/nar/gkab455 PMC8216270 34110422

[60] B. Liu, P. Wu, Z. Huang, L. Ma, J. Liu, Bromide as a robust backfiller on gold for precise control of DNA conformation and high stability of spherical nucleic acids. J. Am. Chem. Soc. 140, 4499–4502 (2018). 10.1021/jacs.8b01510 29561603

[61] J. Lee, J.-H. Huh, S. Lee, DNA base pair stacking crystallization of gold colloids. Langmuir 36, 5118–5125 (2020). 10.1021/acs.langmuir.0c00239 32316734

[62] P. B. Johnson, R. W. Christy, Optical constants of the noble metals. Phys. Rev. B 6, 4370–4379 (1972).

[63] J. Schnitzbauer, M. T. Strauss, T. Schlichthaerle, F. Schueder, R. Jungmann, Super-resolution microscopy with DNA-PAINT. Nat. Protoc. 12, 1198–1228 (2017). 10.1038/nprot.2017.024 28518172

[64] Z. Qian, S. P. Hastings, C. Li, B. Edward, C. K. McGinn, N. Engheta, Z. Fakhraai, S.-J. Park, Raspberry-like metamolecules exhibiting strong magnetic resonances. ACS Nano 9, 1263–1270 (2015). 10.1021/nn5050678 25621502

[65] E. Poppleton, M. Matthies, D. Mandal, F. Romano, P. Šulc, L. Rovigatti, oxDNA: Coarse-grained simulations of nucleic acids made simple. J. Open Source Softw. 8, 4693 (2023).

[66] E. Poppleton, J. Bohlin, M. Matthies, S. Sharma, F. Zhang, P. Šulc, Design, optimization and analysis of large DNA and RNA nanostructures through interactive visualization, editing and molecular simulation. Nucleic Acids Res. 48, e72 (2020). 10.1093/nar/gkaa417 PMC7337935 32449920

[67] E.-M. Roller, L. V. Besteiro, C. Pupp, L. K. Khorashad, A. O. Govorov, T. Liedl, Hotspot-mediated non-dissipative and ultrafast plasmon passage. Nat. Phys. 13, 761–765 (2017). 10.1038/nphys4120 PMC5540180 28781603

[68] E. Ringe, J. M. McMahon, K. Sohn, C. Cobley, Y. Xia, J. Huang, G. C. Schatz, L. D. Marks, R. P. Van Duyne, Unraveling the effects of size, composition, and substrate on the localized surface plasmon resonance frequencies of gold and silver nanocubes: A systematic single-particle approach. J. Phys. Chem. C 114, 12511–12516 (2010).

[69] G. Kewes, F. Binkowski, S. Burger, L. Zschiedrich, O. Benson, Heuristic modeling of strong coupling in plasmonic resonators. ACS Photonics 5, 4089–4097 (2018).

