# Supplementary material for: 3D DNA origami-enabled molecularly addressable optical nanocircuit
Source: Sci Adv. 2026 Oct 7;12(41):eaeb2001. doi: 10.1126/sciadv.aeb2001 (PMC13644784; doi:10.1126/sciadv.aeb2001)
Supplement: Supplementary file 1 [file sciadv.aeb2001_sm.pdf]

Science Advances
AAAS

Supplementary Materials for

3D DNA origami-enabled molecularly addressable optical nanocircuit

Jaewon Lee *et al.*

Corresponding author: Shelley F. J. Wickham, shelley.wickham@sydney.edu.au;
William M. Shih, william.shih@wyss.harvard.edu; Seungwoo Lee, seungwoo@korea.ac.kr

Sci. Adv. **12**, eaeb2001 (2026)
DOI: 10.1126/sciadv.aeb2001

This PDF file includes:

Supplementary Text
Figs. S1 to S42
Tables S1 to S11
References

1. Barrel-shaped DNA origami

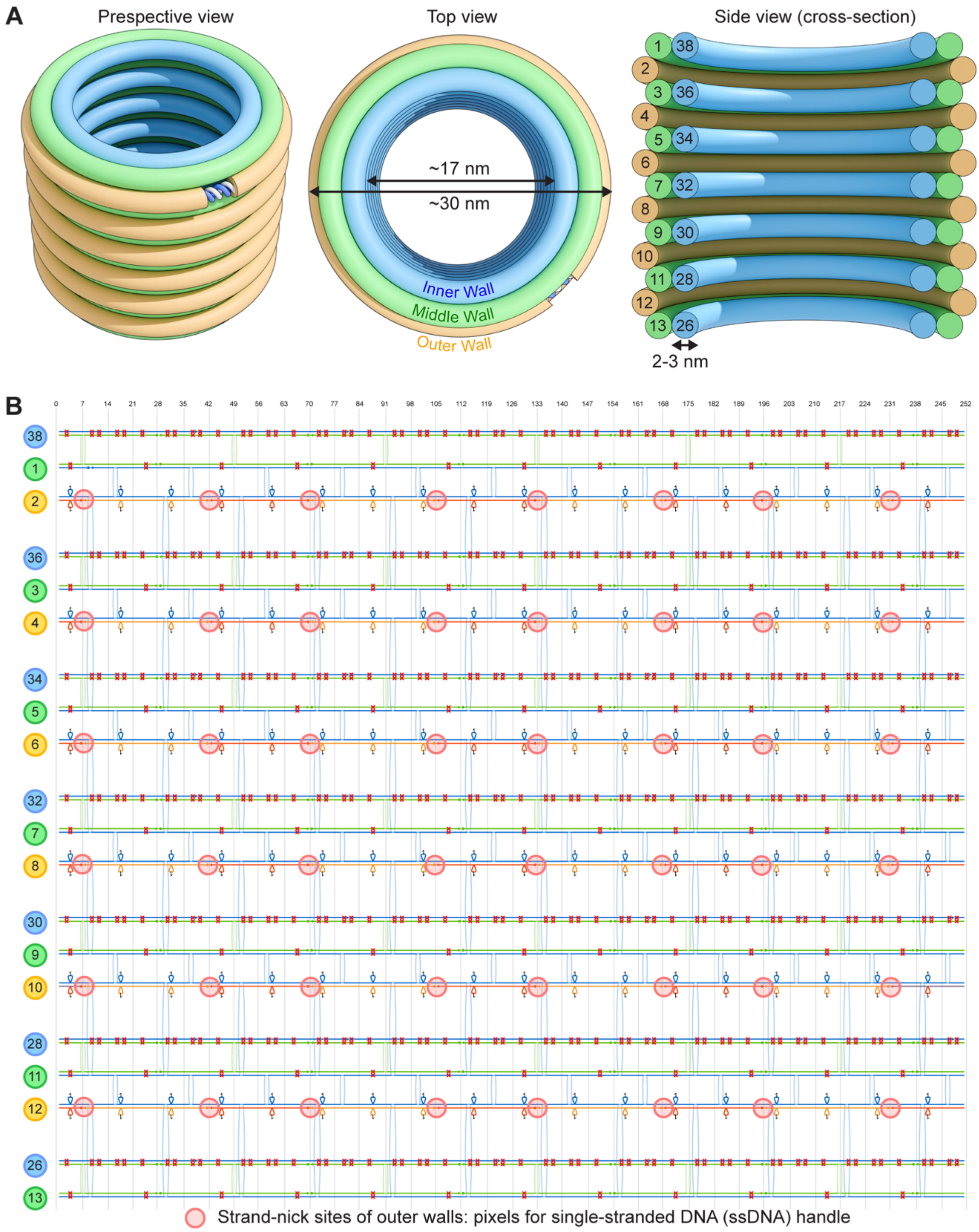

Fig. S1 | Design and schematic of the barrel DNA origami structure. (**A**) Schematics of the DNA origami barrel shown from a perspective, top, and side view. The structure consists of three concentric walls of double-stranded DNA (dsDNA) helices, shown as cylinders. The inner, middle, and outer walls are colored blue, green, and orange, respectively. (**B**) The caDNAno design scaffold and staple routing map for the barrel structure.

2. Coarse-grained molecular dynamics calculation of DNA origami

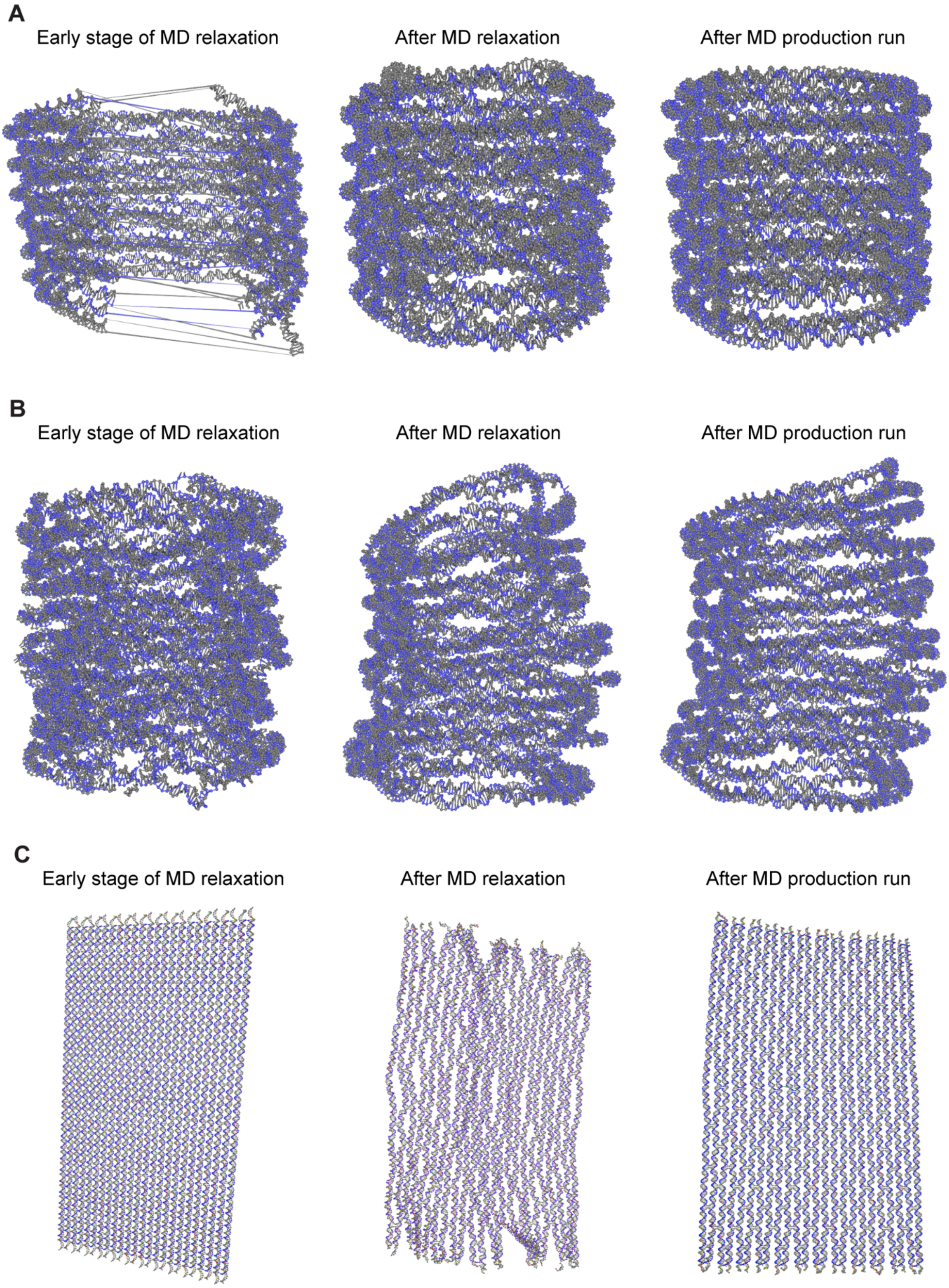

Fig. S2 | Coarse-grained molecular dynamics (MD) simulation process of DNA origami structures. (**A, B, C**) Representative snapshots from MD simulations using the coarse-grained oxDNA model. The panels show the configurations of three different DNA nanostructures: (**A**)

a three-layered barrel, (**B**) a two-layered barrel, and (**C**) a 2D rectangular DNA origami sheet. In these coarse-grained DNA models, the scaffold strand is colored blue and the staple strands are grey.

Coarse-grained molecular dynamics (MD) simulations were performed using the oxDNA simulation package and followed established standard protocols (*65*, *66*). The simulation workflow consists of several stages. First, initial structural configurations of the DNA origami were generated from scadnano design files. These initial models were relaxed using a Monte Carlo (MC) simulation. Next, the structures underwent a two-step MD process. An initial MD relaxation was performed with modified backbone forces to allow structural rearrangement (see the early stage of **Figure S2**). During this stage, a mutual trap potential was applied between base pairs to prevent the premature melting of double-stranded DNA (dsDNA) helices. After this initial relaxation, a final production run was conducted using the standard oxDNA force field without any external constraints (see the after MD relaxation and production run stages of **Figure S2**). Finally, the simulation trajectories from the production run were analyzed to calculate the root mean square fluctuation (RMSF) and determine the mean structural configuration using the built-in oxDNA analysis tools (*66*).

3. Design-level functional comparison with representative 3D DNA origami scaffolds for plasmonic nanoparticle assembly

Table S1 combines matched-series quantitative RMSF values with a qualitative, design-level comparison across unrelated DNA-origami architectures; it is not a matched quantitative rigidity benchmark among unrelated architectures. The 2D sheet, two-layer barrel, and three-layer barrel in **Figure 1B** form a controlled design series with matched dimensions and simulation protocols. Extending the same quantitative oxDNA comparison to unrelated published architectures would require harmonized dimensions, helix density, crossover topology, and simulation settings and is beyond the scope of this study. Accordingly, quantitative RMSF values are reported only for the matched structures simulated here; no RMSF value is inferred for literature motifs. The design-level comparison instead asks whether a motif integrates the functions required here: C_n-symmetric z-parallel exterior addressability, equivalent top/bottom modularity, a hollow molecular-loading volume, and support for integrating multiple 100 nm Au NSs.

Table S1 | Design-level functional comparison of representative DNA origami motifs used for plasmonic nanoparticle assembly.

Feature	Barrel (this work)	2D sheet / rectangle (Ref 21, 24)	3D molds / nanoparticle cages (Ref 25, 31, 32)	Packed multilayer bundles (Ref 23, 24, 28, 29)
Comparison level	Quantitative for the matched 2D sheet/barrels; qualitative across other architectures	Quantitative for the matched 2D sheet/barrels; qualitative across other architectures	Qualitative	Qualitative
Matched-condition RMSF	≤0.5 nm at the Au NS-binding sidewall	2–3 nm at the center (2D sheet)	Not evaluated under matched conditions	Not evaluated under matched conditions
C_n symmetric exterior addressability	Yes (C_4 used here)	No	Generally no continuous cylindrical C_n array	Generally facet- or line-based
z-parallel exterior handle rows	Yes	No	Design dependent	Design dependent
Equivalent top/bottom modularity	Yes	No	Design dependent	Design dependent
Hollow molecular-loading volume	Yes	No	Often available	Usually absent
Demonstrated assembly of multiple 100 nm Au NSs	Up to four 100 nm Au NSs	Not established in the cited examples	Not established in the cited examples	Not established in the cited examples

Barrel DNA origami (this work): The three-layer barrel integrates low matched-series RMSF at the attachment wall, C_n-symmetric z-parallel exterior pixels, top/bottom modularity, and a hollow molecular-loading core. This combination enables controlled-symmetry assembly of multiple 100 nm Au NSs and molecular loading for PRET.

2D sheet/rectangle templates: These motifs provide dense planar addressability. Their role here is as the open-sheet precursor in the controlled sheet-to-barrel design progression, not as a universal reference for all 2D origami.

Design-level advantage of the barrel for this study: a closed and multilayer-reinforced cylindrical wall, continuous angular addressability, compatibility with multiple large Au NSs, and a molecularly accessible interior.

3D cuboid molds or nanoparticle cages: These motifs can provide robust cavity or docking geometries and may be mechanically rigid, but they are generally optimized for encapsulation or molding rather than a continuous C_n-symmetric convex exterior with equivalent top/bottom interfaces.

Design-level advantage of the barrel for this study: equivalent angular H1–H4 sites with z-parallel exterior handles, permitting programmed symmetry/asymmetry and attachment of multiple 100 nm Au NSs around a convex wall while retaining a central molecular-loading volume.

Packed multilayer bundles: These architectures can be highly rigid, but their external handles are typically arranged on facets or linear surfaces rather than around a continuously addressable cylindrical wall.

Design-level advantage of the barrel for this study: vertically parallelized handles distributed around a cylindrical exterior, independently programmable top and bottom interfaces for hierarchical 3D modularity, and a molecularly accessible interior.

4. Structural characterizations of assembled barrel origami

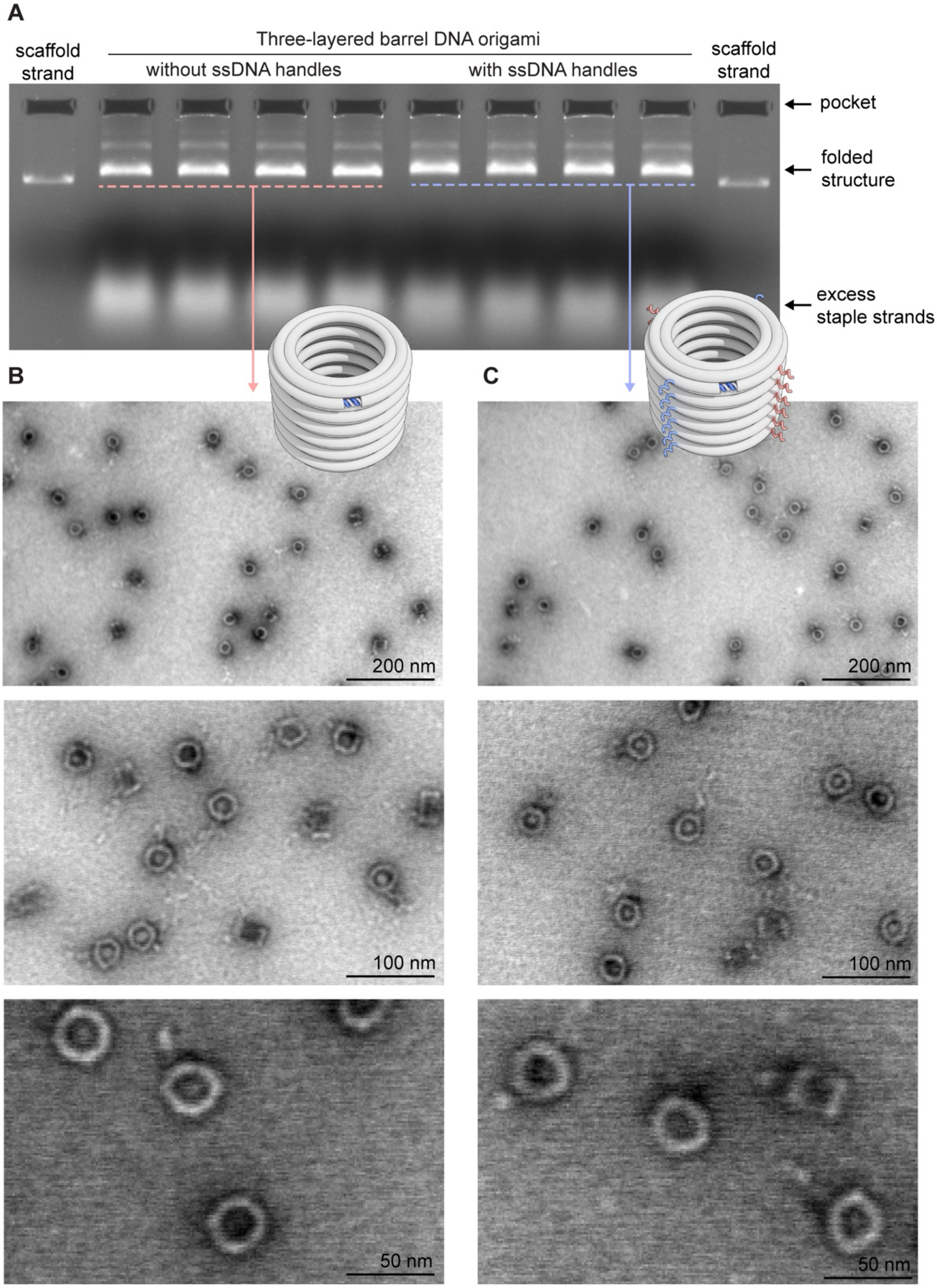

Fig. S3 | Scalable assembly of barrel DNA origami. (**A**) Agarose gel electrophoresis analysis of the folded DNA origami. The gel confirms the successful formation of barrels without single-stranded DNA (ssDNA) overhangs and barrels functionalized with ssDNA handles. (**B, C**) Representative low-magnification transmission electron microscopy (TEM) images of the assembled barrel DNA origami (**B**) without and (**C**) with ssDNA handles.

5. Estimation of YOYO-3 loading on DNA-conjugated gold nanospheres (DNA–Au NSs) and barrel DNA origami

To estimate and control the amount of YOYO-3 dye loaded onto the DNA nanostructures, we first experimentally determined the dye's binding affinity for our specific buffer conditions. The relationship between the dye concentration in solution and the amount bound to DNA can be described by the McGhee-von Hippel model (*51*):

$$\frac{r}{c_f} = K_a(1 - nr)\left[\frac{1 - nr}{1 - (n-1)r}\right]^{n-1} \tag{S1}$$

Here, r is the ratio of bound dye to DNA base pairs, c_f is the concentration of free dye in solution, K_a is the association constant, and n is the binding site size in base pairs (bp). The ratio r and the free dye concentration c_f can be expressed in terms of the total dye concentration (c_T), the bound dye concentration (c_b), and the total DNA base pair concentration (c_{DNA}):

$$c_b = c_T - c_f \tag{S2}$$

$$r = \frac{c_b}{c_{DNA}} = \frac{c_T - c_f}{c_{DNA}} \tag{S3}$$

$$\frac{c_T - c_f}{c_f c_{DNA}} = K_a\left(1 - n\frac{c_T - c_f}{c_{DNA}}\right)\left[\frac{1 - n\frac{c_T - c_f}{c_{DNA}}}{1 - (n-1)\frac{c_T - c_f}{c_{DNA}}}\right]^{n-1} \tag{S4}$$

Given a known binding site of n=3.22 bp/dye (*51*), the K_a was the only unknown parameter. Since K_a is highly sensitive to buffer conditions, we determined it experimentally.

To do this, we prepared solutions containing a fixed concentration of short (50 bp) double-stranded DNA (dsDNA) and titrated them with varying total concentrations of YOYO-3 (c_T). The c_b was measured using absorption spectroscopy. Free and bound YOYO-3 have distinct absorption spectra, allowing for their quantification (*52*). The bound-dye concentration (c_b) was determined by first deconvoluting each spectrum with a two-component Lorentzian fit. This resolved the overlapping spectral contributions from free and bound dye. The center wavelength of each fitted peak was then used to calculate the relative abundance of the two species and thereby determine c_b.

The experimentally measured values for c_b and c_f at each titration point were then fitted to the McGhee-von Hippel model (Equation S1) to extract the association constant. For our experimental buffer, we determined the value to be K_a=3.18×10^4 M^{-1}. This constant was then used to precisely estimate the YOYO-3 loading on the dsDNA-conjugated gold nanospheres (Au NSs) and the barrel DNA origami in our main experiments.

6. Analytical analysis of the optical nanocircuit model

To analytically analyze the scattering and resonant behavior of various Au NS clusters and the PRET system, we used an optical nanocircuit model based on previously established frameworks (*14*, *17*). The components were modeled based on the numerically simulated electric displacement field distribution depicted in Figure 1 of the main manuscript. A single Au NS was modeled as an inductor (L_p) coupled with a radiative resistor (R_{rad}) and a non-radiative resistor (R_{nrad}). The dye-loaded barrel DNA origami was modeled as a lossy capacitor, represented by a capacitor (C) coupled with a non-radiative resistor (R_{nrad}).

Following previous work, the impedance of the Au NS and dye-loaded DNA origami can be represented as follows (*14*):

$$Z_{\mathrm{NP}} = (-i\omega\varepsilon\pi R)^{-1} \tag{S5}$$

Here, ω is the angular frequency, ε is the permittivity, and R is the effective radius of the Au NS and DNA origami. The impedance for the displacement current of the fringe field can be represented as follows:

$$Z_{\mathrm{fringe}} = (-i\omega 2\pi R\varepsilon_0)^{-1} \tag{S6}$$

ε_0 is the background permittivity. To model Au NS clusters, the circuit elements were integrated based on the cluster configuration and the polarization of the illuminating light. For a cluster's fringe field impedance, the effective radius R was considered comparable to the cluster's dimension in the direction of the incident electric field. Additional capacitors (C) and mutual inductance (l_m) were introduced into the circuit to reflect the capacitive coupling between adjacent Au NSs. Because the measured scattered field can be related to the current delivered by the circuit's voltage source (*17*), the current flow in the final circuit was calculated to estimate the scattering and resonance behavior of the corresponding Au NS clusters and PRET systems. The nanocircuit parameters used for the lumped circuit elements and their assemblies are summarized in **Tables S2–S6**.

Table S2 | Nanocircuit parameters used for the dye-loaded DNA origami and an Au NS.

Motif	C_f (aF)	r (nm)	R_{rad} (ohms)
Au NS	9.56	51	7
Dye-loaded DNA origami	N/A	15	N/A

Table S3 | Nanocircuit parameters used for dimeric nanocircuits. (A) Symmetric dimers composed of two identical 100 nm Au NSs. **(B)** Asymmetric dimers composed of 100 and 40 nm Au NSs.

A

Polarization	C_f (aF)	r_A (nm)	r_B (nm)	$R_{rad,A}$ (ohms)	$R_{rad,B}$ (ohms)	l_m (fH)	C_{AB} (aF)
x-polarized	9.56	50.05	51.53	3.5	3.5	5	20

y-polarized	7.82	50.05	51.53	1	1	0	10

B

Polarization	C_f (aF)	r_A (nm)	r_B (nm)	$R_{rad,A}$ (ohms)	$R_{rad,B}$ (ohms)	l_m (fH)	C_{AB} (aF)
x-polarized	5.65	47.5	20	2	1	1	15
y-polarized	5.65	47.5	20	1	0.5	0	4

Table S4 | Nanocircuit parameters used for trimeric nanocircuits. (A) Mirror-symmetric heterogeneous trimers composed of two 100 nm Au NSs and one 40 nm Au NS. **(B)** Mirror-symmetric heterogeneous trimers composed of two 40 nm Au NSs and one 100 nm Au NS. **(C)** Mirror-symmetric homogeneous trimers composed of three 100 nm Au NSs.

A

Polarization	C_f (aF)	r_A (nm)	r_B (nm)	r_C (nm)	$R_{rad,A}$ (ohms)	$R_{rad,B}$ (ohms)	$R_{rad,C}$ (ohms)	$l_{m,AB}$ (fH)	$l_{m,BC}$ (fH)	$l_{m,AC}$ (fH)	C_{AB} (aF)	C_{BC} (aF)	C_{AC} (aF)
x-polarized	3.91	43.84	42.37	20	1	1	1	0	0.5	0	23	14	14
y-polarized	7.82	43.84	42.37	20	2	2	1	2.5	0.5	0.5	120	22	22

B

Polarization	C_f (aF)	r_A (nm)	r_B (nm)	r_C (nm)	$R_{rad,A}$ (ohms)	$R_{rad,B}$ (ohms)	$R_{rad,C}$ (ohms)	$l_{m,AB}$ (fH)	$l_{m,BC}$ (fH)	$l_{m,AC}$ (fH)	C_{AB} (aF)	C_{BC} (aF)	C_{AC} (aF)
x-polarized	1.56	19.07	19.07	42.37	1	1	3	0	1	1	20	35	35
y-polarized	2.52	19.07	19.07	42.37	1	1	4	0	1.5	1.5	13	32	32

C

Polarization	C_f (aF)	r_A (nm)	r_B (nm)	r_C (nm)	$R_{rad,A}$ (ohms)	$R_{rad,B}$ (ohms)	$R_{rad,C}$ (ohms)	$l_{m,AB}$ (fH)	$l_{m,BC}$ (fH)	$l_{m,AC}$ (fH)	C_{AB} (aF)	C_{BC} (aF)	C_{AC} (aF)
x-polarized	7.82	44.88	44.88	44.88	2	2	4	0	0.5	0.5	22	36	36
y-polarized	8.08	44.88	44.88	44.88	2.5	2.5	2	0.5	0.5	0.5	36	47	47

Table S5 | Nanocircuit parameters used for mirror-symmetric heterogeneous tetramers composed of two 100 nm and two 40 nm Au NSs.

x-polarized	C_f (aF)	r_A (nm)	r_B (nm)	r_C (nm)	r_D (nm)	$R_{rad,A}$ (ohms)	$R_{rad,B}$ (ohms)	$R_{rad,C}$ (ohms)	$R_{rad,D}$ (ohms)	$l_{m,AB}$ (fH)	$l_{m,BC}$ (fH)	$l_{m,AC}$ (fH)	$l_{m,AD}$ (fH)
	3.04	22.5	22.5	55	55	2.5	2.5	3	3	0	0.3	0.3	0.3
	$l_{m,BD}$ (fH)	C_{AB} (aF)	C_{AC} (aF)	C_{BC} (aF)	C_{AD} (aF)	C_{BD} (aF)							
	0.3	55	80	80	80	80							

y-polarized	C_f (aF)	r_A (nm)	r_B (nm)	r_C (nm)	r_D (nm)	$R_{rad,A}$ (ohms)	$R_{rad,B}$ (ohms)	$R_{rad,C}$ (ohms)	$R_{rad,D}$ (ohms)	$l_{m,AB}$ (fH)	$l_{m,BC}$ (fH)	$l_{m,AC}$ (fH)	$l_{m,AD}$ (fH)
	9.56	55	55	22.5	22.5	5	5	2	2	0	1.5	1.5	1.5
	$l_{m,BD}$ (fH)	C_{AB} (aF)	C_{AC} (aF)	C_{BC} (aF)	C_{AD} (aF)	C_{BD} (aF)							
	1.5	16	16	16	16	16							

Table S6 | Nanocircuit parameters used for PRET analysis. (A) YOYO-3-loaded 100 nm Au NS monomer. **(B)** Symmetric dimers composed of two 100 nm Au NSs.

A

Polarization	C_f (aF)	r_A (nm)	r_C (nm)	$R_{rad,A}$ (ohms)				
x-polarized	4.18	47.5	90	3.5				

B

Polarization	C_f (aF)	r_A (nm)	r_B (nm)	r_C (nm)	$R_{rad,A}$ (ohms)	$R_{rad,B}$ (ohms)	l_m (fH)	C_{AB} (aF)
x-polarized	10.83	47.5	47.5	15	14	14	13.5	2.25

7. Numerical FEM analysis

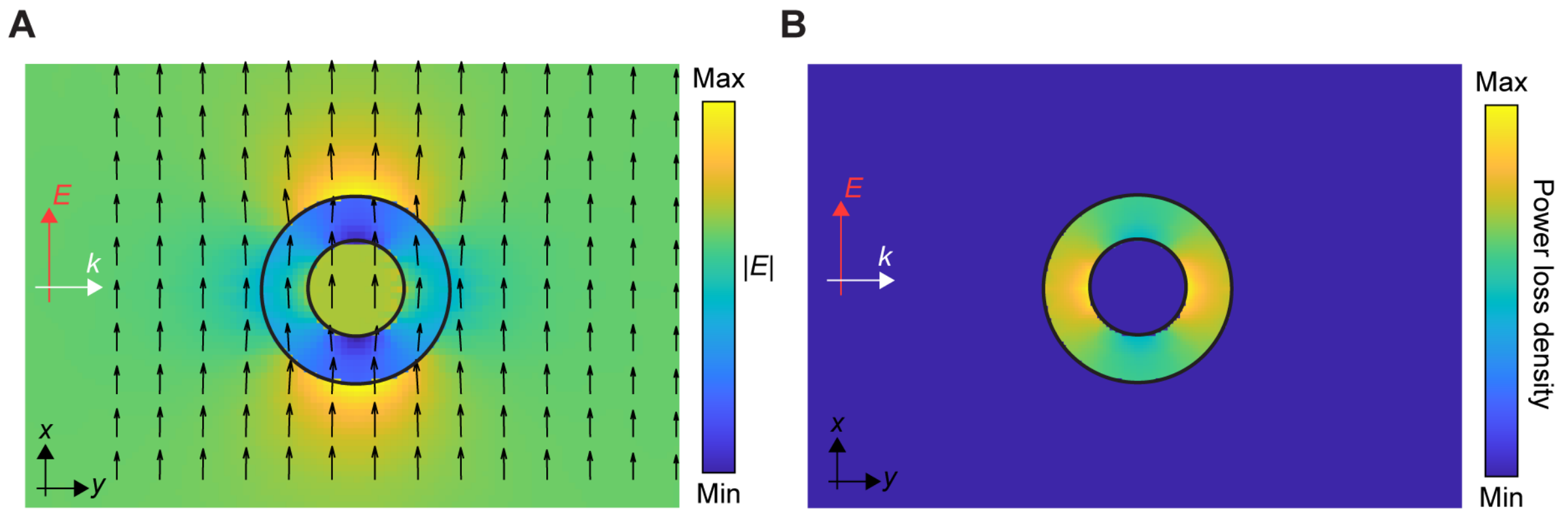

Fig. S4 | Full-wave numerical calculation of dye-loaded DNA origami. (**A**) Electric field distribution of dye-loaded DNA origami at the wavelength of 610 nm. (**B**) Power loss density distribution, revealing regions of optical absorption, of dye-loaded DNA origami at the wavelength of 610 nm.

The scattering and PRET behavior of the Au NS clusters was analyzed by FEM using CST Microwave Studio (2022). Three distinct geometric quantities are used throughout this work: (i) the nominal design gap derived from the programmed barrel/handle geometry; (ii) the population-level projected dry-state gap measured across 100 stoichiometrically target-positive structures; and (iii) the device-specific FEM input gap measured from the same individual nanocircuit used for the corresponding DF spectrum. The FEM curves paired with individual experimental spectra use the third quantity, including the particle diameters and all interparticle gaps of that device; they do not use the schematic label or the population average. The design derivations and device-specific FEM inputs are documented in Sections 26 and 27, respectively. The dielectric function of Au was taken from Johnson and Christy (63). The clusters were illuminated by a plane wave at 53° incidence with polarization varied from 0° to 90°, and scattering cross section was calculated from a far-field monitor.

The dielectric environments for the optical simulations were defined using effective medium approximations following established electrodynamic modeling for plasmonic nanostructures at dielectric interfaces (67). For dry-state DF scattering measurements, nanocircuits reside on a Formvar-coated TEM grid ($n_{\text{Formvar}} = 1.50$, $\varepsilon_{\text{Formvar}} = 2.25$) in air ($\varepsilon_{\text{air}} = 1.00$). The refractive index of the Formvar support film was taken from literature values (68). For both dry-state and in situ calculations, the effective refractive index was approximated as the arithmetic mean of the refractive indices of the two adjacent media, and the corresponding effective permittivity was calculated as $\varepsilon_{\text{eff}} = n_{\text{eff}}^2$. Thus, the Formvar/air environment gives neff = 1.25 and ε_{eff} = 1.56. For the aqueous-buffer/glass environment, $n_{\text{eff}} = (1.333 + 1.50)/2 = 1.4165$ and $\varepsilon_{\text{eff}} \approx 2.01$; the rounded value $\varepsilon_{\text{eff}} = 2.00$ was used in the PRET simulations.

The dielectric properties of the DNA components were also specifically defined. The permittivity of the barrel-shaped DNA origami was determined to be 1.45 via the Clausius-Mossotti relation (*35*):

$$\varepsilon_{\mathrm{DNA}} = \frac{3 + 2N\alpha_{\mathrm{e}}}{3 - N\alpha_{\mathrm{e}}}\varepsilon_{\mathrm{h}} \quad \text{(S7)}$$

Here, ε_{h}, $\varepsilon_{\mathrm{DNA}}$, N, and α_{e} are the relative permittivity of the host medium, the relative permittivity of the DNA origami, the number density of nucleotides, and the electric polarizability of nucleotides, respectively. The ssDNA shell on the Au NSs was modeled with a thickness of 7.53 nm and a permittivity of 1.93, based on its optically quantified ssDNA loading density (*35*). The comprehensive sensitivity of the optical resonances to variations in local dielectric parameters is demonstrated in Section 32.

For the PRET analysis specifically, the optical response of the dye molecules was included by modeling the dielectric function of the dye-loaded dsDNA shell and DNA origami with a Lorentzian oscillator model (*69*):

$$\varepsilon = \varepsilon_{\infty} + \frac{f_1\omega_{0,1}^2}{\omega_{0,1}^2 - \omega^2 - i\gamma_{a,1}\omega} + \frac{f_2\omega_{0,2}^2}{\omega_{0,2}^2 - \omega^2 - i\gamma_{a,2}\omega} \quad \text{(S8)}$$

In this equation, ε_{∞} is the background dielectric constant, ω_0 is the resonant angular frequency, and γ_{a} is the linewidth of the dye molecule. The key parameter, f, is a dimensionless oscillator strength defined as:

$$f_i = \frac{N}{V}\mu^2\frac{2}{3\varepsilon_0\hbar\omega_{0,i}} \quad \text{(S9)}$$

where N is the number of dye molecules, V is the volume of the DNA shell or origami, μ is the dipole moment of the dye, ε_0 is the vacuum permittivity, and $\hbar$ is the reduced Planck constant. The parameters for the YOYO-3 dye were determined from experimental measurements, using a two-oscillator model to capture its absorption profile: $\omega_{0,1}$ = 2.16 eV, $\omega_{0,2}$ = 2.03 eV; $\gamma_{\mathrm{a},1}$ = 0.20 eV, $\gamma_{\mathrm{a},2}$ = 0.12 eV; and μ = 8.5 D for both oscillators.

8. Synthesis of 40–100 nm Au NSs

Au NSs with diameters ranging from 40 to 100 nm were synthesized following a modified, multi-step method of iterative reductive growth and oxidative dissolution (*33*, *34, 35*). All chemical reagents were purchased from Sigma-Aldrich and used as received. The synthesis began with the preparation of an Au seed solution by injecting 125 μL of Gold (III) chloride trihydrate ($HAuCl_4$, 10 mM) into 5 mL of a hexadecyltrimethylammonium bromide (CTAB, 100 mM) solution under vigorous stirring. After 30 seconds, 300 μL of ice-cold sodium borohydride ($NaBH_4$, 10 mM) was rapidly injected. The solution was stirred for 1 minute and then incubated at 28°C for 30 minutes to decompose any residual $NaBH_4$.

This seed solution was then used to grow single-crystal Au nanorods (Au NRs). In a 200 mL solution of CTAB (100 mM), 10 mL of $HAuCl_4$ (10 mM) was injected, followed by the sequential addition of silver nitrate ($AgNO_3$, 1.8 mL, 10 mM), L-ascorbic acid (1.14 mL, 100 mM), and 240 μL of the seed solution, with 30-second intervals of mild stirring between each addition. After a minute of stirring, the solution was incubated at 28°C for 2 hours. Following growth, the ends of the Au NRs were etched out. To optimize this step, small batches of Au NRs (2 OD) in CTAB (50 mM) were incubated with varying concentrations of $HAuCl_4$ (from 50 to 100 μM) at 40°C for 2 hours. From these optimization experiments, the ideal $HAuCl_4$ concentration was typically found to be in the 70–80 μM range. This procedure was then repeated on a large scale using an optimized etchant concentration to produce the etched Au NRs (eAu NRs).

These eAu NRs were used as seeds to grow concave rhombic dodecahedra (CRDs). In 80 mL of a cetylpyridinium chloride (CPC, 20 mM) solution, 1.4 mL of $HAuCl_4$ (10 mM) and 18 mL of L-ascorbic acid (100 mM) were injected. Subsequently, 3 mL of the eAu NR seeds (1 OD), dispersed in CPC (10 mM), was added to the reaction solution and stirred for 15 minutes. To produce the 40 nm Au NSs, these CRDs (1 OD) were dispersed in CTAB (50 mM), mixed with $HAuCl_4$ to a final concentration of 60 μM, and incubated at 40°C for 2 hours.

The larger 100 nm Au NSs were prepared by using the 40 nm Au NSs as seeds in another growth and dissolution cycle. First, larger CRDs were grown by adding 4 mL of the 40 nm Au NSs (1 OD), dispersed in CPC (10 mM), to a solution containing CPC (80 mL, 20 mM), $HAuCl_4$ (1.4 mL, 10 mM), and L-ascorbic acid (18 mL, 100 mM), and stirring for 15 minutes. These larger CRDs (1 OD), dispersed in CTAB (50 mM), were then etched with $HAuCl_4$ (60 μM) at 40°C for 2 hours to produce the 100 nm Au NSs.

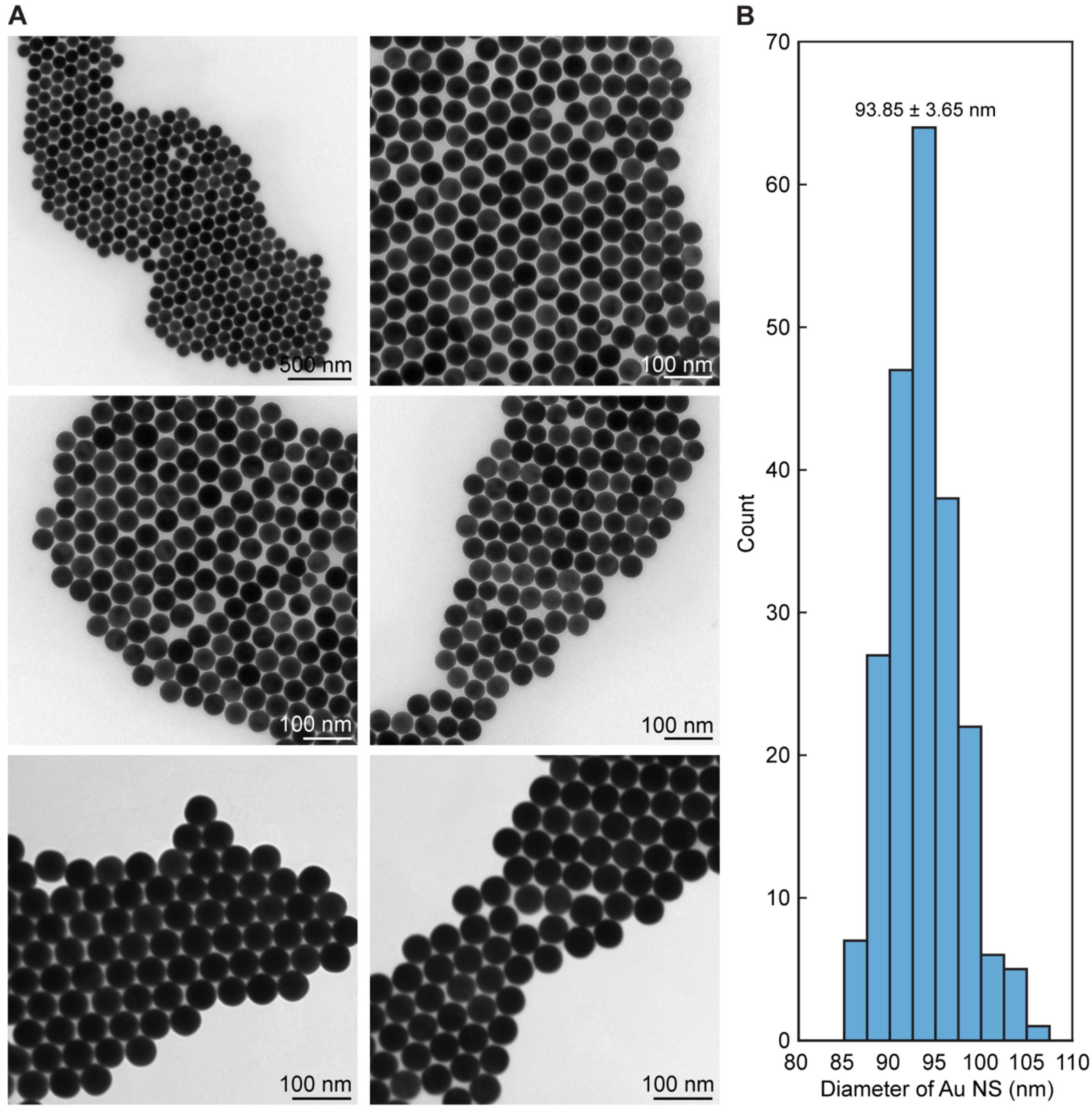

Fig. S5 | Characterization of the synthesized 100 nm Au NSs. (**A**) Representative TEM images showing the uniform, highly spherical, and ultrasmooth features of Au NSs. (**B**) The corresponding Au NS diameter distribution histogram, obtained from analysis of multiple TEM images.

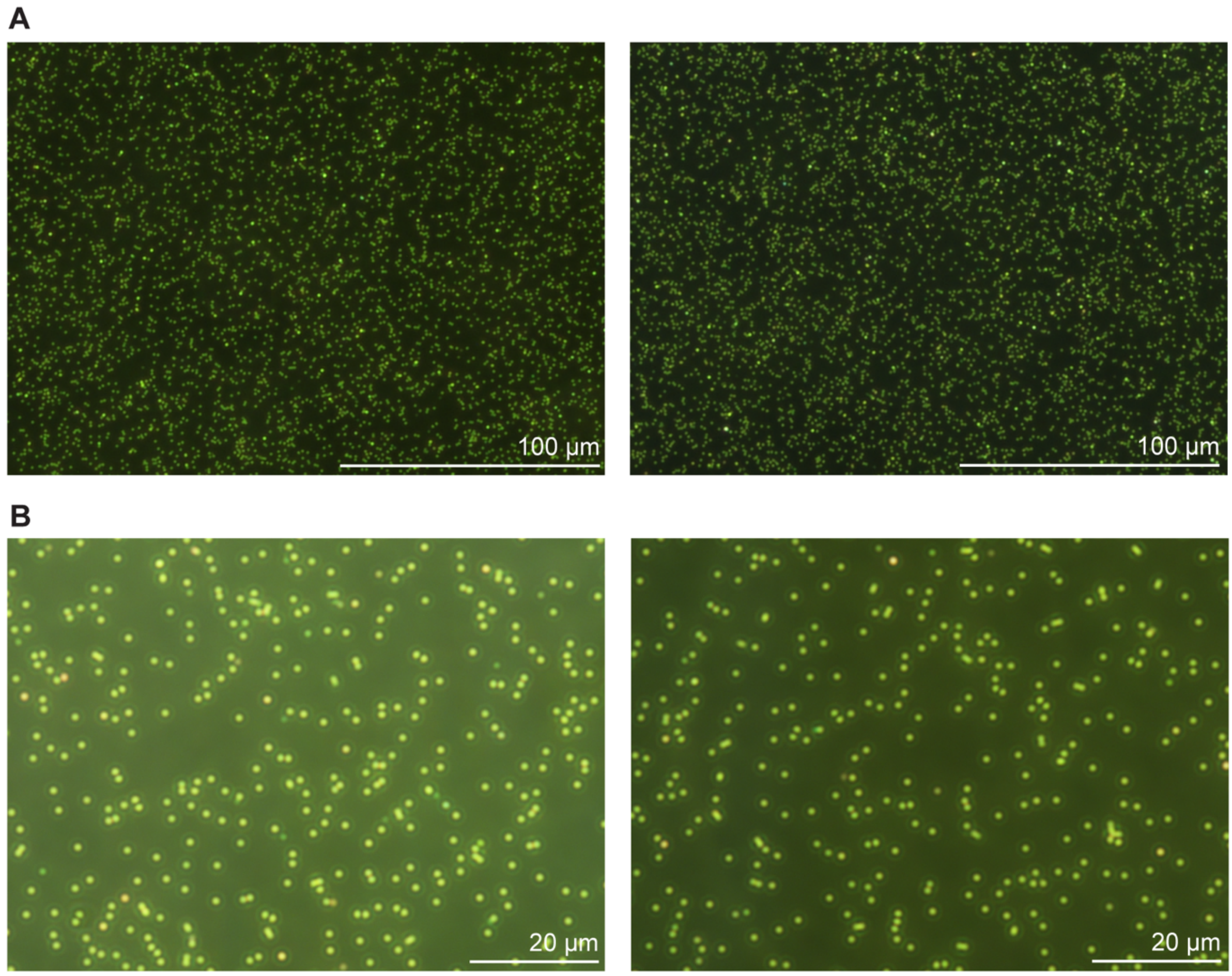

Fig. S6 | Dark-field optical microscope images of 100 nm Au NSs. (**A, B**) Representative dark-field optical microscope images of Au NSs immobilized on the surface of a glass microfluidic chamber, viewed with (**A**) 10x and (**B**) 50x objective lens. The consistent green color and brightness of the scattered light from individual NSs indicate the high size and shape uniformity of the synthesized Au NSs.

9. Absorption and scattering cross-sections (ACS and SCS) of 30–100 nm Au NSs

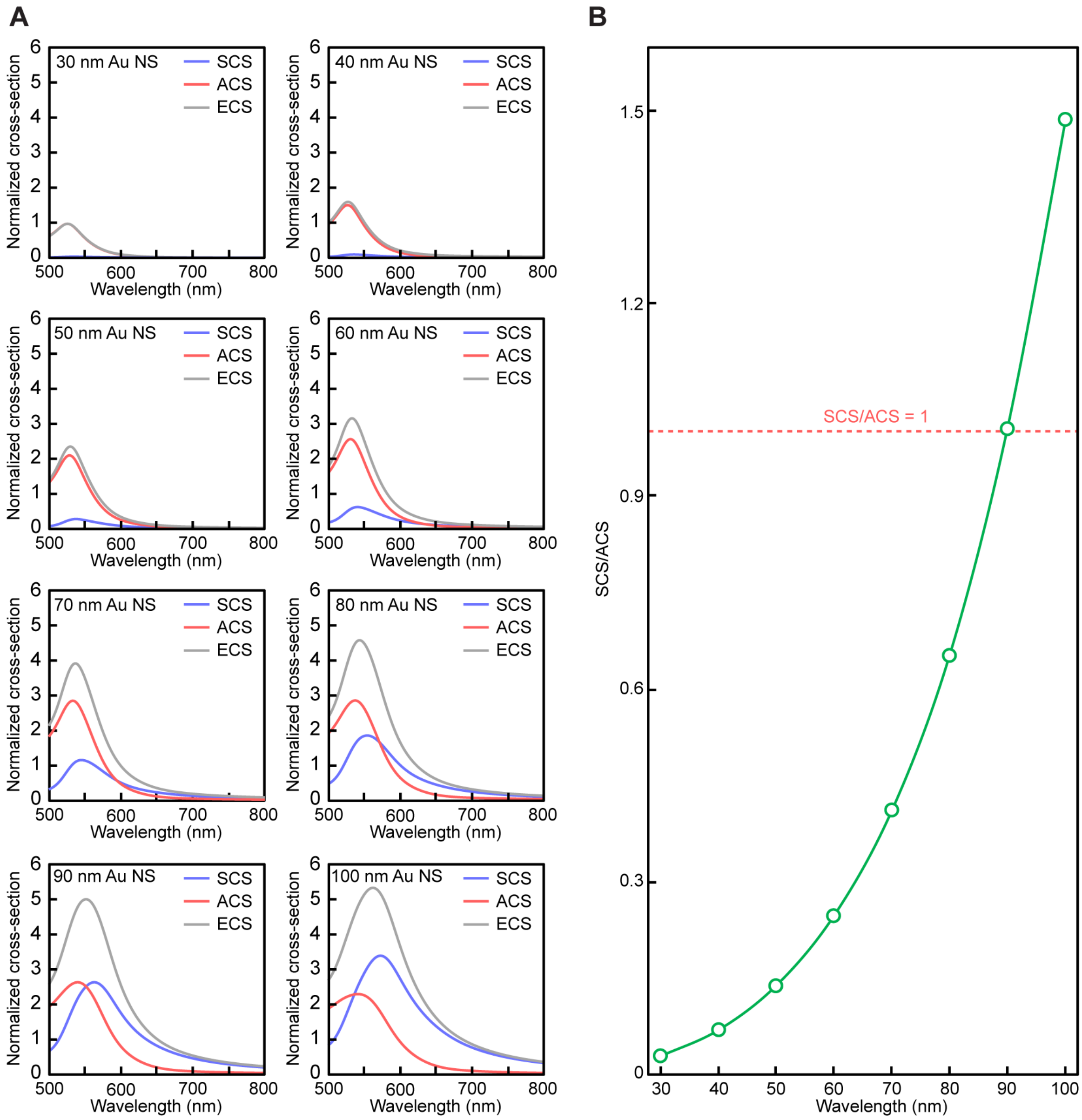

Fig. S7 | Calculated cross-section of Au NSs as a function of diameter. (**A**) Numerically calculated absorption (ACS), scattering (SCS), and extinction (ECS) cross-sections for Au NSs with varying diameters. (**B**) The corresponding ratio of SCS to ACS. The red dotted line highlights the crossover point where the scattering efficiency begins to dominate over absorption efficiency.

The absorption cross-section (ACS) and scattering cross-section (SCS) for Au NSs with diameters ranging from 30 to 100 nm were calculated using the FEM, as detailed in Section 7 of the Supplementary Information. To determine the ACS, the power loss density was integrated within the volume of the Au NS and then normalized by the intensity of the incident plane wave. For the SCS, the scattered electromagnetic field was first determined by subtracting the incident field from the total calculated field. The SCS was then found by

integrating the Poynting vector of this scattered field over a closed surface enclosing the nanosphere and normalizing the value by the incident plane wave intensity.

The calculated ACS, SCS, and their ratio (SCS/ACS) for different Au NS diameters are shown in **Figure S7**. For larger Au NSs (diameters exceeding 90 nm), the SCS becomes substantially greater than the ACS. This indicates that larger nanoparticles are more efficient at scattering energy than absorbing it. Therefore, plasmonic resonators assembled from these larger Au NSs can more effectively support complex, higher-order resonance modes (e.g., magnetic dipolar and Fano resonances) that rely on strong capacitive coupling between the Au NSs.

10. DNA-PAINT analysis of barrel DNA origami

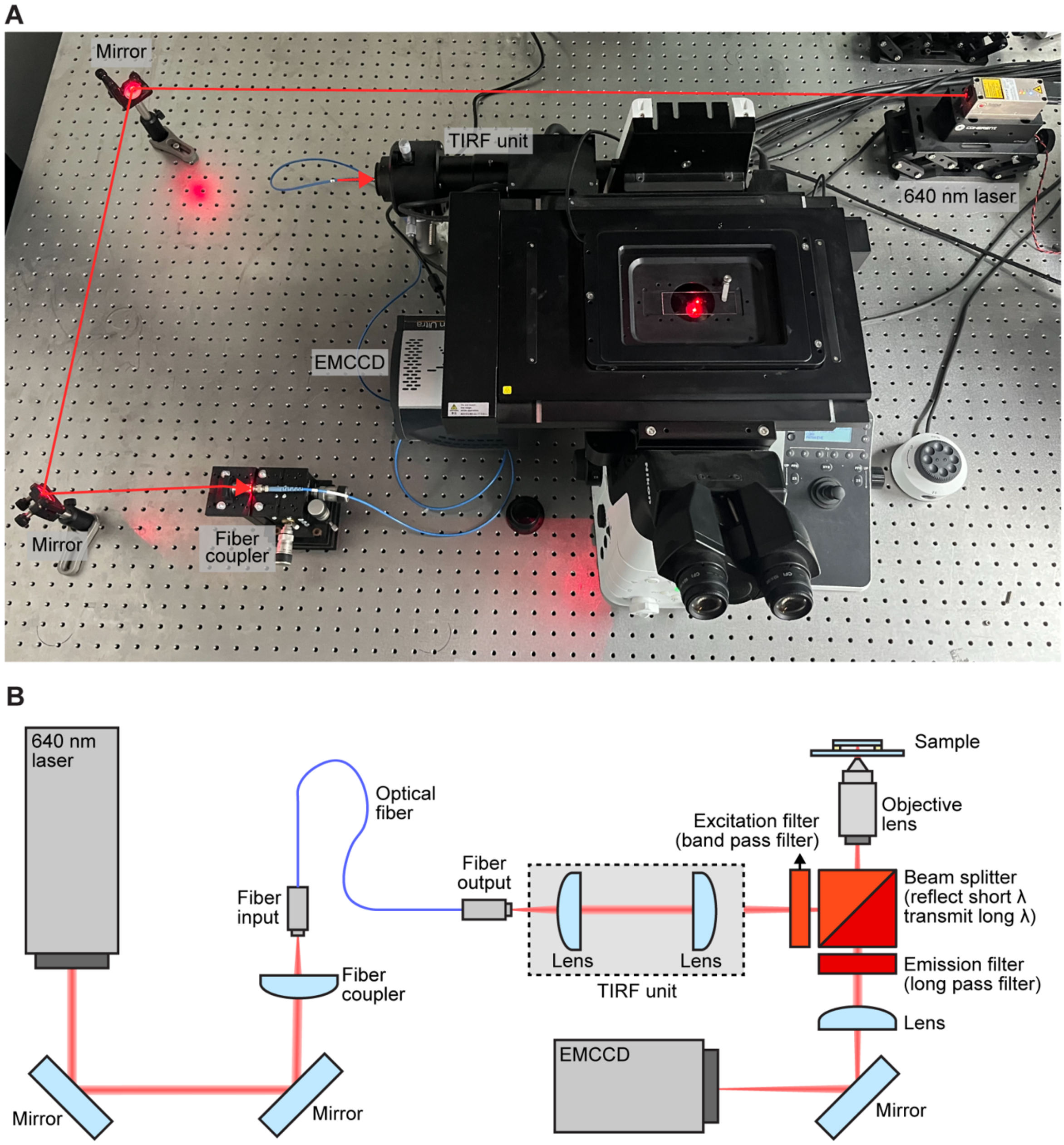

Fig. S8 | Custom-built PAINT microscopy setup. (**A**) Photograph of the experimental setup showing the layout of the instrument. (**B**) Corresponding schematic diagram illustrating the optical path and the arrangement of key components, such as the objective lens, filters, and detector.

DNA-PAINT imaging was performed on a custom-built microscopy setup, as illustrated in **Figure S8**. A 640 nm laser (TOPTICA, iBeam smart) was used as the excitation light source. The light was delivered through a polarization-maintaining single-mode optical fiber to ensure a high-quality Gaussian beam profile. The beam was directed through a TIRF illumination unit into a Nikon Ti2 series microscope. The light was then passed through a bandpass filter and beam splitter before being focused by a high numerical aperture objective lens (Nikon, Apo TIRF) to illuminate the sample. The resulting fluorescence emission was collected by the same

objective lens, passed through an emission filter to block scattered excitation light, and imaged onto an EMCCD camera (Andor, iXon Ultra 888).

For the DNA-PAINT analysis of barrel DNA origami, the samples were specifically designed by incorporating 24 short docking strands on their outer surface (as shown in **Figure 2A** of the main manuscript) and 6 biotin molecules on their bottom surface. These structures were immobilized within a fluidic chamber via a biotin-streptavidin linkage. Briefly, the chamber surface was coated with BSA-biotin and then treated with streptavidin, following a previously reported protocol (*64*). Then, the biotin-tagged DNA origami was introduced and incubated to allow for immobilization on the surface. For imaging, a solution containing 5 nM of fluorescently-labeled imager strands, which are complementary to the docking strands, was introduced into the chamber. A total of 10,000 image frames were acquired using an exposure time of 250 ms per frame and without EM amplification. The final super-resolution images were reconstructed from the resulting localization data using the Picasso software package (*64*).

11. High-magnification TEM images of dimeric Au NS assemblies

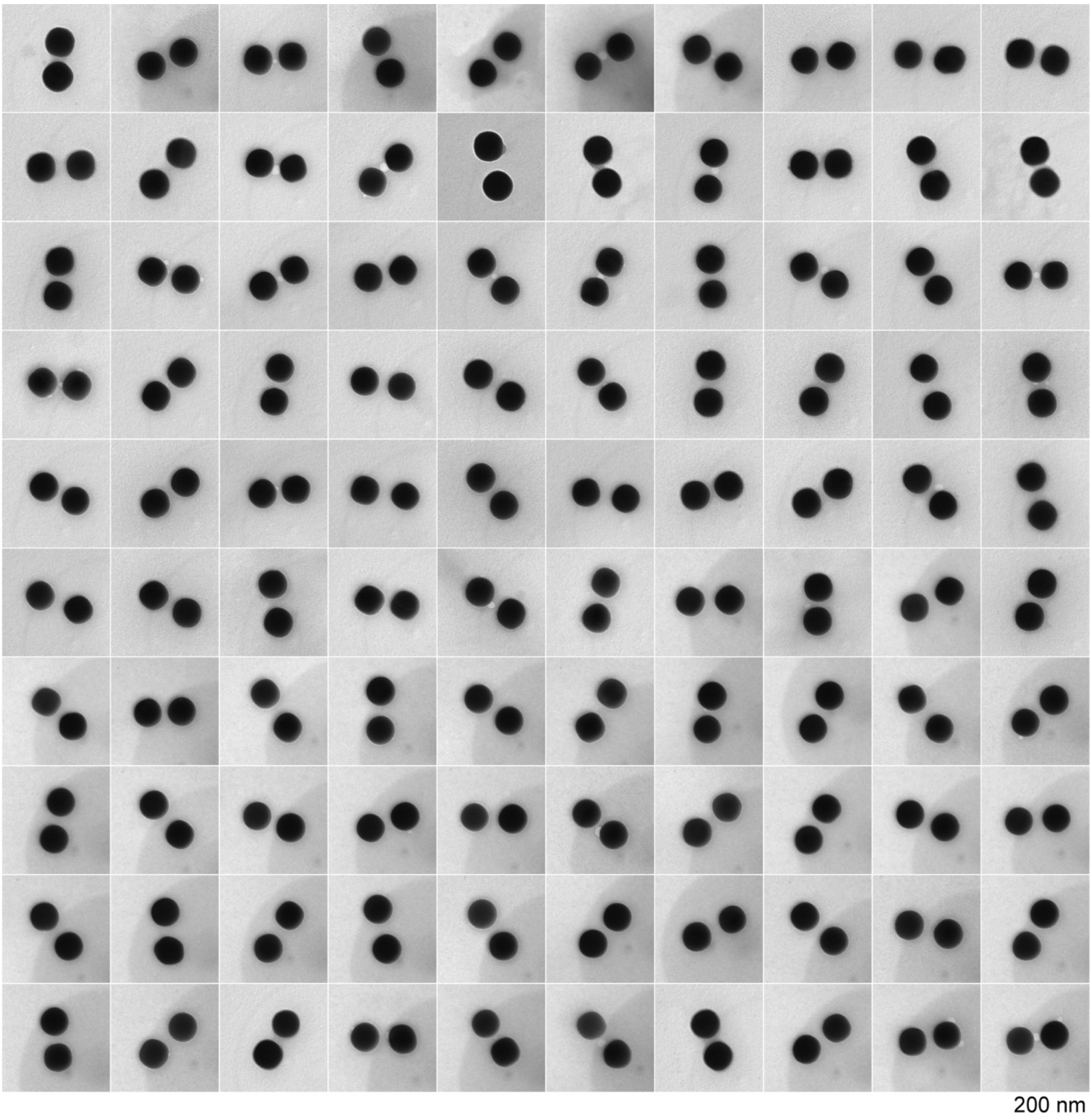

Fig. S9 | High-magnification TEM images of symmetric dimers consisting of two 100 nm Au NSs. These independent stoichiometrically target-positive images were translationally and rotationally aligned and used to generate the aligned average shown in **Figure 2F** of the main manuscript. Source-image and instrument-control analyses of the recurrent fixed-pattern background are provided in Section 31.

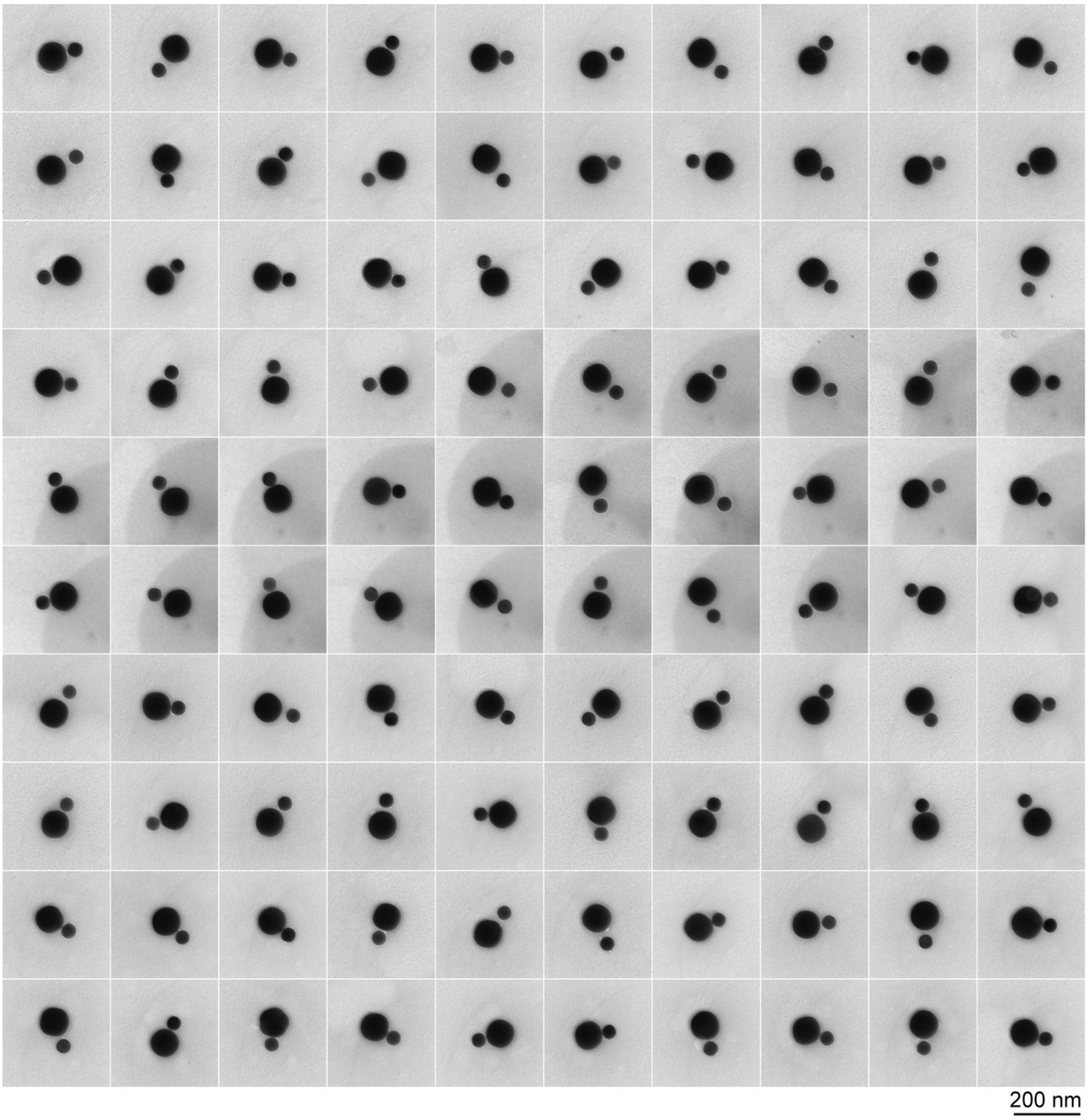

Fig. S10 | High-magnification TEM images of asymmetric dimers consisting of 40 nm and 100 nm Au NSs. These independent stoichiometrically target-positive images were translationally and rotationally aligned and used to generate the aligned average shown in **Figure 2G** of the main manuscript. Source-image and instrument-control analyses of the recurrent fixed-pattern background are provided in Section 31.

12. Analysis of polarization-resolved dark-field scattering spectra

12.1. Dark-field microspectroscopy setup

Scattering spectra were measured using a custom-built dark-field microspectroscopy system (*34*). Samples were illuminated with a broadband light source at an incident angle of 53°. The scattered light was collected through a 50x objective lens (NA 0.8) and directed to an imaging spectrometer (Princeton Instruments, IsoPlane) coupled with a CCD camera (Princeton Instruments, PIXIS-400B) for spectral analysis.

To investigate the resonant scattering behavior of the Au NS assemblies upon differently polarized light illumination, we performed polarization-resolved measurements. A linear polarizer was placed in the illumination path to set the polarization of the incident light. A second linear polarizer, acting as an analyzer, was placed in the detection path and aligned parallel to the first to selectively measure the co-polarized scattered light. Spectra were recorded by varying the incident polarization from 0° to 90°. For these measurements, 0° (horizontal) is defined as the orientation where the incident electric field is parallel to the long axis of the Au NS assembly, while 90° (vertical) corresponds to a perpendicular orientation.

12.2. Sample preparation and dark-field scattering measurement

To correlate the structure of Au NS clusters with their optical properties, assembled Au NS clusters were deposited onto a Formvar film supported by a 3-meshed TEM grid (*34*). This substrate was chosen because it enables TEM characterization before the scattering spectrum of a selected cluster is measured. Furthermore, the optically thin Formvar film provides a high signal-to-noise ratio by minimizing background scattering. Other common substrates (e.g., glass and silicon wafers) were considered unsuitable for this correlative analysis. While glass offers a low scattering background, it is unsuitable for characterizing the target clusters with an electron microscope before the optical measurement. Silicon wafers, though compatible with both techniques, have a high refractive index that can distort the plasmonic resonances through image-dipole interactions (*34*).

The scattering spectra measurement procedure began with a pre-screening step to ensure the reliability of the data acquisition. The TEM grid was first inspected using the dark-field optical microscope to select regions with low background noise, minimal contamination, and an intact Formvar film. Once a suitable region was identified, it was imaged using low-magnification (i.e., 1000×) TEM to create a map of well-isolated nanoclusters. Following this, intermediate-magnification (i.e., 3000–5000×) TEM was used to determine the precise orientation of individual target clusters. To avoid beam damage before optical measurement, high-magnification imaging was avoided. Subsequently, the same TEM grid was transferred to the dark-field microscopy stage. Using the low-magnification TEM map as a guide, each structurally-characterized cluster was relocated. Finally, the polarization-resolved scattering spectrum of the single, pre-characterized cluster was measured. A representative example of this correlative imaging process is shown in **Figure S11**. High-magnification structural images were acquired after the correlative optical measurement. Each target was centered at approximately the same detector coordinate before acquisition under the same magnification and detector settings. Consequently, an instrument-fixed feature can recur at the same relative coordinate in identically sized crops from independent acquisitions. Uncropped source images

and blank/no-grid controls are provided in Section 31. Processing of the displayed galleries was limited to cropping and, where necessary, whole-image rotation; construction of aligned averages additionally used linear x/y translation and rotation. No local intensity adjustment, local filtering, or background subtraction was applied. Every displayed crop was traced to a unique source acquisition.

For characterization of the PRET system, a microfluidic chamber was constructed from two sandwiched soda-lime glass slides. One inner glass surface was treated with oxygen plasma to create a hydrophilic surface, which facilitated the immobilization of the dye-loaded Au NS monomers and dimers. This microfluidic device allowed for in-situ control over the sample environment during the scattering measurement. Once the nanostructures were immobilized, the buffer solution in the chamber was exchanged with solutions containing various concentrations of YOYO-3 dye. This process enabled precise control over the dye-loading density on the Au NS monomers and dimers while the scattering spectra were being measured.

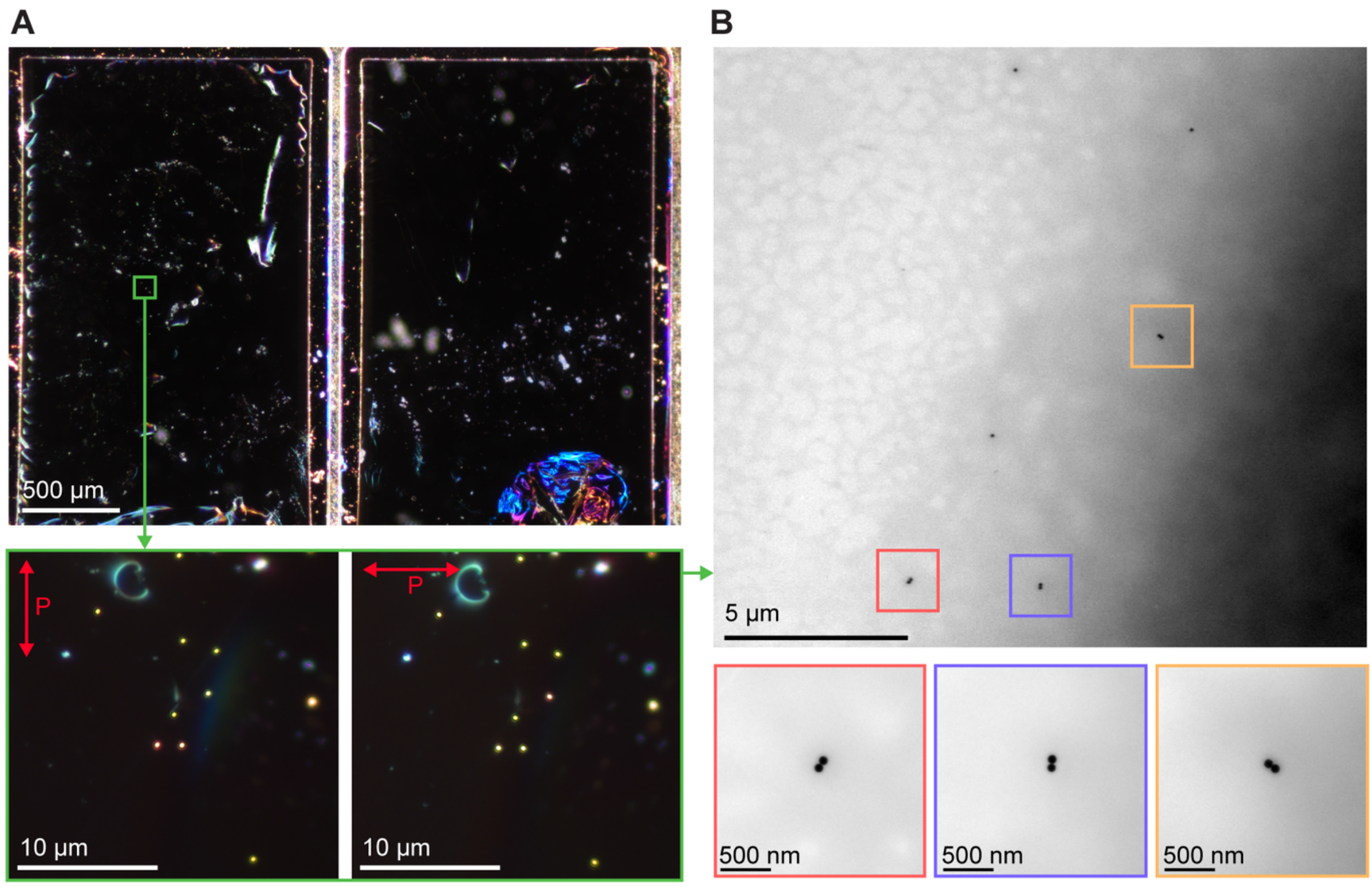

Fig. S11 | Representative example of correlative imaging for dark-field scattering measurement. (**A**) Representative dark-field optical microscope images of Au NS dimers on a Formvar film supported by a 3-meshed TEM grid. The arrow labeled 'P' indicates the polarization of the incident light. (**B**) Corresponding TEM images of the exact same region. The top panel shows a low-magnification TEM map. The bottom panel provides an intermediate-magnification view, which is used to determine the precise orientation of the target dimer before spectral measurement.

13. Correlative TEM images and individual dark-field spectra of dimeric Au NS assemblies

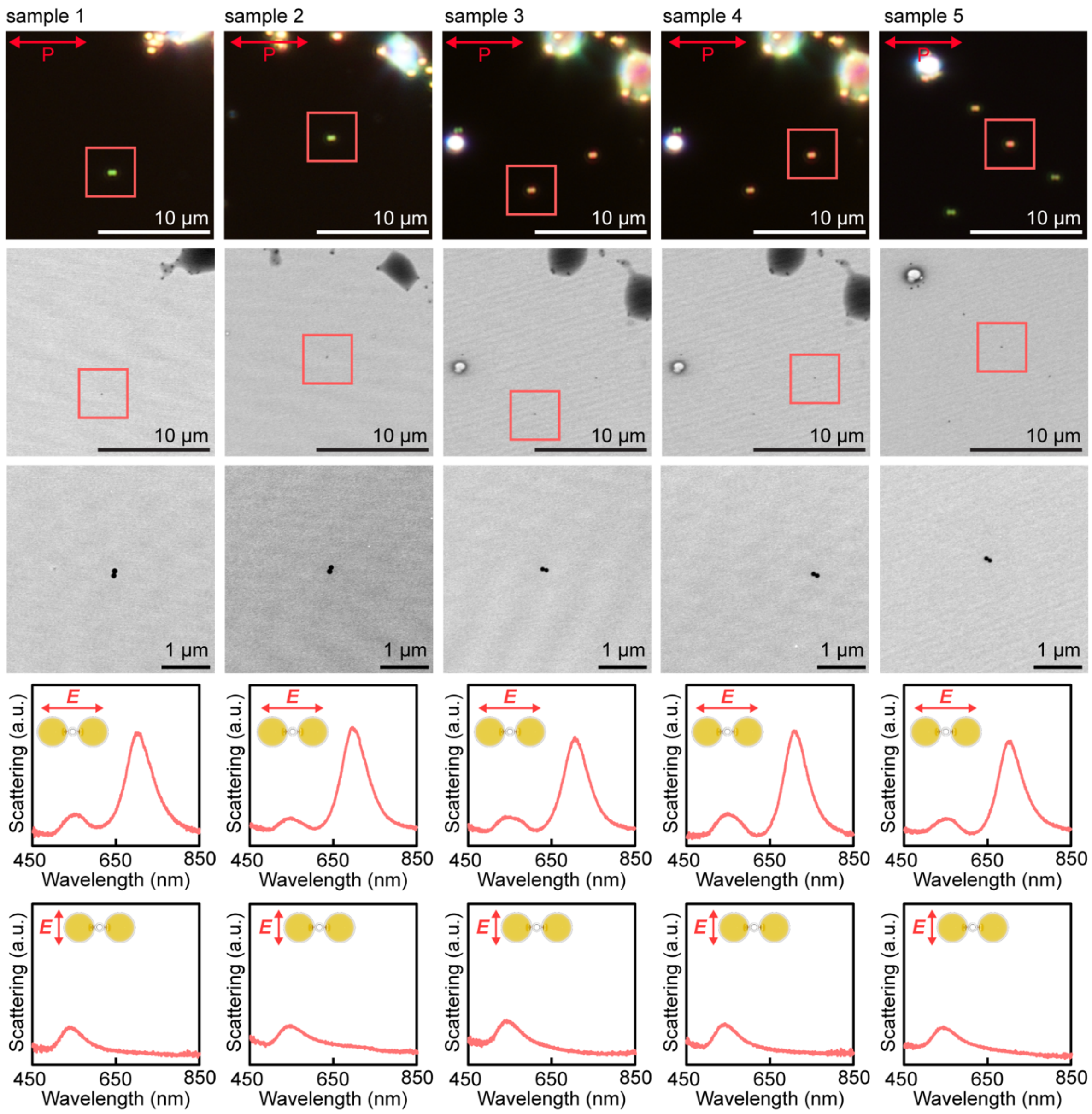

Fig. S12 | Correlative TEM images and dark-field scattering spectra of symmetric dimers consisting of two 100 nm Au NSs. One-to-one correlative analysis of five displayed, structurally identified target nanocircuits in Figure 2H (Samples 1–5, marked by red boxes). From top to bottom, the first three rows represent: dark field optical micrographs of individual nanocircuits with incident polarization indicated by red arrows (P), low-magnification TEM maps used for target localization, and intermediate-magnification TEM images used to determine the precise orientation of each nanocircuit. The bottom two rows show the corresponding polarization-resolved dark-field scattering spectra obtained under parallel (fourth row) and perpendicular (fifth row) excitation, respectively. The five displayed devices are the denominator for this figure; the targeted correlative workflow was not designed as an unbiased optical-yield survey.

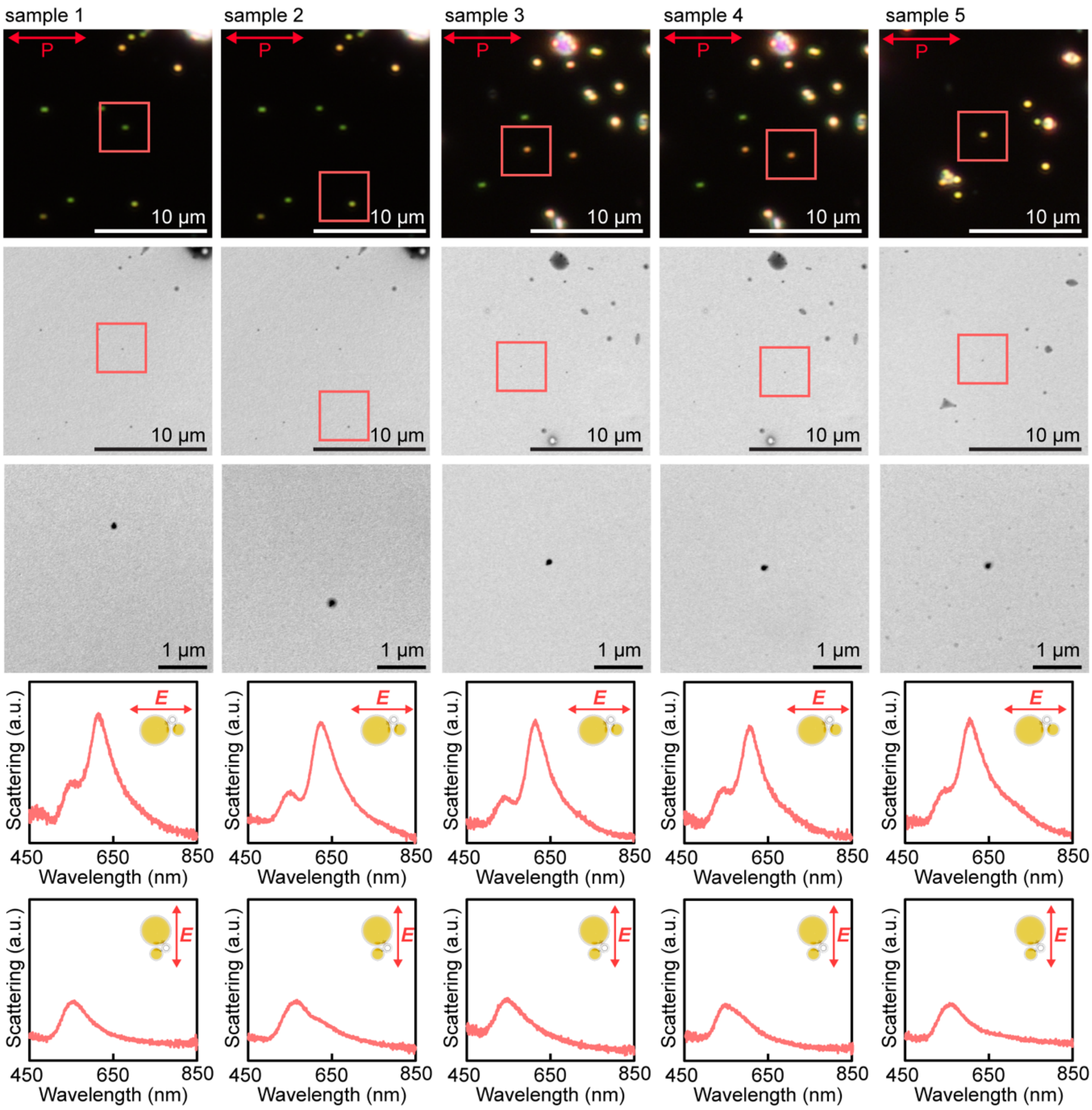

Fig. S13 | Correlative TEM images and dark-field scattering spectra of asymmetric dimers consisting of 40 nm and 100 nm Au NSs. One-to-one correlative analysis of five displayed, structurally identified target nanocircuits in Figure 2I (Samples 1–5, marked by red boxes). From top to bottom, the first three rows represent: dark field optical micrographs of individual nanocircuits with incident polarization indicated by **P**, low-magnification TEM maps used for target localization, and intermediate-magnification TEM images used to determine the precise orientation of each nanocircuit. The bottom two rows show the corresponding polarization-resolved dark-field scattering spectra obtained under parallel (fourth row) and perpendicular (fifth row) excitation, respectively. The five displayed devices are the denominator for this figure; the targeted correlative workflow was not designed as an unbiased optical-yield survey.

14. Preparation of DNA-functionalized Au NSs

To functionalize the Au NSs with DNA, we utilized the stable pseudo-covalent bond formed between gold and a thiol group tagged at the end of a DNA oligo. The as-synthesized Au NSs, initially dispersed in CTAB (10 mM), were first washed to remove residual surfactant by concentrating and redispersing them in deionized (DI) water. The washed Au NSs were then dispersed in a buffer solution containing 0.05 wt% sodium dodecyl sulfate (SDS) and 10 mM Tris-HCl, along with the thiolated anti-handle ssDNA. This anti-handle DNA is complementary to the handle strands decorated on the surface of the barrel DNA origami. The concentration of the anti-handle ssDNA was maintained above 3 μM throughout the procedure. After a 1-hour incubation in a mixer, a salt-aging technique was employed to maximize the DNA loading density on the Au NS surface (*35*, *61*, *62*). This technique reduces the electrostatic repulsion between DNA strands. A high-salt buffer (2 M NaBr, 0.05 wt% SDS, 10 mM Tris-HCl) was gradually injected into the solution over 2 days until the final NaBr concentration reached 750 mM.

Successful DNA conjugation was confirmed by observing a 1-2 nm redshift in the extinction spectra of the DNA-conjugated Au NSs compared to the bare particles. The successful functionalization was further supported by a salt screening assay, which assesses the colloidal stability of the NSs in high-salt solutions. Finally, before their use in cluster assembly, the DNA-conjugated Au NSs were washed by cetnrifugating and redispersing them in buffer (0.05 wt% SDS, 10 mM Tris-HCl) to remove any excess anti-handle ssDNA. The thiolated anti-handle ssDNA was purchased from Bioneer, and all other chemical reagents were ordered from Sigma-Aldrich.

15. *Q*-factor analysis of electric resonance modes

The *Q*-factor, or quality factor, defined as the ratio of power stored to power dissipated in a resonance circuit, reflects the spectral sharpness of resonance–higher *Q* factors correspond to narrower bandwidths and greater spectral selectivity.

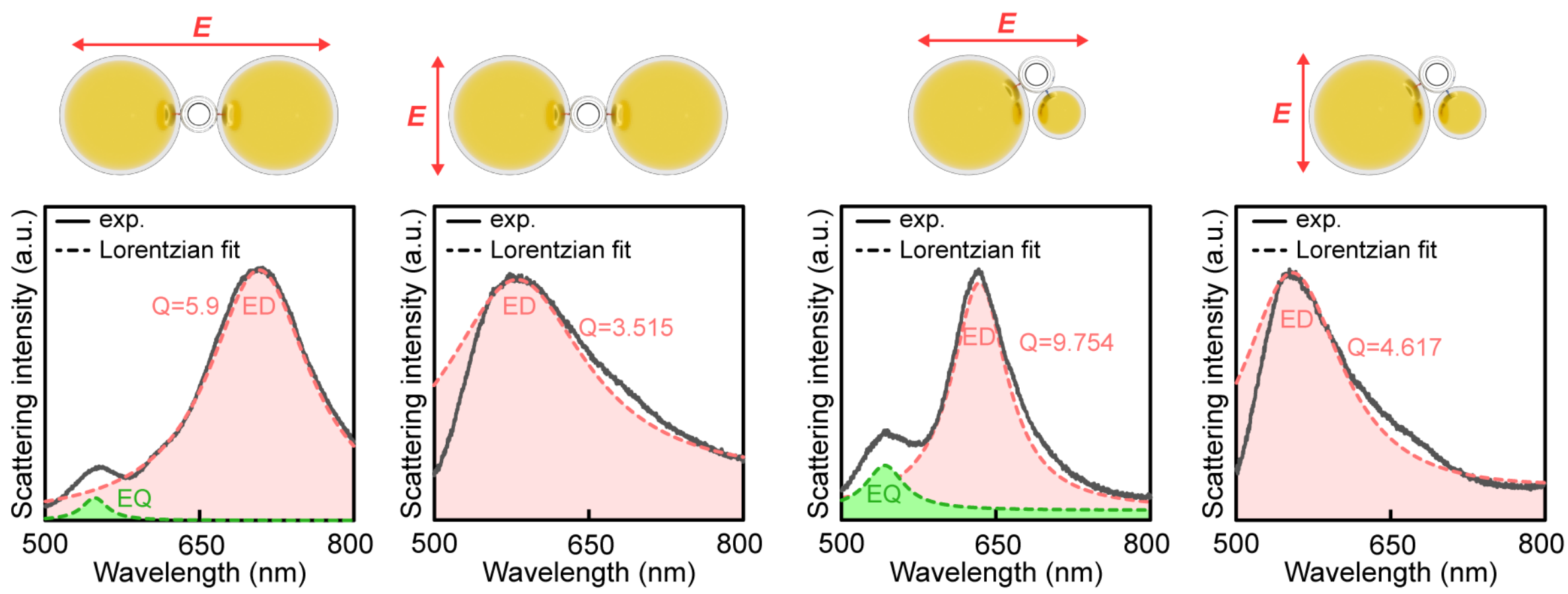

Fig. S14 | *Q*-factor analysis of the electric dipole (ED) resonance of Au NS dimers. (Top panel) Schematic illustration of the Au NS dimer under different incident polarizations. (Bottom panel) Representative measured scattering spectrum (solid line) from an assembled dimer. The dashed line shows a Lorentzian fit applied to the resonance mode and used to extract its *Q*-factor.

16. Electric quadrupolar mode of Au NS dimers

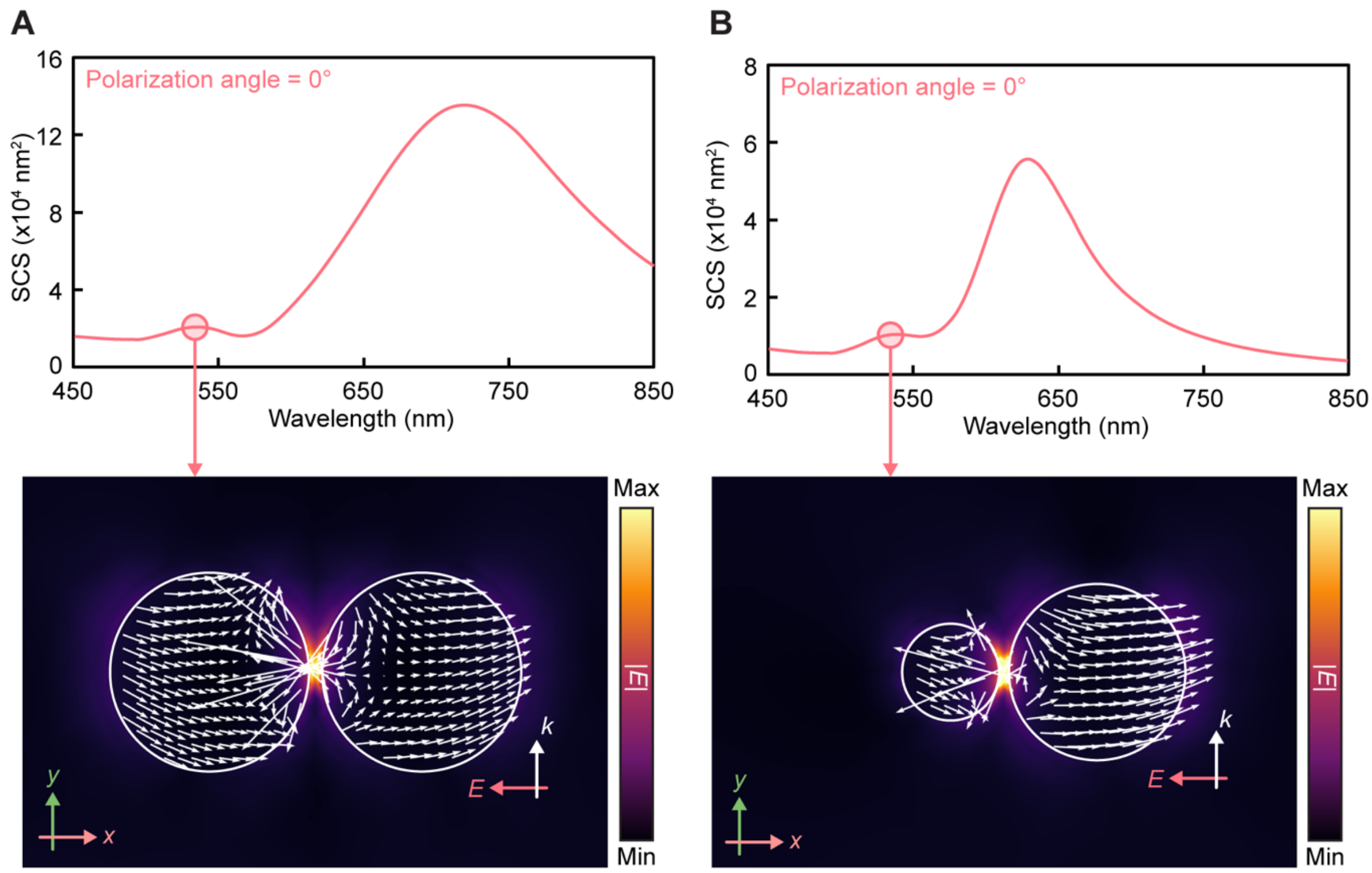

Fig. S15 | Electric field distribution of the electric quadrupolar (EQ) mode in Au NS dimers. (**A, B**) (Top panel) numerically simulated SCS for (**A**) a symmetric and (**B**) an asymmetric Au NS dimer. (Bottom panel) Corresponding electric field distributions calculated at each dimer's respective EQ resonance wavelength. The white arrows indicate the internal electric field vectors within the nanoparticles. This complex, non-dipolar field pattern at the EQ mode cannot be captured by the fundamental dipolar approximation used in the lumped optical nanocircuit model.

17. High-magnification TEM images of trimeric and tetrameric Au NS assemblies

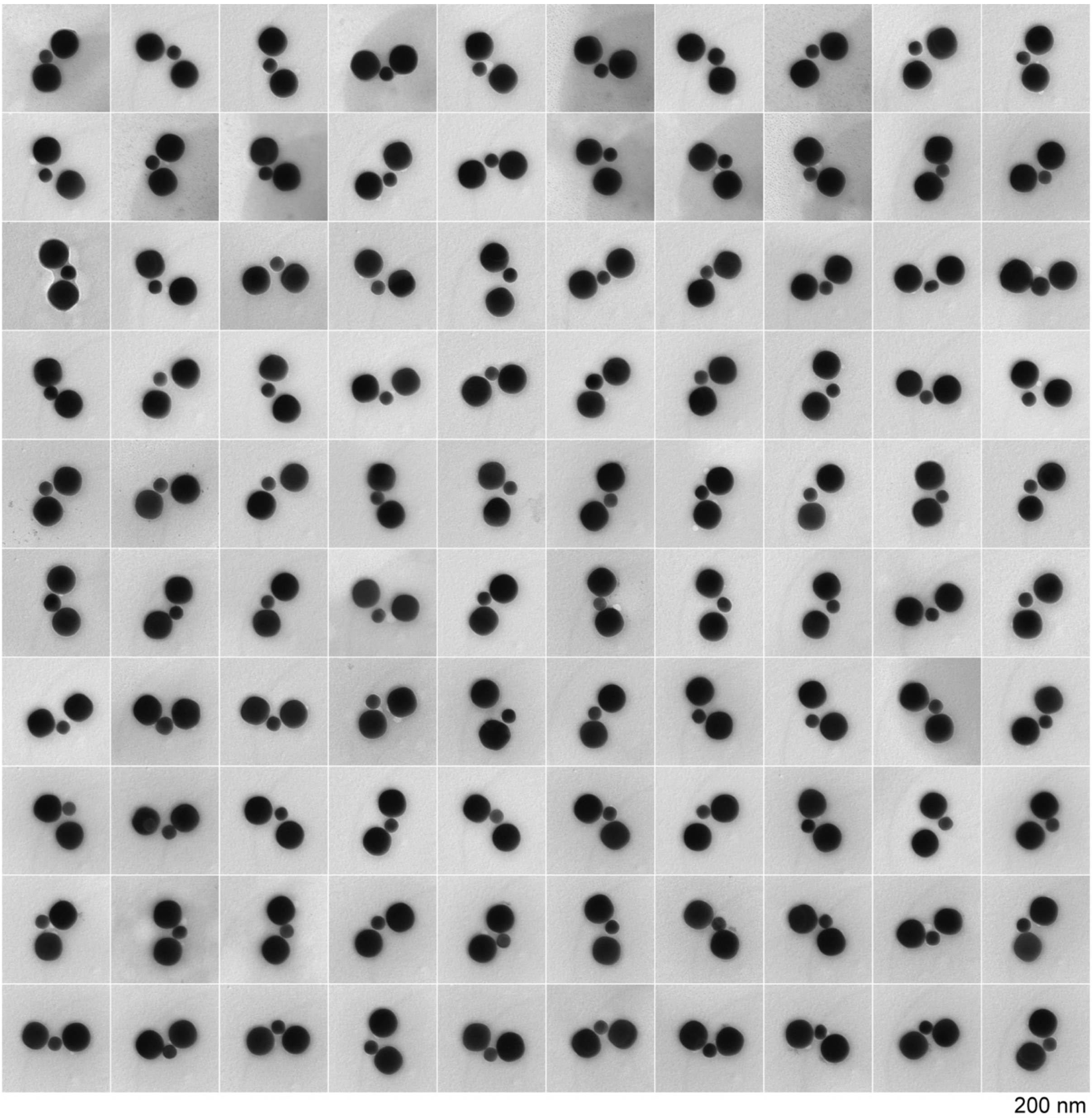

Fig. S16 | High-magnification TEM images of trimers consisting of two 100 nm Au NSs and one 40 nm Au NS. These independent stoichiometrically target-positive images were translationally and rotationally aligned and used to generate the aligned average shown in **Figure 3A** of the main manuscript. Source-image and instrument-control analyses of the recurrent fixed-pattern background are provided in Section 31.

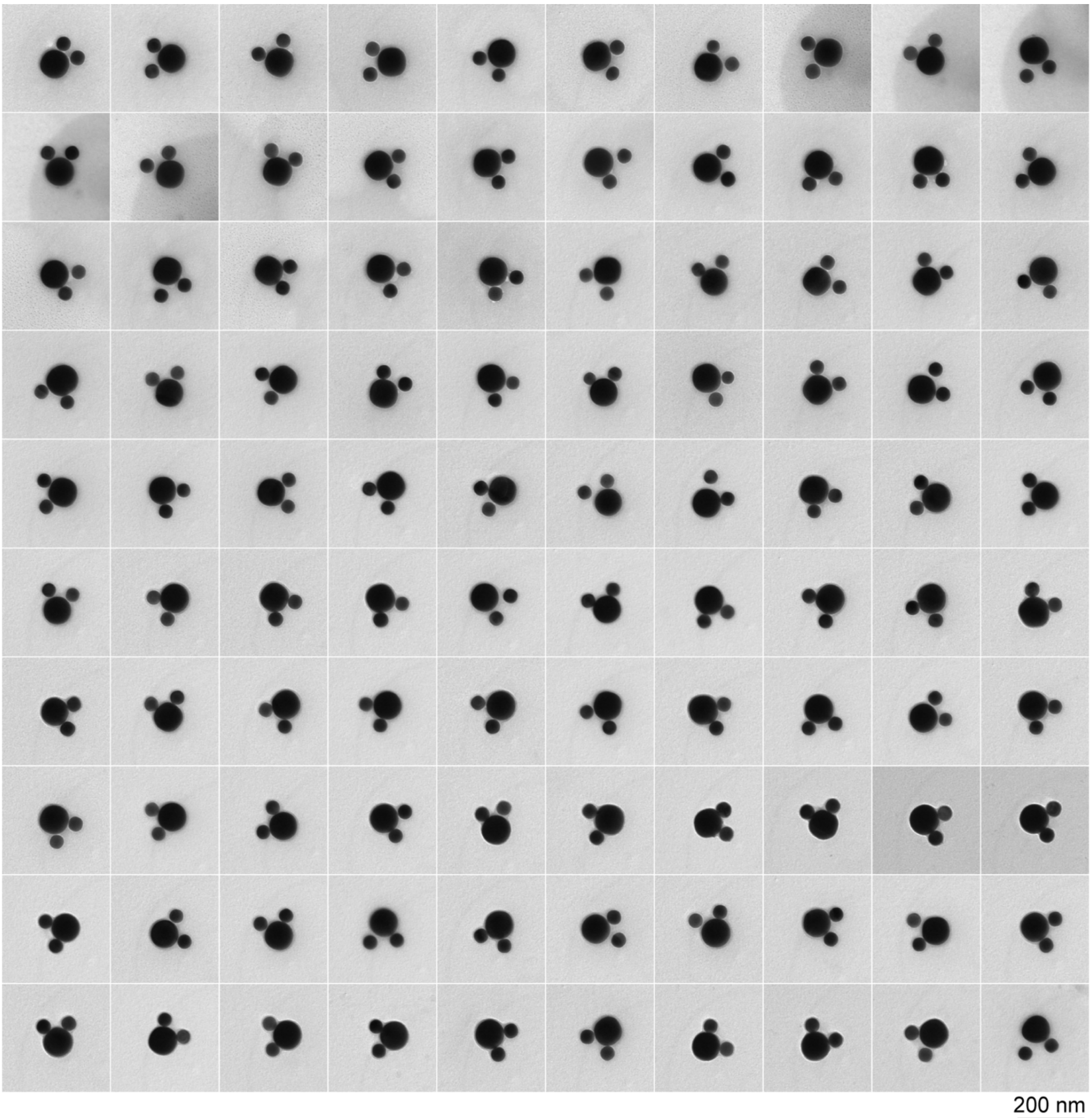

Fig. S17 | High-magnification TEM images of trimers consisting of one 100 nm Au NS and two 40 nm Au NSs. These independent stoichiometrically target-positive images were translationally and rotationally aligned and used to generate the aligned average shown in **Figure 3B** of the main manuscript. Source-image and instrument-control analyses of the recurrent fixed-pattern background are provided in Section 31.

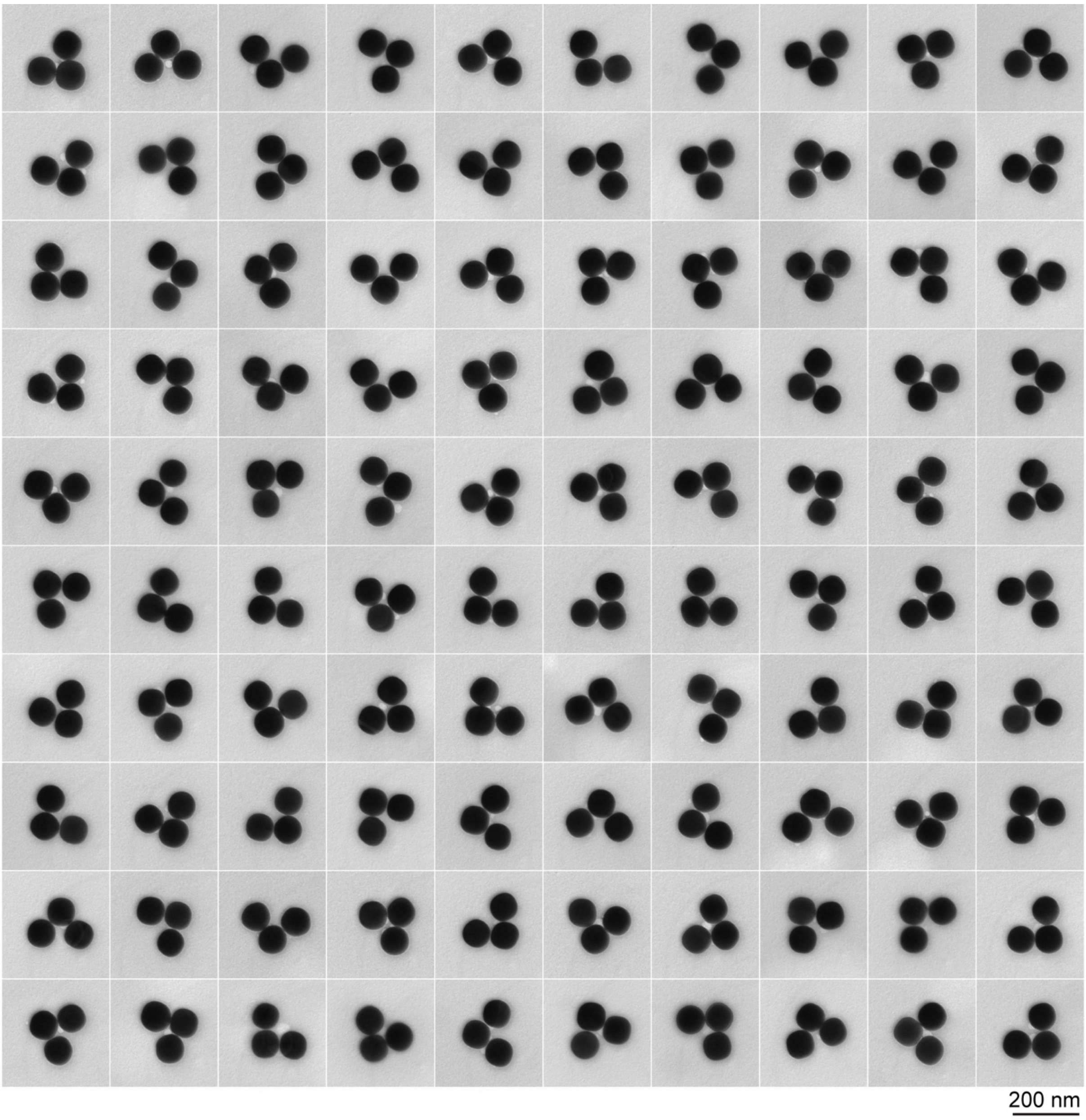

Fig. S18 | High-magnification TEM images of trimers consisting of three 100 nm Au NSs. These independent stoichiometrically target-positive images were translationally and rotationally aligned and used to generate the aligned average shown in **Figure 3C** of the main manuscript.

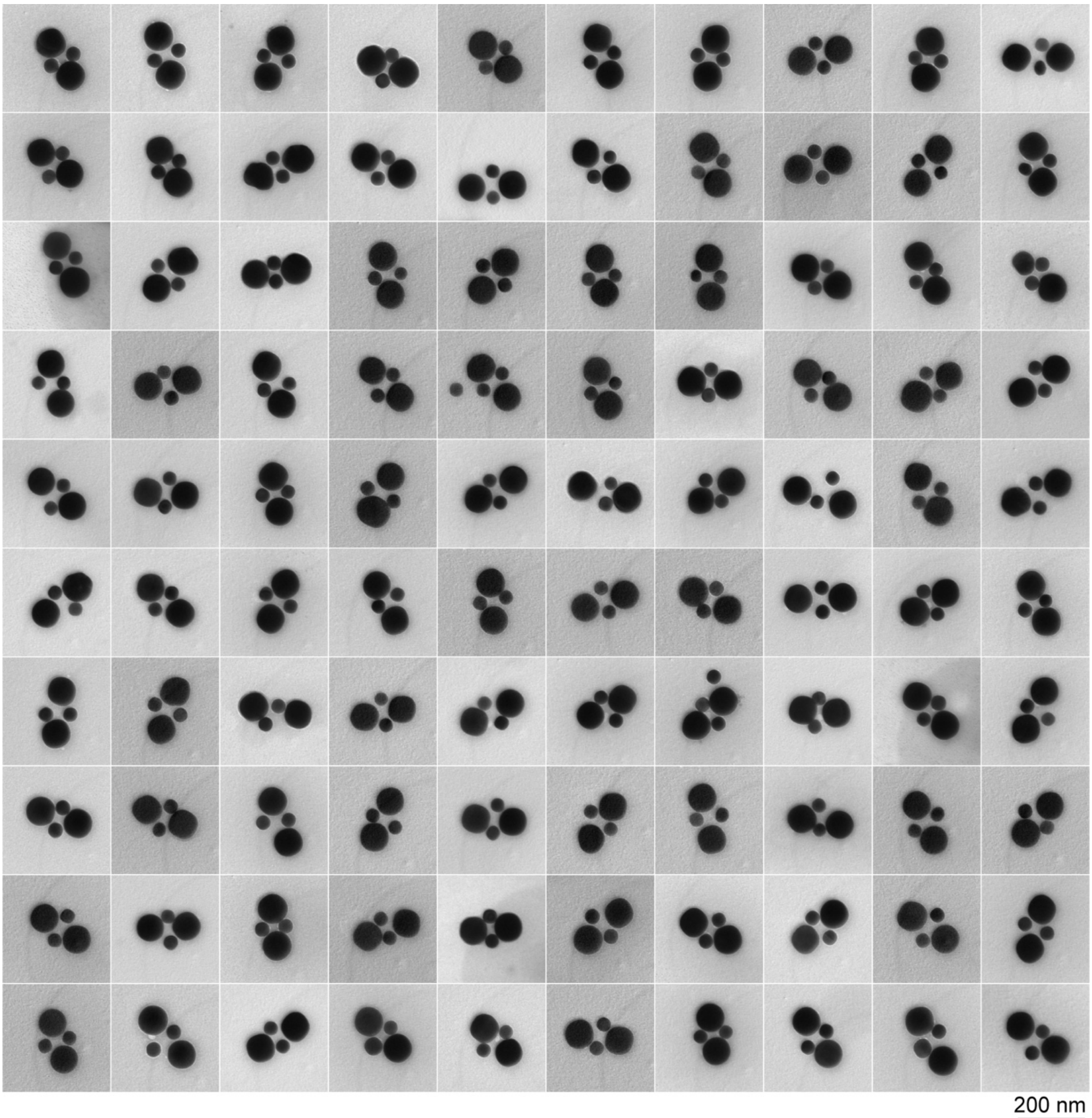

Fig. S19 | High-magnification TEM images of tetramers consisting of two 100 nm Au NSs and two 40 nm Au NSs. These independent stoichiometrically target-positive images were translationally and rotationally aligned and used to generate the aligned average shown in **Figure 3D** of the main manuscript.

18. Correlative TEM images and individual dark-field spectra of trimeric and tetrameric Au NS assemblies

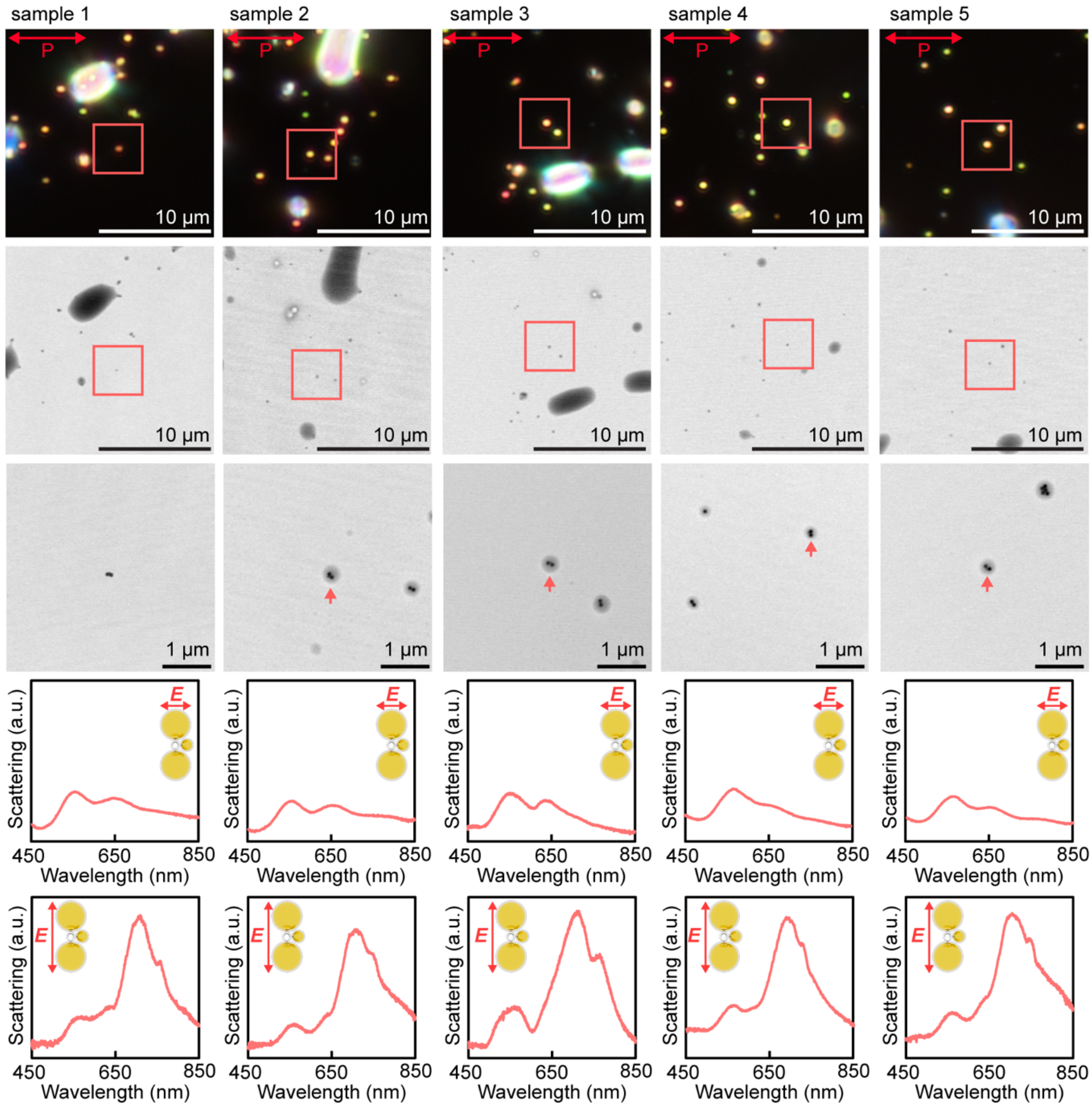

Fig. S20 | Correlative TEM images and dark-field scattering spectra of trimers consisting of two 100 nm Au NSs and one 40 nm Au NS. One-to-one correlative analysis of five displayed, structurally identified target nanocircuits in **Figure 4C** (Samples 1–5, marked by red boxes). From top to bottom, the first three rows represent: dark-field optical micrographs of individual nanocircuits with the incident polarization indicated by **P**, low-magnification TEM maps used for target localization, and intermediate-magnification TEM images used to determine the precise orientation of each nanocircuit. The bottom two rows show the corresponding polarization-resolved dark-field scattering spectra obtained under parallel (fourth row) and perpendicular (fifth row) excitation, respectively. The five displayed devices are the denominator for this figure; the targeted correlative workflow was not designed as an unbiased optical-yield survey.

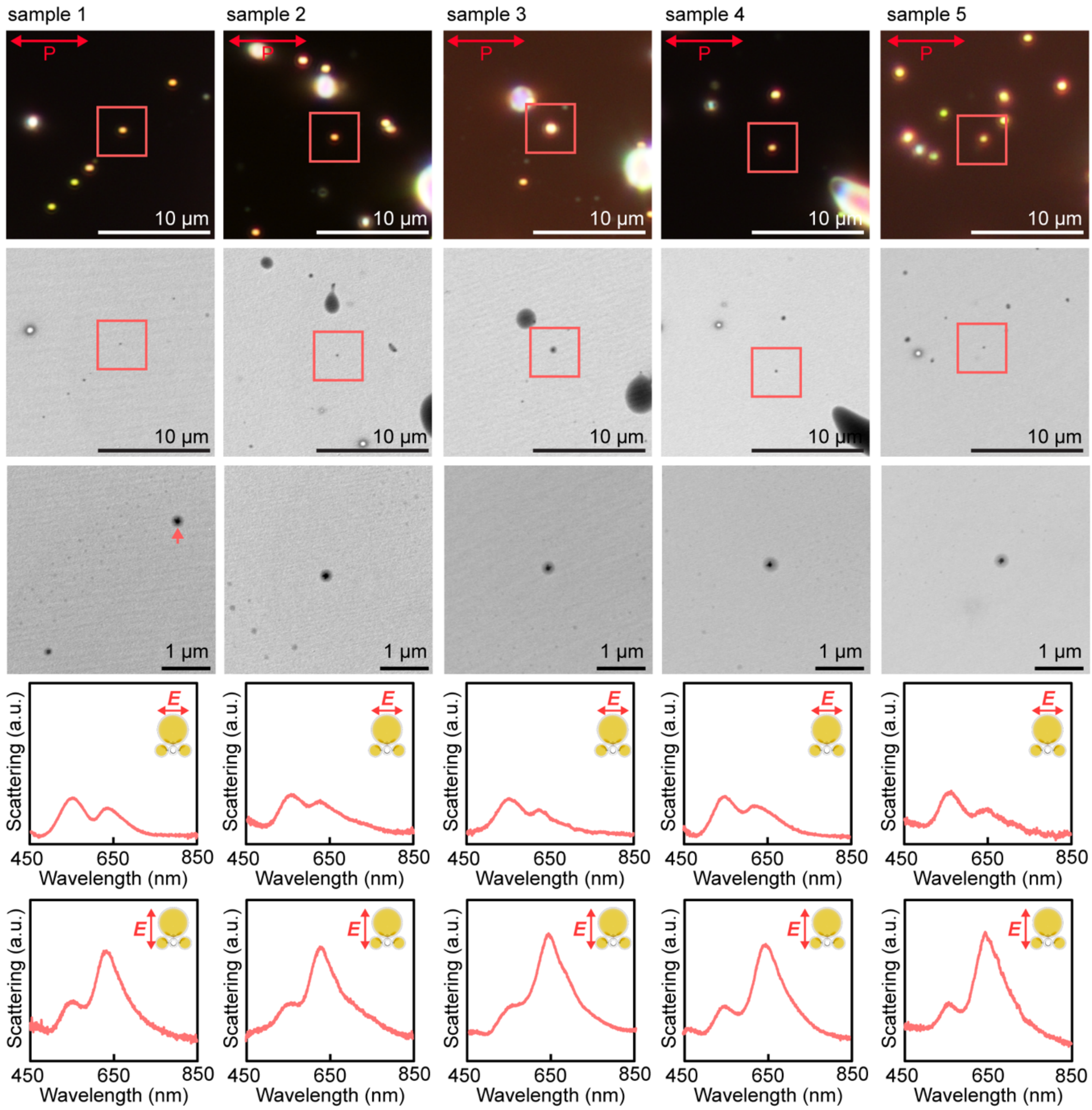

Fig. S21 | Correlative TEM images and dark-field scattering spectra of trimers consisting of one 100 nm Au NS and two 40 nm Au NSs. One-to-one correlative analysis of five displayed, structurally identified target nanocircuits in **Figure 4D** (Samples 1–5, marked by red boxes). From top to bottom, the first three rows represent: dark-field optical micrographs of individual nanocircuits with the incident polarization indicated by **P**, low-magnification TEM maps used for target localization, and intermediate-magnification TEM images used to determine the precise orientation of each nanocircuit. The bottom two rows show the corresponding polarization-resolved dark-field scattering spectra obtained under parallel (fourth row) and perpendicular (fifth row) excitation, respectively. The five displayed devices are the denominator for this figure; the targeted correlative workflow was not designed as an unbiased optical-yield survey.

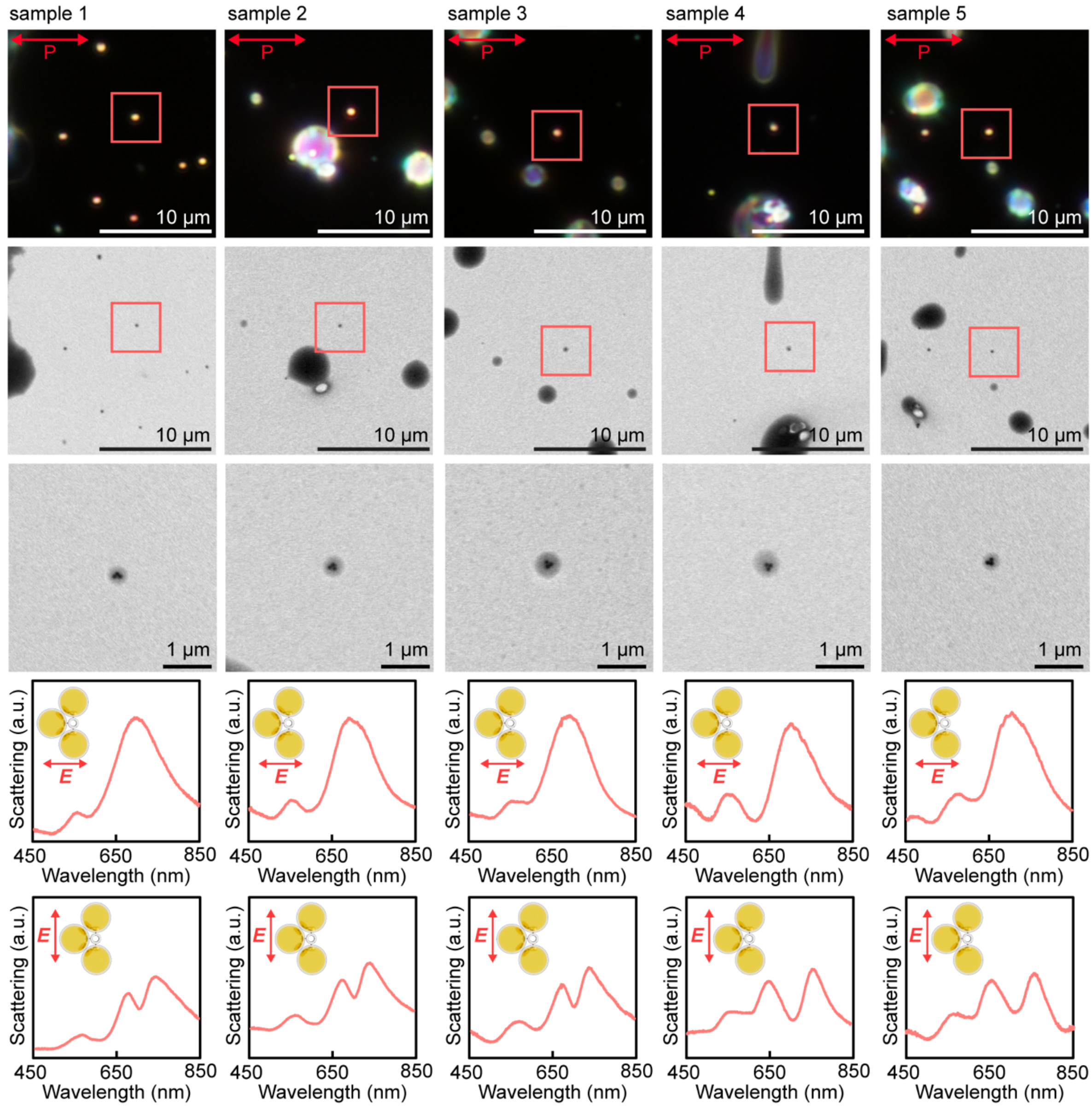

Fig. S22 | Correlative TEM images and dark-field scattering spectra of trimers consisting of three 100 nm Au NSs. One-to-one correlative analysis of five displayed, structurally identified target nanocircuits in **Figure 4E** (Samples 1–5, marked by red boxes). From top to bottom, the first three rows represent: dark-field optical micrographs of individual nanocircuits with the incident polarization indicated by **P**, low-magnification TEM maps used for target localization, and intermediate-magnification TEM images used to determine the precise orientation of each nanocircuit. The bottom two rows show the corresponding polarization-resolved dark-field scattering spectra obtained under parallel (fourth row) and perpendicular (fifth row) excitation, respectively. The five displayed devices are the denominator for this figure; the targeted correlative workflow was not designed as an unbiased optical-yield survey.

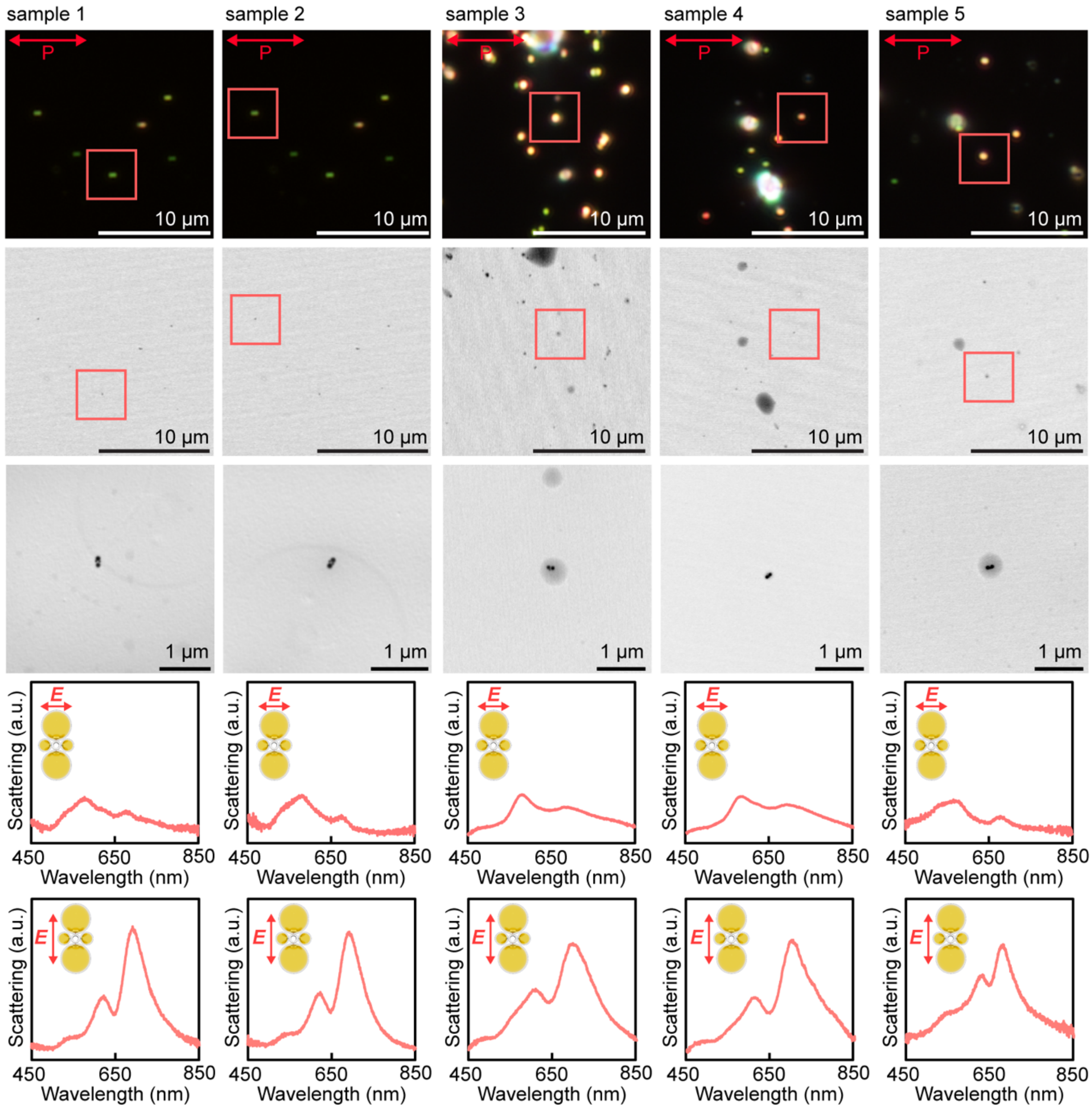

Fig. S23 | Correlative TEM images and dark-field scattering spectra of tetramers consisting of two 100 nm Au NSs and two 40 nm Au NSs. One-to-one correlative analysis of five displayed, structurally identified target nanocircuits in **Figure 4F** (Samples 1–5, marked by red boxes). From top to bottom, the first three rows represent: dark-field optical micrographs of individual nanocircuits with the incident polarization indicated by **P**, low-magnification TEM maps used for target localization, and intermediate-magnification TEM images used to determine the precise orientation of each nanocircuit. The bottom two rows show the corresponding polarization-resolved dark-field scattering spectra obtained under parallel (fourth row) and perpendicular (fifth row) excitation, respectively. The five displayed devices are the denominator for this figure; the targeted correlative workflow was not designed as an unbiased optical-yield survey.

19. *Q*-factor analysis of magnetic resonance modes

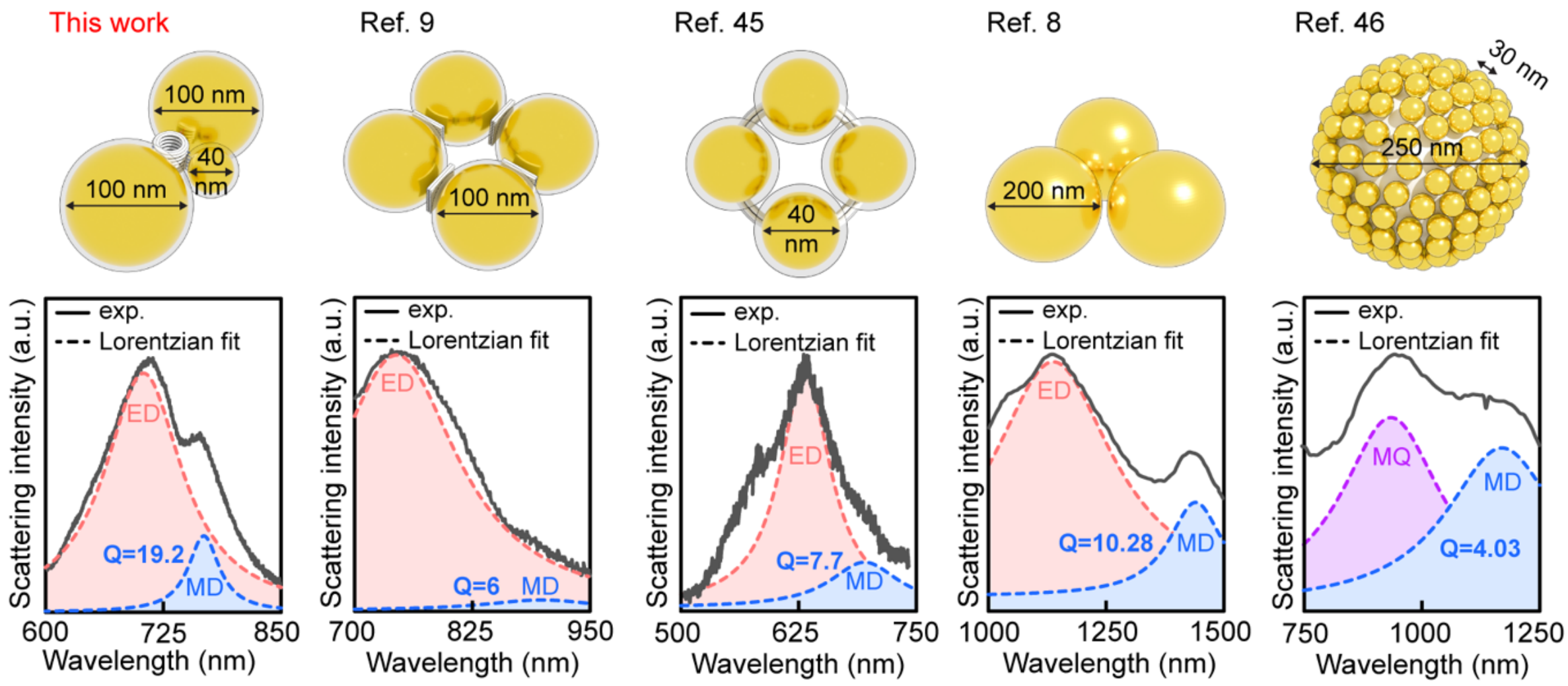

Fig. S24 | Benchmarking the Q-factor of the magnetic dipole (MD) mode of a trimeric Au NS cluster. (Top panel) Schematic illustrations of the trimeric Au NS cluster (this work) and other cluster geometries that exhibit MD mode (scaled schematic). (Bottom panel) Comparison of scattering spectra and their corresponding *Q*-factors. The spectrum for the trimer was measured in this work, while the spectra for the benchmark clusters were obtained from published data cited in the main manuscript. To ensure a consistent comparison, a Lorentzian function (dashed lines) was fitted to the resonance modes of each spectrum to extract and compare their respective *Q*-factors.

20. *Q*-factor analysis of the Fano resonance mode from the symmetry-broken trimer

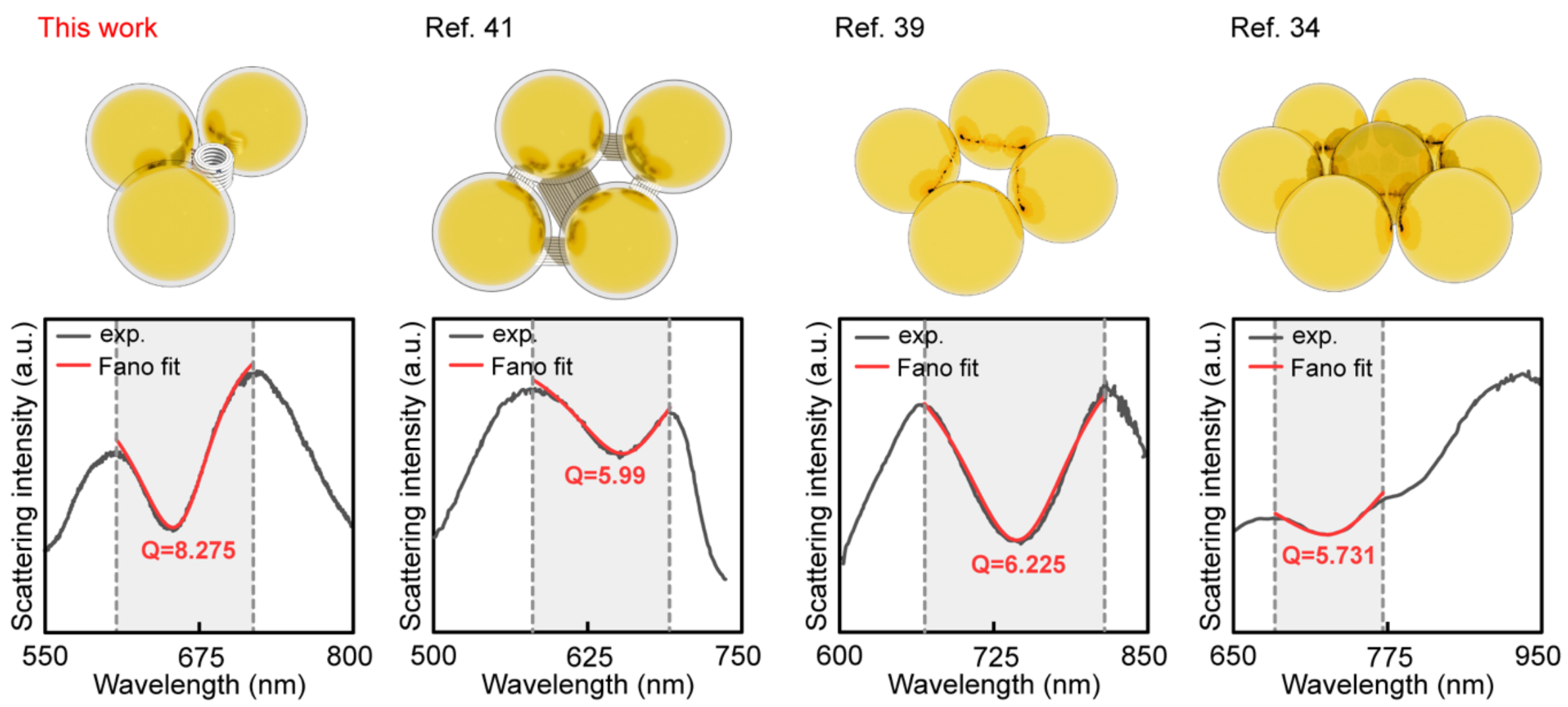

Fig. S25 | Benchmarking the *Q*-factor of the Fano resonance mode of a symmetry-broken Au NS trimer. (Top panel) Schematic illustrations of the symmetry-broken trimeric Au NS cluster (this work) and other cluster geometries that exhibit a Fano resonance mode. (Bottom panel) Comparison of scattering spectra and their corresponding *Q*-factors. The spectrum for the trimer was measured in this work, while the spectra for the benchmark clusters were obtained from published data cited in the main manuscript. To ensure a consistent comparison, a Fano function (red solid lines) was fitted to the resonance mode of each spectrum within the shaded grey region to extract and compare their respective quality factors.

21. High-magnification TEM images of multi-barrel systems

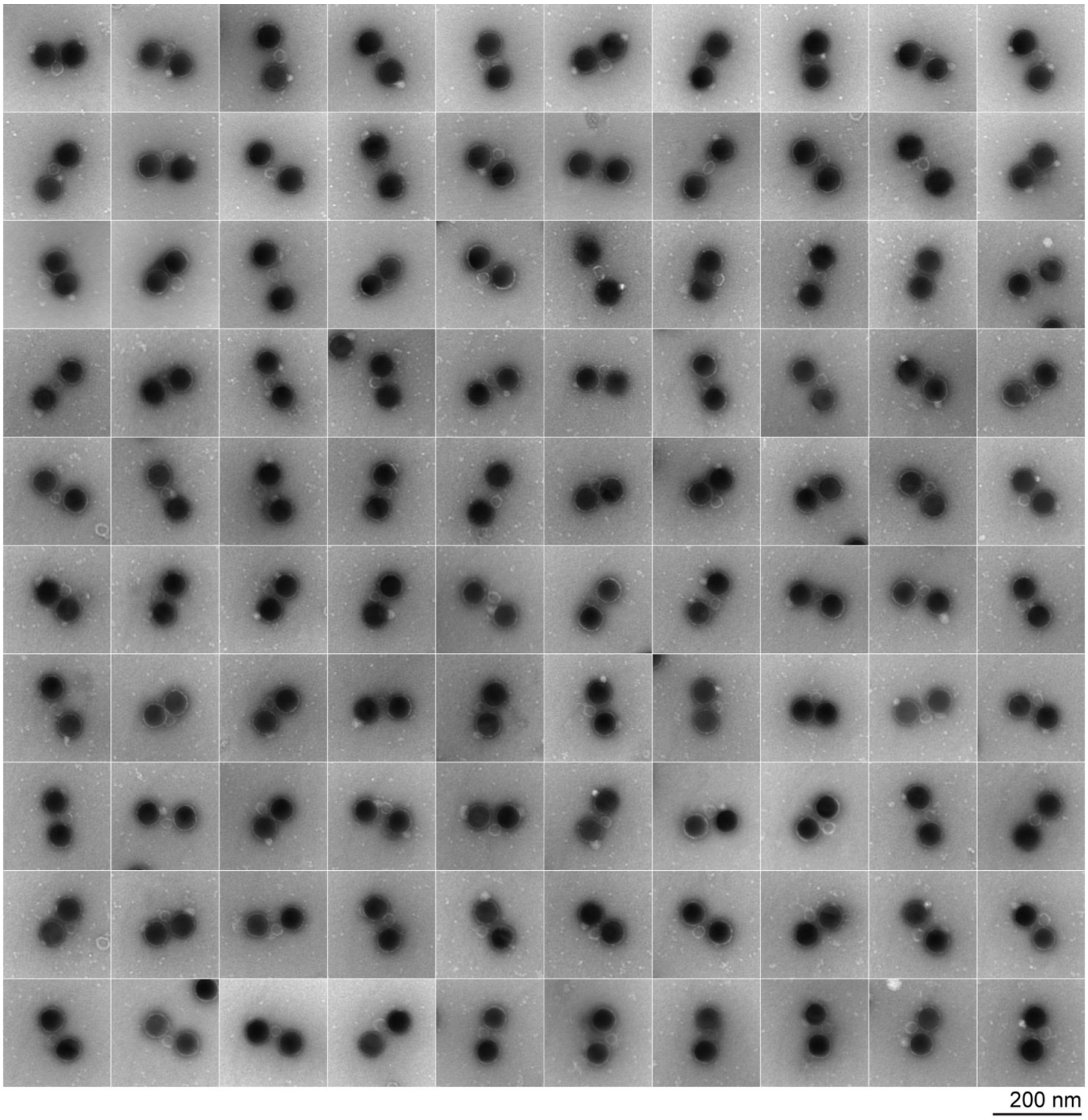

Fig. S26 | High-magnification TEM images of dimers consisting of two 60 nm Au NSs and a single barrel. The dimeric nanocircuit was assembled using a single-barrel design. These independent stoichiometrically target-positive images were translationally and rotationally aligned and used to generate the aligned average shown in **Figure 5A** of the main manuscript.

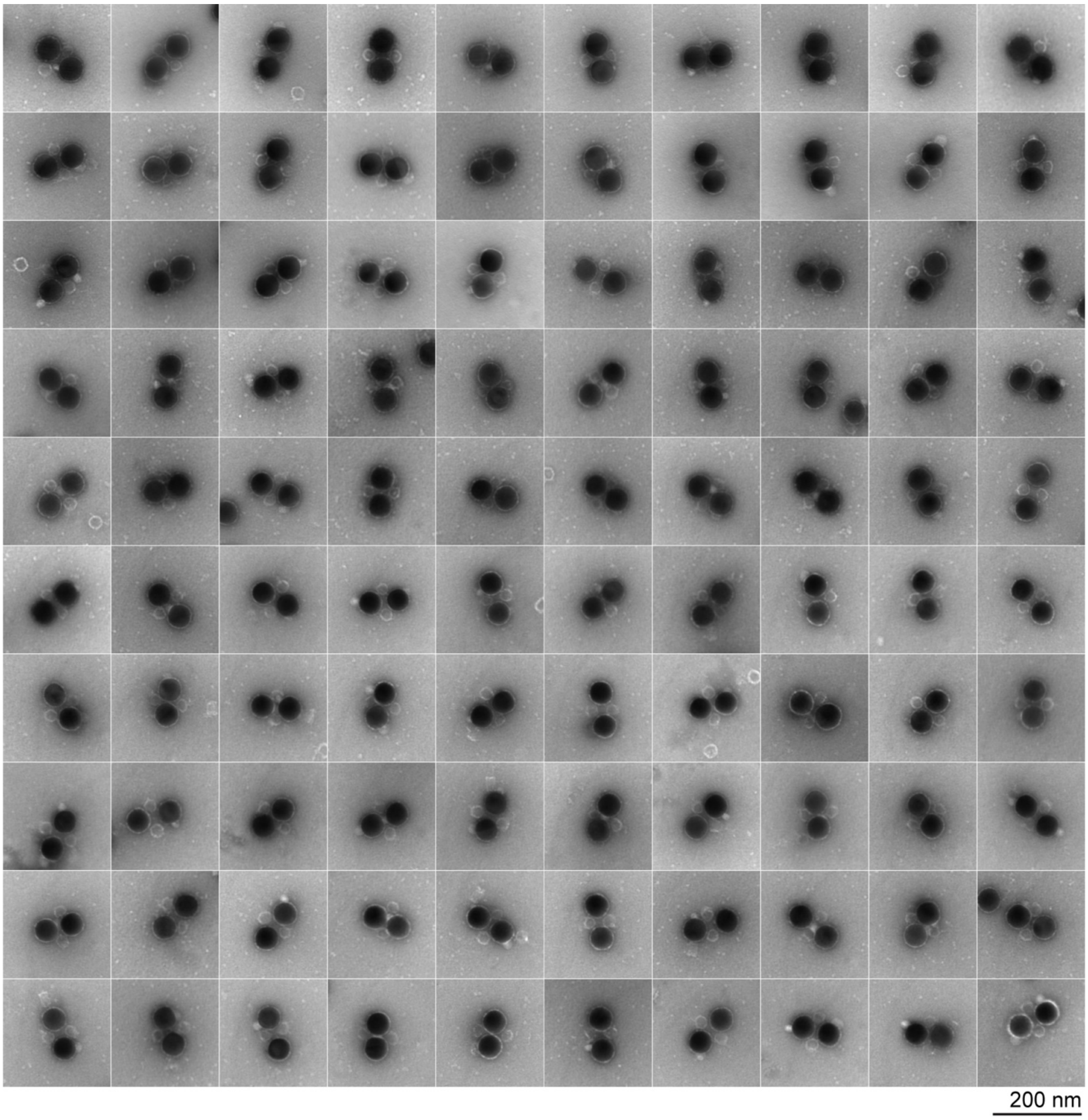

Fig. S27 | High-magnification TEM images of dimers consisting of two 60 nm Au NSs and a dual-barrel scaffold. The dimeric nanocircuit was assembled using a dual-barrel configuration. These independent stoichiometrically target-positive images were translationally and rotationally aligned and used to generate the aligned average shown in **Figure 5A** of the main manuscript.

22. Quantification of structural variability and controlled pivoting

To quantify reduced and controlled pivoting, we analyzed high-magnification TEM images (N = 100 stoichiometrically target-positive structures per motif). The handle–anti-handle duplex permits finite rotational freedom; the analysis therefore quantifies constraint-dependent variability rather than complete elimination. The 60 nm single-barrel dimer serves as a pivoting-sensitive stress test because its smaller contact footprint makes projected angular variability readily visible. The 60 nm dual-barrel dimer isolates the effect of adding a second geometric constraint. The 100 nm single-barrel dimer serves as a particle-size/contact-footprint reference using its individual gap distribution. For the diametrically opposed 100 nm dimer, however, the central barrel contour is obscured by the mass-thickness contrast of the two adjacent Au NSs, so an independent barrel-axis-based pivoting angle and lateral displacement cannot be assigned without imposing the model being tested.

Structural variability was quantified by (i) interparticle gap d, (ii) angular deviation θ relative to the designed barrel axis where that axis is directly resolvable, and (iii) lateral displacement δ from the designed attachment line. The direct 60 nm single-versus-dual comparison isolates the contribution of barrel number. The gap dispersion of the 100 nm single-barrel dimer provides a separate reference for particle size/contact footprint, while the 100 nm trimer provides a higher-order geometry in which angular residuals are measurable from the particle configuration. We note that this comparison provides complementary structural benchmarks rather than an isolated particle-size control, because the 60 nm dimers use adjacent attachment sites whereas the 100 nm reference dimers adopt diametrically opposed sites. **Figure S28** and **Table S7** summarize these complementary comparisons.

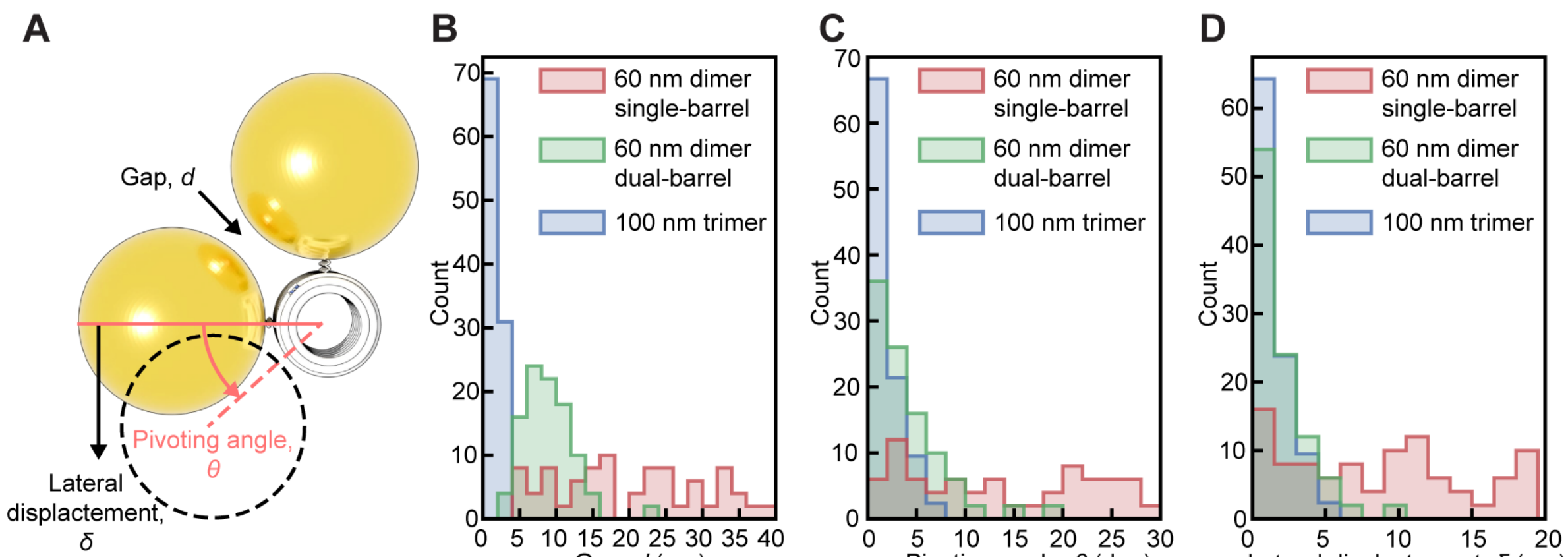

Fig. S28 | Quantitative comparison of apparent pivoting and nanogap variability. (A) Definitions of interparticle gap d, pivoting angle θ, and lateral displacement δ. (B–D) Distributions of the three metrics for 60 nm single-barrel dimers, 60 nm dual-barrel dimers, and the 100 nm single-barrel trimer (N = 100 stoichiometrically target-positive structures per motif). **Table S7** additionally reports the 100 nm single-barrel dimer gap distribution; θ and δ are not assigned for this dimer because the barrel axis is not independently resolvable in its diametric two-particle TEM projection.

Table S7 | Quantitative comparison of apparent pivoting and nanogap variability, including the 100 nm single-barrel dimer gap reference. Values are mean ± SD.

Assembly	N	Gap, d (nm)	Pivoting angle, θ (deg)	Lateral displacement, δ (nm)
60 nm single-barrel stress-test dimer	100	22.2 ± 11.8	18.8 ± 13.2	8.6 ± 5.8
60 nm dual-barrel dimer	100	7.9 ± 2.5	3.9 ± 3.7	1.9 ± 1.7
100 nm single-barrel dimer (main nanocircuit)	100	21.9 ± 3.6	Not independently resolvable	Not independently resolvable
100 nm single-barrel trimer (main nanocircuit)	100	1.8 ± 0.5	1.8 ± 1.5	1.4 ± 1.2

23. Correlative TEM images and individual dark-field spectra of multi-barrel systems

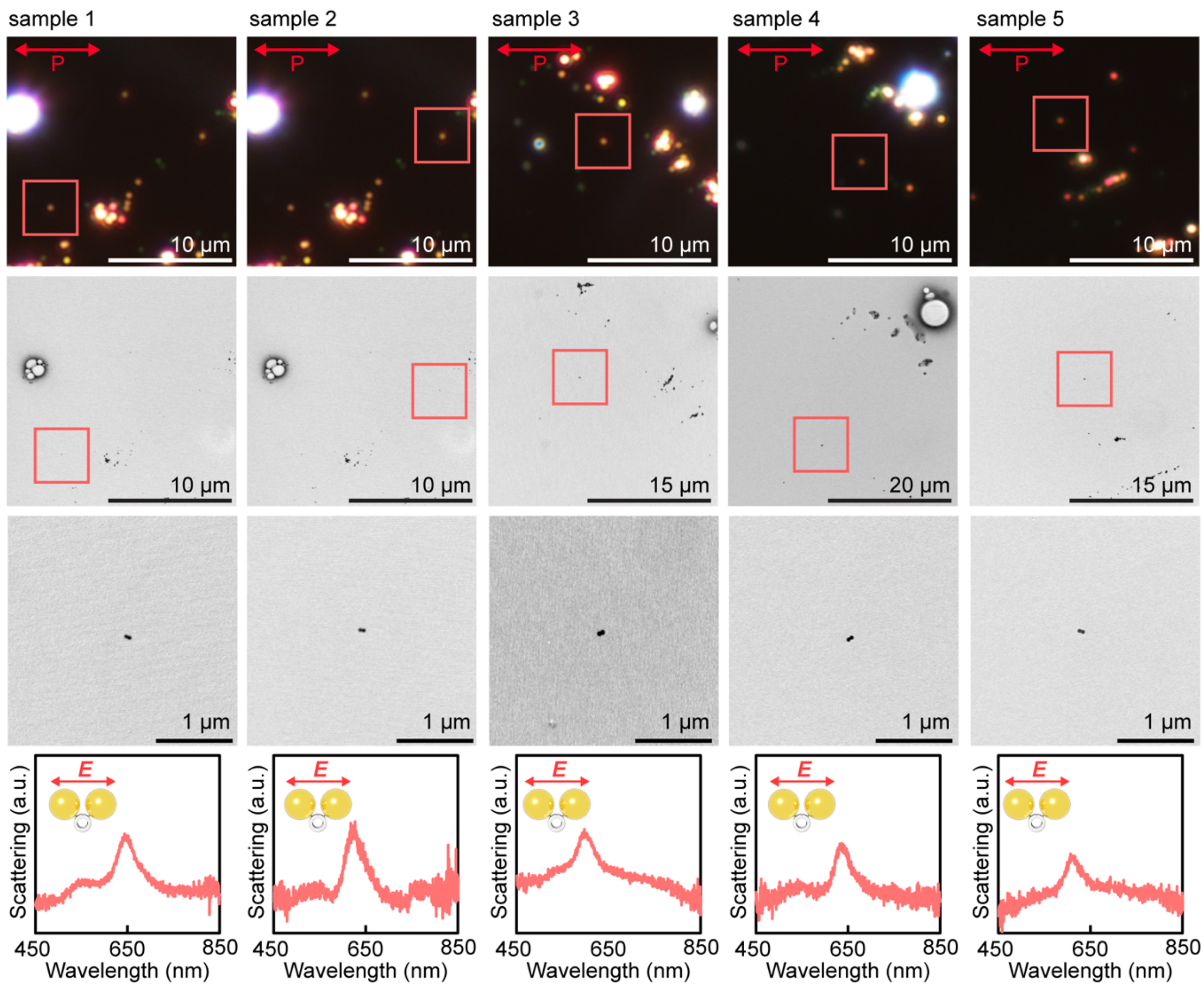

Fig. S29 | Correlative TEM images and dark-field scattering spectra of dimers consisting of two 60 nm Au NSs and a single barrel. One-to-one correlative analysis of five displayed, structurally identified target nanocircuits in **Figure 5B** (Samples 1–5, marked by red boxes). From top to bottom, the first three rows represent: dark-field optical micrographs of individual nanocircuits with the incident polarization indicated by **P**, low-magnification TEM maps used for target localization, and intermediate-magnification TEM images used to determine the precise orientation of each nanocircuit. The bottom row shows the corresponding polarization-resolved dark-field scattering spectra obtained under parallel excitation. The five displayed devices are the denominator for this figure; the targeted correlative workflow was not designed as an unbiased optical-yield survey.

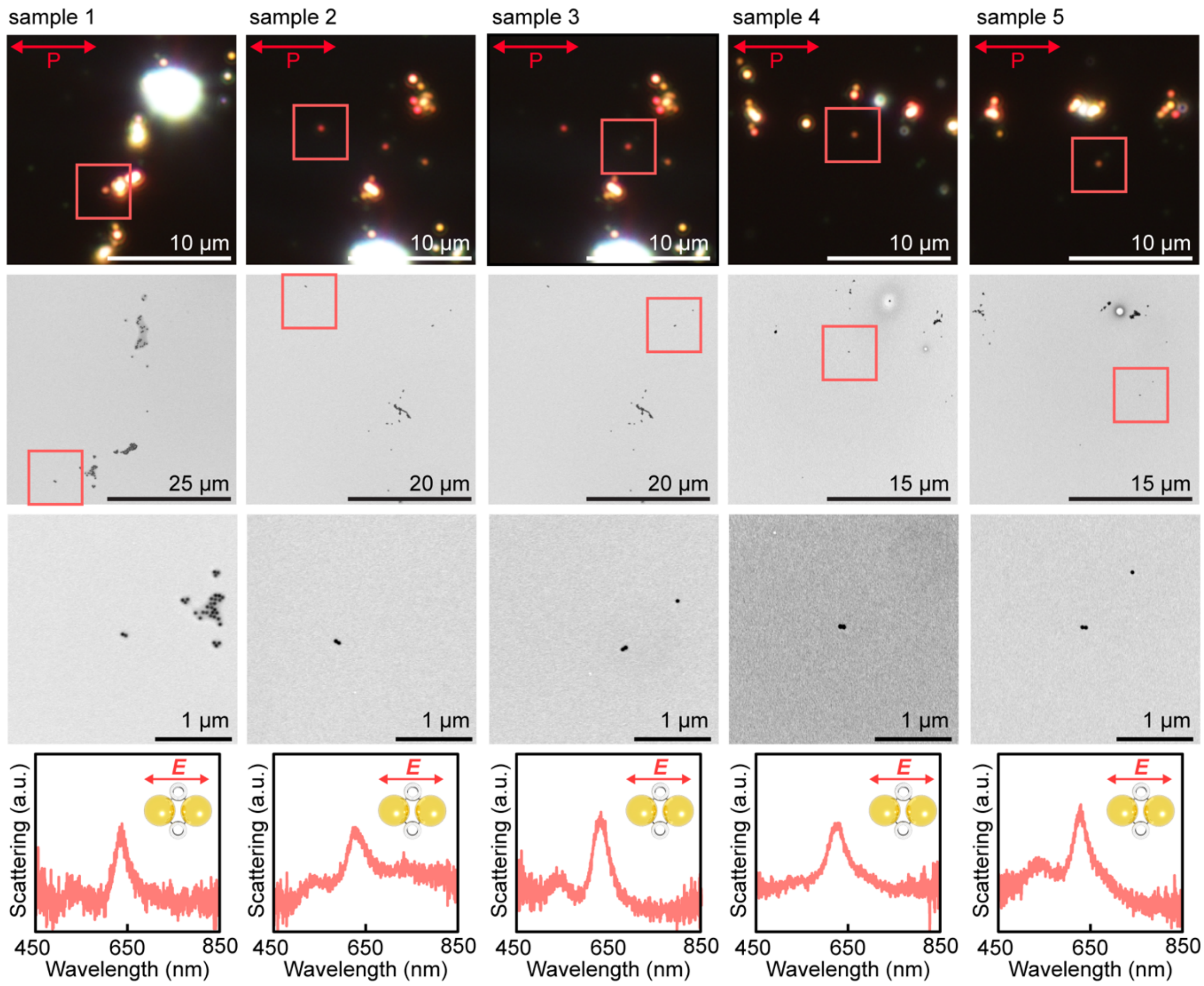

Fig. S30 | Correlative TEM images and dark-field scattering spectra of dimers consisting of two 60 nm Au NSs and a dual-barrel scaffold. One-to-one correlative analysis of five displayed, structurally identified target nanocircuits in **Figure 5B** (Samples 1–5, marked by red boxes). From top to bottom, the first three rows represent: dark-field optical micrographs of individual nanocircuits with the incident polarization indicated by **P**, low-magnification TEM maps used for target localization, and intermediate-magnification TEM images used to determine the precise orientation of each nanocircuit. The bottom row shows the corresponding polarization-resolved dark-field scattering spectra obtained under parallel excitation. The five displayed devices are the denominator for this figure; the targeted correlative workflow was not designed as an unbiased optical-yield survey.

24. Additional TEM images of 3D optical nanocircuits

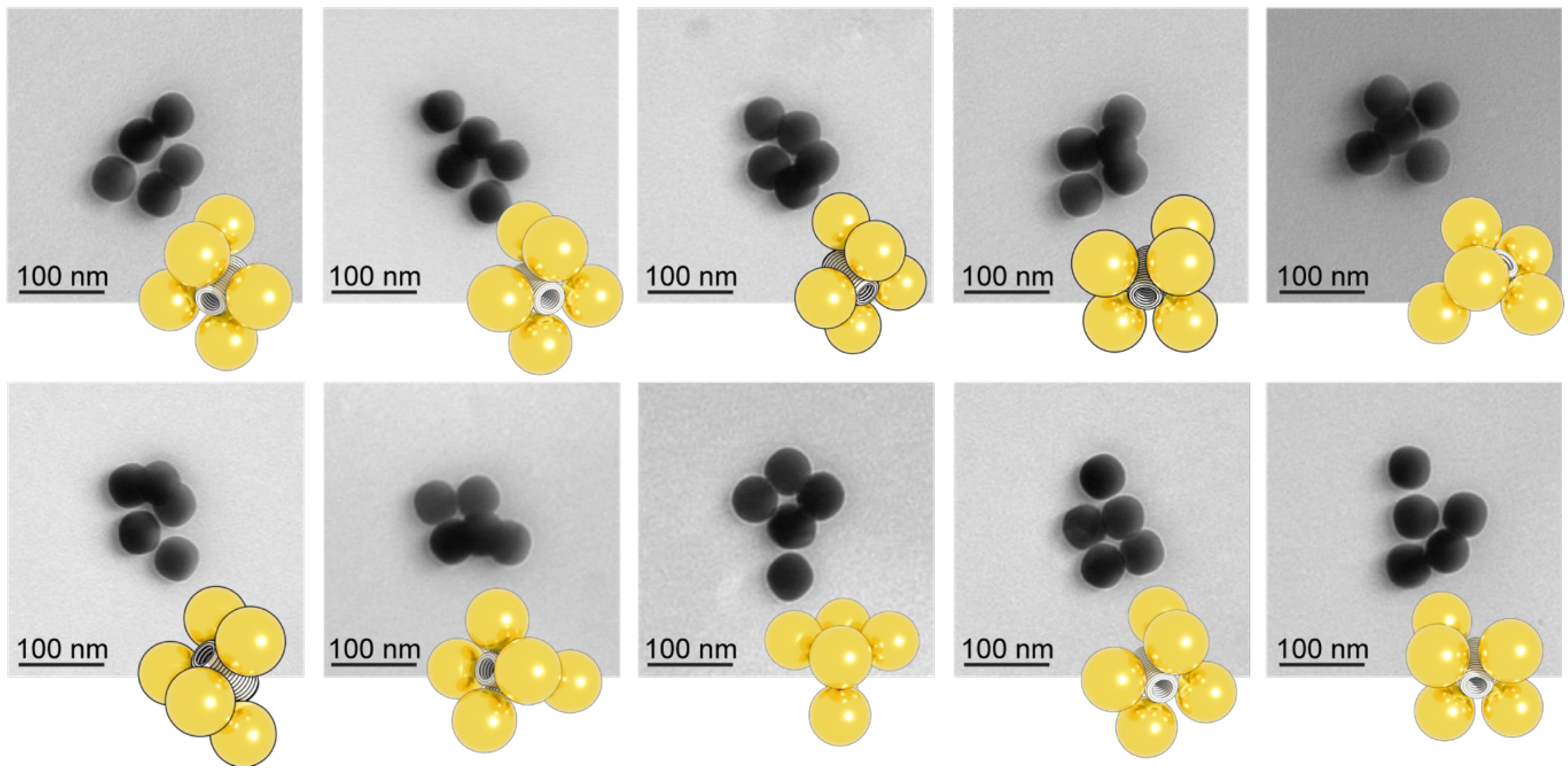

Fig. S31 | High-magnification TEM images of 3D pentamers consisting of five 60 nm Au NSs. The pentameric nanocircuit was assembled using a vertically stacked dual-barrel configuration. Ten stoichiometrically target-positive structures are shown.

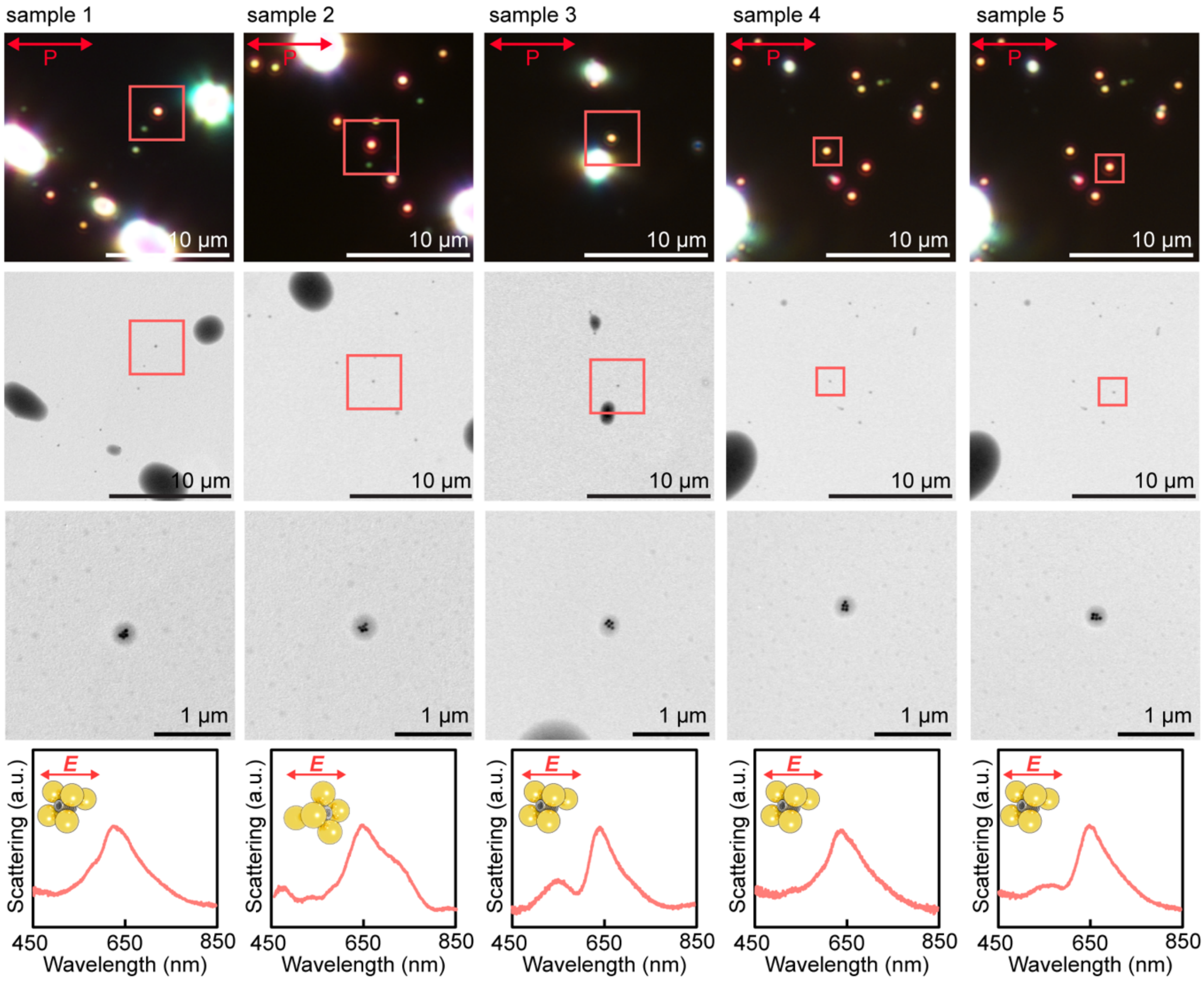

Fig. S32 | Correlative TEM images and dark-field scattering spectra of 3D pentamers consisting of five 60 nm Au NSs. One-to-one correlative analysis of five displayed, structurally identified target nanocircuits in **Figure 5F** (Samples 1–5, marked by red boxes). From top to bottom, the first three rows represent: dark-field optical micrographs of individual nanocircuits with the incident polarization indicated by **P**, low-magnification TEM maps used for target localization, and intermediate-magnification TEM images used to determine the precise orientation of each nanocircuit. The bottom row shows the corresponding polarization-resolved dark-field scattering spectra obtained under horizontal excitation. The five displayed devices are the denominator for this figure; the targeted correlative workflow was not designed as an unbiased optical-yield survey.

25. TEM image alignment, processing, and interpretation

Two distinct image processing and averaging workflows were employed depending on the target motif. For the Au-NS-loaded assemblies (**Figures 2, 3, and 5**), panels described as aligned averages were generated from high-magnification crops in Fiji (ImageJ). Each crop was translated and rotated to place the particle principal axis on a common reference, followed by a pixel-wise arithmetic mean. No unsupervised classification was performed, and no subclass was selected after alignment; 'aligned average' is therefore the technically accurate term for these structures. Meanwhile, for the Au-NS-free bare barrel origami (**Figure 1C**), high-magnification images were processed using the Scipion software framework (Xmipp package) for 2D classification and class averaging to appropriately separate and average the distinct top and side views. We therefore use the term 'class average' for this specific panel.

All of these averaged panels visualize the mean projected geometry only. For the Au-NS-free barrel in **Figure 1C**, structural dimensions were measured from individual high-magnification crops before classification and averaging: inner and outer diameters from 100 top views and height from 100 side views, as detailed in Section 30, Supplementary Information. Likewise, the Au NS gap statistics in the main figures were calculated from individual structures rather than from averaged-image edges. The N = 100 Au NS galleries are datasets of structures possessing the correct stoichiometry obtained after gel purification and therefore describe geometry conditional on the intended stoichiometry rather than population-wide target yield.

A recurrent broad dark feature in some Au NS TEM crops was identified as an instrument-associated fixed-pattern response. Across uncropped source images, random Formvar-film features and specimen context vary from field to field, whereas the broad shadow remains at a reproducible detector-relative position; a similar detector-centered feature is also observed in a blank acquisition without a specimen or TEM grid (Section 31, Supplementary Information). These observations, together with the dependence on accumulated focused-beam exposure, are consistent with localized sensitivity variation of the scintillator/detector system. Because each target was centered at approximately the same detector position, the feature appears at a similar coordinate in identically sized crops. The Au NS mass-thickness contrast is substantially greater than this faint low-frequency background, and particle-edge and gap measurements were based on the Au NS boundaries. Image processing was limited to whole-image cropping and rotation; no local intensity adjustment, local filtering, or background subtraction was applied.

26. Design geometries and nominal nanogaps

The nominal gaps used in the design schematics were derived from the 30 nm barrel diameter, programmed handle positions, Au NS radii, and the designed barrel-to-particle surface distance. The 3 × 100 nm trimer uses a longer handle–anti-handle pair to permit assembly of three large particles; the other motifs use the shorter pairing to place the Au NSs close to the barrel wall. These nominal values are design quantities and are distinct from projected dry-state TEM gaps and device-specific FEM inputs.

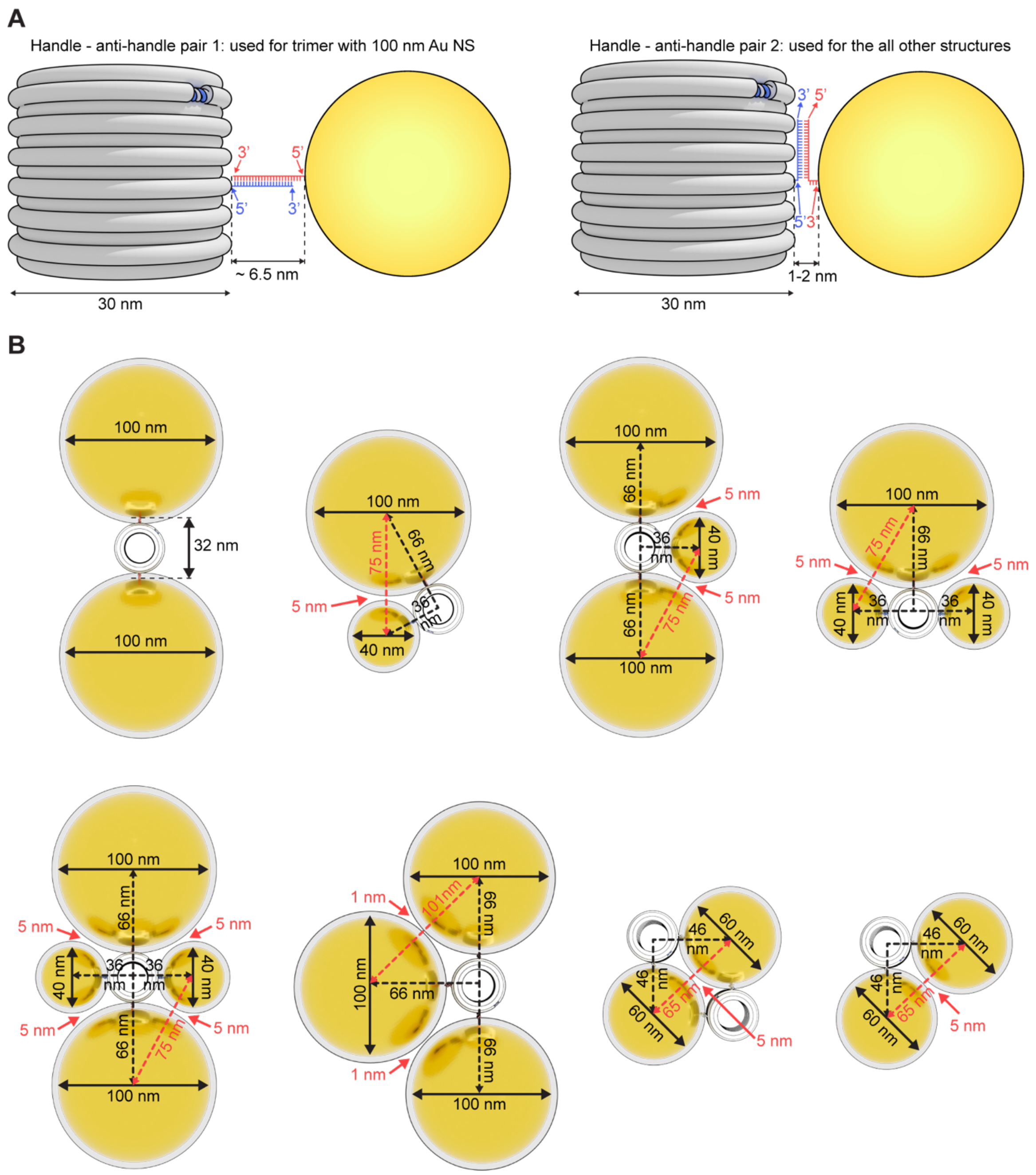

Fig. S33 | Design geometries and nominal nanogaps. (A) Handle–anti-handle pairing geometries. The longer pairing is used for the 3 × 100 nm trimer; the shorter pairing is used for the other motifs. (**B**) Dimensioned schematics showing the 30 nm barrel, handle positions, Au NS diameters, radial center distances, and nominal edge-to-edge gaps.

27. Device-specific geometries used for FEM

For each device-specific FEM curve included in the uncertainty analysis, particle diameters and interparticle gaps were measured from the TEM image of that same individual nanocircuit. **Figure S34** labels the measurement positions; **Table S8** lists the resulting inputs. Five technical gap-reading replicates were performed by one operator on the same TEM image, with the preceding numerical values hidden until all measurements were completed. The reported mean ± standard deviation (s.d.) therefore quantifies within-operator analysis repeatability rather than instrumental resolution or independent structural variability. The mean gap values were used for the baseline FEM curves. Lower- and upper-bound calculations were then generated by setting the complete set of measured gaps for each device simultaneously to their mean − s.d. and mean + s.d. values while retaining the measured particle diameters and all other simulation parameters. At each wavelength, the shaded envelope in **Figure S35** spans the minimum-to-maximum SCS among the lower-bound, baseline, and upper-bound calculations. These envelopes quantify sensitivity to empirical gap-reading repeatability; they are not statistical confidence intervals and do not include uncertainty in particle shape, orientation, dielectric functions, or substrate modeling. The 60 nm single-barrel curve in **Figure 5B** is a reference calculation rather than a one-to-one device-specific calculation and is therefore not assigned a device-specific uncertainty envelope. These device-specific values are independent of the nominal gap shown in the design schematic and of the $N = 100$ population mean.

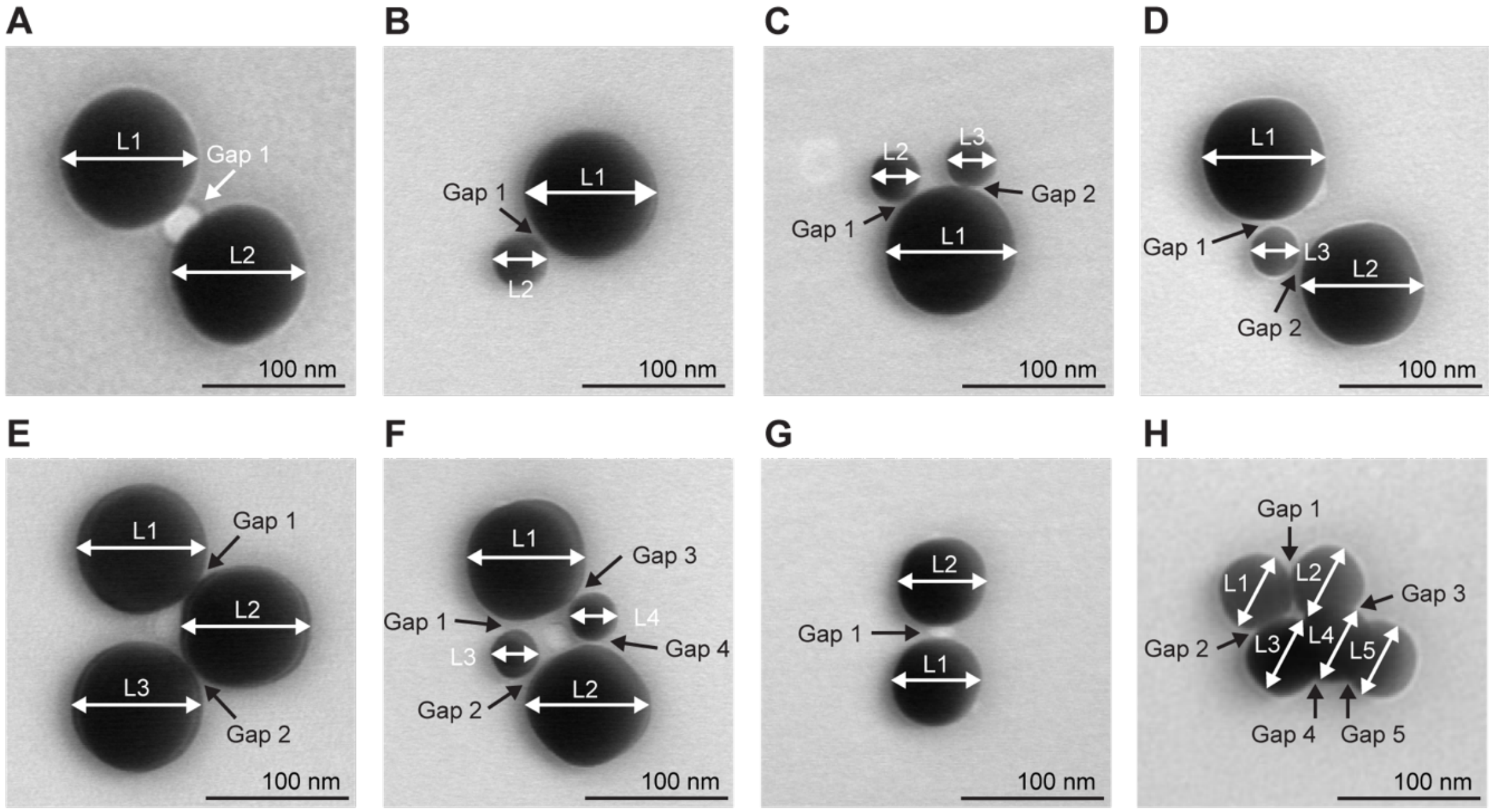

Fig. S34 | TEM images of the individual nanocircuits used for device-specific geometric parameter extraction. (**A**) 2 × 100 nm dimer; (**B**) 100/40 nm dimer; (**C**) trimer composed of 1 × 100 nm and 2 × 40 nm Au NSs; (**D**) trimer composed of 2 × 100 nm and 1 × 40 nm Au NSs; (**E**) 3 × 100 nm trimer; (**F**) 2 × 100 nm + 2 × 40 nm tetramer; **(G)** 2 × 60 nm dual-barrel dimer; and (**H**) 5 × 60 nm pentamer. Labels L1–L5 and Gap 1–Gap 5 identify the exact measurements reported in **Table S8**.

To evaluate the statistical position of each correlative device within the overall assembly

statistics, each gap was standardized against the corresponding $N = 100$ population distribution as $z = (g_{\text{device}} - \mu_{\text{pop}})/\sigma_{\text{pop}}$ (**Table S8**), where g_{device} is the mean measured gap of the individual device, and μ_{pop} and σ_{pop} are the mean and s.d. of the corresponding population, respectively. Devices A–D and F–G fall within 1.25 standard deviations of their respective population means (−1.25 to −0.11 s.d.). Device E (3 × 100 nm trimer) lies in the open-gap tail at +2.60 to +2.80 s.d. relative to its population (1.8 ± 0.5 nm), representing an individual nanocircuit with wider interparticle gaps.

Table S8 | Device-specific Au NS diameters (L) and interparticle gaps used as FEM inputs. All values are in nanometers and correspond to **Figure S34**. Motif G corresponds to the 60 nm dual-barrel dimer. For motif H, population z-score is not assigned because the population gap distribution was not quantified for the 3D structure. The ± values are within-operator standard deviations from five technical gap-reading replicates on the same TEM image and do not represent independent structural replicates.

Motif	L1	L2	L3	L4	L5	Gap 1	Gap 2	Gap 3	Gap 4	Gap5	Gap z-scores
A	96.6	96.6	-	-	-	17.4 ± 0.2	-	-	-	-	− 1.25
B	93.0	37.2	-	-	-	4.9 ± 0.2	-	-	-	-	− 0.26
C	93.8	35.8	35.8	-	-	3.3 ± 0.2	3.4 ± 0.4	-	-	-	− 0.72 to − 0.68
D	89.0	89.0	36.0	-	-	3.1 ± 0.1	3.7 ± 0.2	-	-	-	− 0.84 to − 0.65
E	90.4	90.4	90.4	-	-	3.2 ± 0.3	3.1 ± 0.3	-	-	-	+ 2.60 to + 2.80
F	87.7	87.7	36.0	36.0	-	5.2 ± 0.3	4.9 ± 0.3	3.0 ± 0.3	3.1 ± 0.1	-	− 0.89 to − 0.11
G	60.1	61.3	-	-	-	6.7 ± 0.2	-	-	-	-	− 0.48
H	60.0	60.0	60.0	60.0	60.0	2.4 ± 0.3	2.4 ± 0.3	2.4 ± 0.3	2.4 ± 0.3	2.4 ± 0.3	N/A

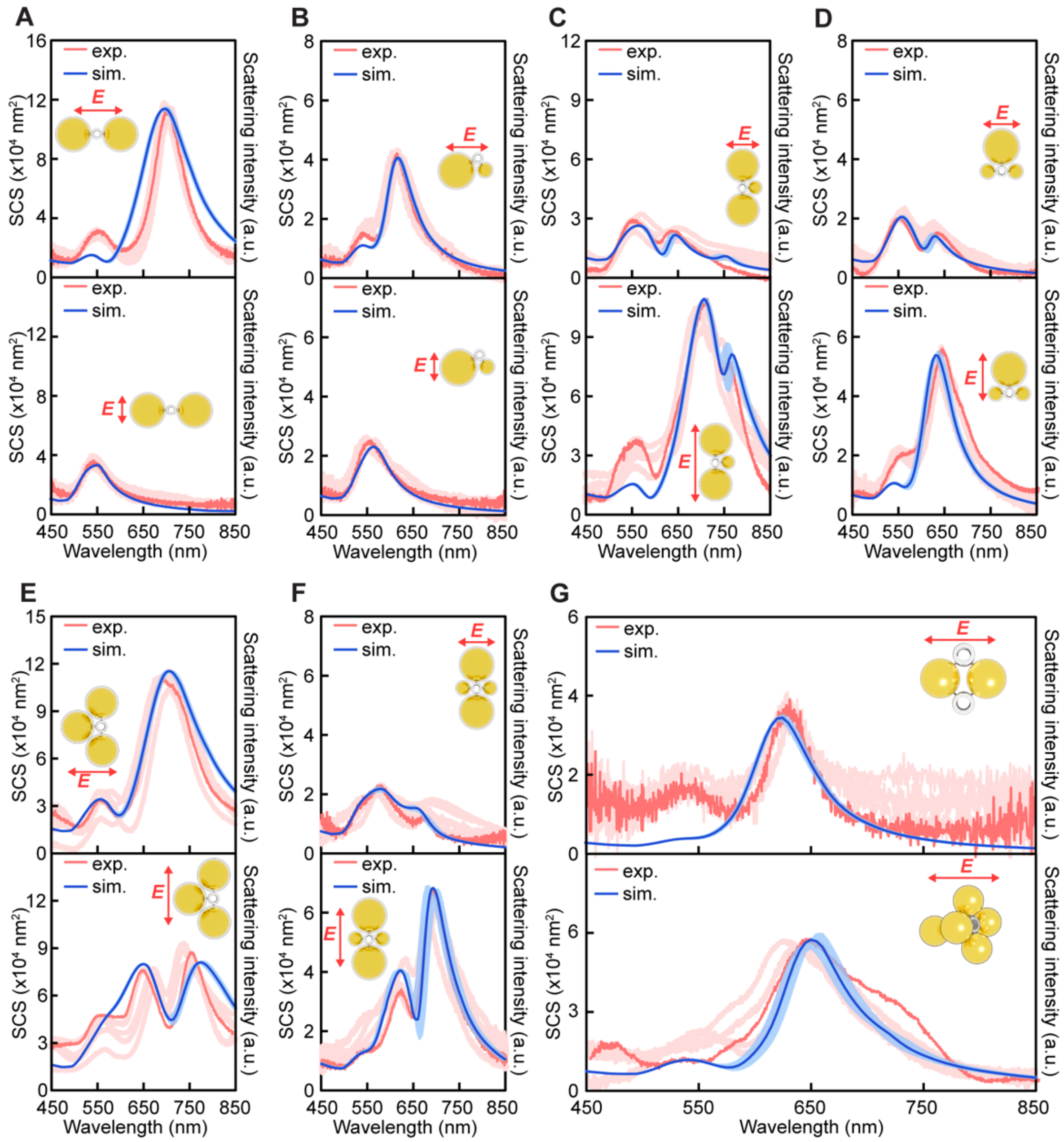

Fig. S35 | FEM sensitivity to empirical gap-reading repeatability. (A–G) Polarization-resolved DF spectra (red; five devices per motif) compared with device-specific baseline FEM calculations (blue) and shaded gap-reading sensitivity envelopes for **(A)** 2 × 100 nm dimer, **(B)** 100/40 nm dimer, **(C)** 2 × 100 nm + 1 × 40 nm trimer, **(D)** 1 × 100 nm + 2 × 40 nm trimer, **(E)** 3 × 100 nm trimer, **(F)** 2 × 100 nm + 2 × 40 nm tetramer, and **(G)** 2 × 60 nm dual-barrel dimer (top) and 5 × 60 nm pentamer (bottom). Lower and upper calculations set all measured gaps simultaneously to mean − SD and mean + SD; the envelope is the wavelength-wise minimum-to-maximum SCS across these curves and the baseline. SD values derive from five technical gap-reading replicates on the same TEM image (**Table S8**). The envelopes are sensitivity ranges, not confidence intervals or a complete FEM uncertainty budget. The 60 nm single-barrel reference in **Figure 5B** is excluded because no same-device repeat-measured geometry was available.

28. Loaded-state geometry and projected dry-state nanogaps

Directly resolving the central DNA barrel in assemblies containing two to four 100 nm Au NSs is not reliable in conventional TEM because the mass-thickness contrast of the adjacent Au particles dominates and overlaps the projected DNA signal. Conventional staining may increase DNA contrast, but in this geometry, it would not guarantee independent segmentation of a barrel located immediately between the strongly scattering Au NS projections. We therefore assess loaded-state geometry from the spatial distribution of the Au NSs: particle diameters, center positions, edge-to-edge gaps, and, where the barrel axis is resolvable, angular and lateral residuals. This is an operational measure of the geometry maintained under the same dried-grid condition used for the correlative TEM–DF analysis.

The observed interparticle gap variations exhibit a distinct motif-dependent pattern. While the principal adjacent-site motifs assembled from 40 and/or 100 nm Au NSs show projected gaps close to their nominal design values, the diametric 2 × 100 nm dimer exhibits a pronounced gap contraction (21.9 ± 3.6 nm observed vs ~ 30 nm nominal). This contraction is consistent with a support-limited top-rim contact geometry on the substrate and does not require barrel collapse as its primary explanation.

Let R_b = 15.45 nm be the measured barrel outer radius (outer diameter 30.9 nm), H = 20.9 nm the measured barrel height, and r the radius of the attached Au NS. For an upright barrel with equatorial sidewall contact, the projected edge-to-edge separation is governed by the barrel diameter:

$$g_{\text{upright}} = 2R_b = 30.9 \text{ nm} \tag{S10}$$

However, when both Au NSs rest directly on the support film, their center height above the film is $z = r$. For 100 nm-scale spheres, $r > H$ (20.9 nm), meaning the equators of the spheres lie well above the top rim of the barrel. As the two diametrically attached spheres approach each other over the barrel, their movement is arrested not by the sidewall, but by contact with the barrel's top rim. At this contact height H, the horizontal distance x from the sphere center to the contact point is $x = \sqrt{r^2 - (r - H)^2}$. The center-to-barrel-axis distance is $R_b + x$, yielding a theoretical minimum projected edge-to-edge gap at the particle equators:

$$g_{\min} = 2\left\{R_b + \sqrt{r^2 - (r - H)^2} - r\right\} \tag{S11}$$

For device A (r = 48.3) in section 27, this yields $g_{\min}$ = 13.9 nm. The measured gap for Device A (17.4 ± 0.2 nm) and the population mean (21.9 ± 3.6 nm) both fall within the idealized support-limited geometric range defined by $g_{\min}$ = 13.9 nm and g_{upright} = 30.9 nm. Thus, the substantial gap contraction in the diametric motif is consistent with—and can be quantitatively accounted for by—the 3D top-rim contact model without requiring barrel collapse as its primary explanation (**Figure S36**).

In contrast, the principal adjacent-site motifs assembled from 40 and/or 100 nm Au NSs have population mean gaps within +0.1 to +0.8 nm of their nominal design values. The deliberately pivoting-sensitive 60 nm single- and dual-barrel controls show larger deviations (+17.2 and +2.9 nm, respectively) and are therefore treated separately as constraint-dependent stress tests. Secondary grid-preparation perturbations, including evaporation-driven capillary forces, linker

rotation, particle–substrate interactions, and 2D projection, contribute in a construct-dependent manner across different assemblies. Rather than assigning a uniform mechanism (such as capillarity alone) to all constructs, capillary drying is treated as one component of these broader grid-preparation perturbations. A comprehensive summary comparing nominal design gaps and population-level projected dry-state gaps across all nine motifs is provided in **Table S9**.

Because the DNA barrel is directly resolvable in the 60 nm single-barrel stress-test dimers, we quantified the projected cross-sectional aspect ratio (major-to-minor axis ratio) across all 100 images (**Figure S37**). The loaded barrel exhibits an aspect ratio of 1.16 ± 0.11 (mean ± s.d., N = 100 from **Figure S26**), compared to 1.08 ± 0.07 for the Au-NS-free barrel origami (N = 100; Section 30). The loaded distribution shows a modest increase and broadening relative to the unloaded distribution, while the mean projected aspect ratio remains close to unity.

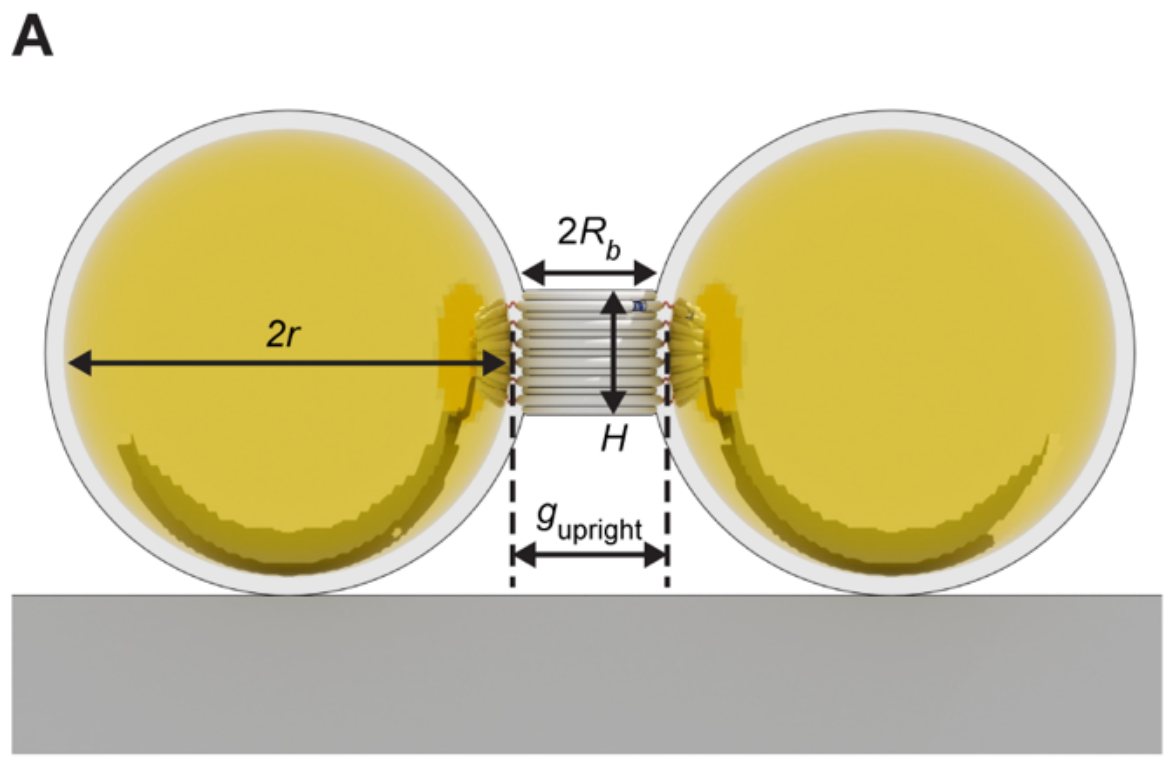

Fig. S36 | Schematic of support-limited top-rim contact geometry for symmetric dimers. (A) Fully upright orientation where the interparticle edge-to-edge separation ($g_{upright}$) is governed by the barrel outer diameter ($2R_b$), yielding $g_{upright} \sim 2R_b$. **(B)** Support-limited top-rim contact geometry on a substrate grid. When two Au NSs with radius r (where $r > H$) rest on the support film, their inward approach over the barrel (with height H) is arrested by contact with the barrel's top rim, establishing an idealized lower geometric limit g_{min} at their equators. The schematic illustrations were created using Blender, an open-source 3D creation suite.

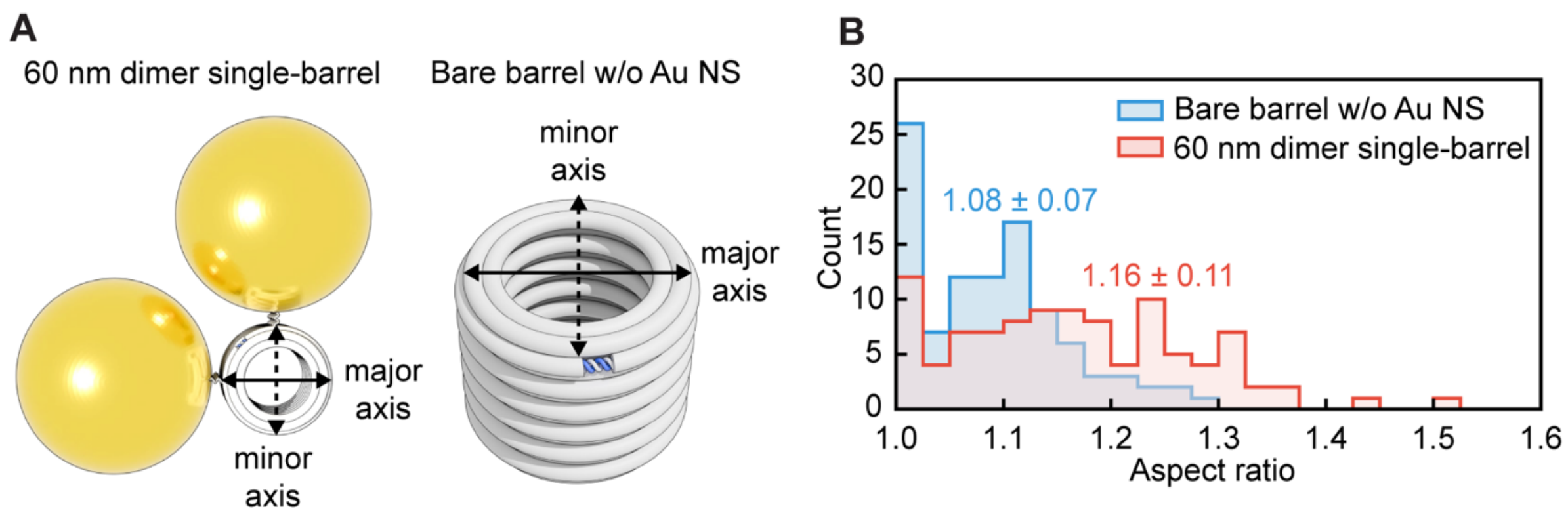

Fig. S37 | Quantitative aspect-ratio analysis of DNA origami barrels under loaded and unloaded conditions. (A) Schematics illustrating the major and minor axes for the 60 nm single-barrel dimer (left) and the Au-NS-free bare barrel origami (right). **(B)** Histograms of

projected aspect ratios measured from individual high-magnification TEM images **(Figure S26 and Section 39)**. The resulting values are 1.08 ± 0.07 for the Au-NS-free bare barrel origami and 1.16 ± 0.11 for the loaded 60 nm single-barrel dimer (mean ± s.d., N = 100 each). The schematic illustrations were created using Blender, an open-source 3D creation suite.

Table S9 | Nominal design gaps and population-level projected dry-state TEM gaps. The difference is descriptive and includes all grid-preparation, linker, substrate, and projection effects. Projected dry-state values are mean ± SD.

Motif	Nominal design gap (nm)	Projected dry-state TEM gap (nm)	Difference
2 × 100 nm dimer	~30	21.9 ± 3.6	−8.1
100/40 nm dimer	~5	5.5 ± 2.3	+0.5
2 × 100 nm + 1 × 40 nm trimer	~5	5.7 ± 3.1	+0.7
1 × 100 nm + 2 × 40 nm trimer	~5	5.1 ± 2.5	+0.1
3 × 100 nm trimer	~1	1.8 ± 0.5	+0.8
2 × 100 nm + 2 × 40 nm tetramer	~5	5.5 ± 2.8	+0.5
60 nm single-barrel dimer	~5	22.2 ± 11.8	+17.2
60 nm dual-barrel dimer	~5	7.9 ± 2.5	+2.9
5 × 60 nm 3D pentamer	~5	Population mean not quantified (N = 10 gallery; device-specific gaps = 2.4 ± 0.3 nm)	Not assigned

29. Scope and denominators of structural and correlative optical datasets

The N = 100 structural galleries were assembled from structures possessing the correct stoichiometry after gel purification and quantify geometry conditional on the intended particle stoichiometry. Because the acquisition was directed by stoichiometry rather than based on a prespecified random-field sampling plan, retrospective target/total counting would not provide an unbiased fabrication yield. Accordingly, these galleries are used to characterize conditional structural distributions rather than population-wide fabrication yield, thereby characterizing structural accuracy conditional on correct stoichiometry.

For each motif, correlative TEM and dark-field scattering spectroscopy measurements were performed on N = 10 individual devices, from which five representative examples are displayed in **Figures S12–S13, S20-S23, S29-S30, and S32**. In this workflow, isolated nanocircuits with correct stoichiometry were pre-screened under TEM before optical acquisition, ensuring that external artifacts, such as carbon film damage, incorrect stoichiometry, or neighboring particles within the diffraction-limited spot, were excluded prior to spectroscopic measurement. **Table S10** summarizes the complete paired TEM–DF datasets across all nine motifs (N = 10 per motif), reporting the number of devices exhibiting each expected plasmonic mode along with mode-deviant exceptions. All 10 paired devices per motif are included in these mode counts; no spectrum acquired for these reported N = 10 datasets was omitted from the counts. The five examples shown in the motif-specific figures are representative displays of the complete counted set. Representative correlative examples (TEM, dark-field optical microscopy, and scattering spectra) of mode-deviant devices are presented in **Figure S38**. In these exceptions, localized structural variations incurred during grid drying, such as non-ideal interparticle gaps or out-of-plane misalignments, induce spectral shifts and suppress the expected coupled modes. A mode was scored as present when the expected polarization-dependent peak, shoulder, or Fano dip assigned by the corresponding FEM/circuit calculation was resolved above the local spectral noise within the model-predicted wavelength region; the same criterion was applied to all devices.

Table S10 | Mode fidelity of correlative TEM–DF scattering spectroscopy datasets. Summary of the motif-specific structural TEM dataset sizes (N = 100 for each planar motif and N = 10 for the 3D pentamer), the number of paired single-device TEM–DF scattering spectroscopy measurements (N = 10 per motif), device counts exhibiting each expected plasmonic mode, and mode-deviant exceptions. The reported mode fidelities evaluate the optical reliability of pre-screened devices with correct stoichiometry, rather than representing the overall chemical fabrication yield. Representative single-device correlative examples (TEM, dark-field optical microscopy, and scattering spectra) of mode-deviant devices for each motif are presented in **Figure S38**. The mode-deviant-exception count is the number of unique devices lacking at least one expected mode and therefore need not equal the sum of missing-mode occurrences across the individual mode columns.

Motif	Structural TEM dataset (N)	Correlative TEM-DF measurements (N)	Device counts per expected mode	Mode-deviant exceptions (N)
2 × 100 nm dimer	100	10	ED: 10<br>EQ:10	0
100/40 nm dimer	100	10	ED: 10<br>EQ: 9	1

Motif	Structural TEM dataset (*N*)	Correlative TEM-DF measurements (*N*)	Device counts per expected mode	Mode-deviant exceptions (*N*)
2 × 100 nm + 1 × 40 nm trimer	100	10	ED:10<br>EQ: 10<br>MD: 8	2
1 × 100 nm + 2 × 40 nm trimer	100	10	ED: 10<br>EQ: 10<br>MD: 9	1
3 × 100 nm trimer	100	10	ED: 10<br>EQ: 10<br>Fano: 8	2
2 × 100 nm + 2 × 40 nm tetramer	100	10	ED: 10<br>MD: 9<br>Fano: 8	2
60 nm single-barrel dimer	100	10	ED: 10	0
60 nm dual-barrel dimer	100	10	ED: 10	0
5 × 60 nm 3D pentamer	10	10	ED: 10<br>MD: 8	2

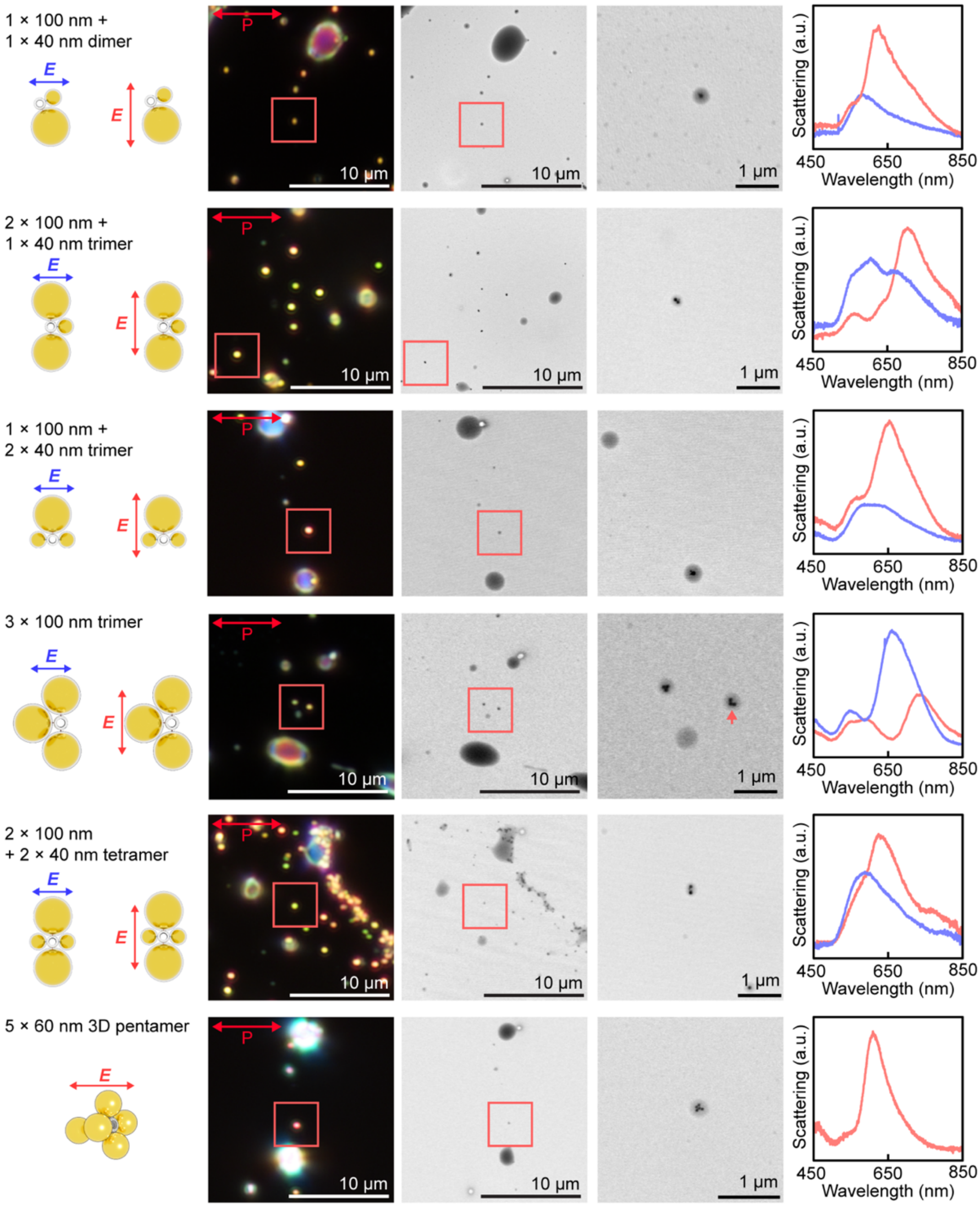

Figure S38 | Correlative TEM–DF characterization of representative mode-deviant nanocircuits. (Left to right) Schematic illustrations defining the excitation polarization axes (blue and red arrows), wide-field DF OM images with red boxes indicating the targeted single devices and red double-headed arrows (P) indicating the incident polarization direction, corresponding low-magnification TEM images, high-magnification TEM images of individual nanocircuits, and polarization-resolved single-device DF scattering spectra. In the high-magnification TEM image of the 3 × 100 nm trimer, the red arrow indicates the specific nanocircuit corresponding to the presented optical spectra. While all displayed devices possess the target stoichiometry, localized structural variations, such as non-ideal interparticle gaps, out-of-plane tilting, or rotational misalignment incurred during substrate adsorption and drying, induce spectral shifts and line broadening, ultimately leading to the suppression of the expected coupled plasmonic modes.

30. Individual-particle dimensional analysis of Au-NS-free barrel origami

To avoid measuring dimensions from the blurred edges of an averaged image, barrel dimensions were quantified directly from TEM images before averaging. The inner and outer diameters were measured from 100 individual top-view barrels, and the height was measured from 100 individual side-view barrels. The resulting mean ± SD values were 17.6 ± 1.0 nm for the inner diameter, 30.9 ± 1.7 nm for the outer diameter, and 20.9 ± 1.5 nm for the height (N = 100 per orientation). For the visualization of the mean projected top and side shapes shown in **Figure 1C** of the main manuscript, these individual images were subsequently processed using the Scipion software framework (Xmipp package) for 2D classification and averaging.

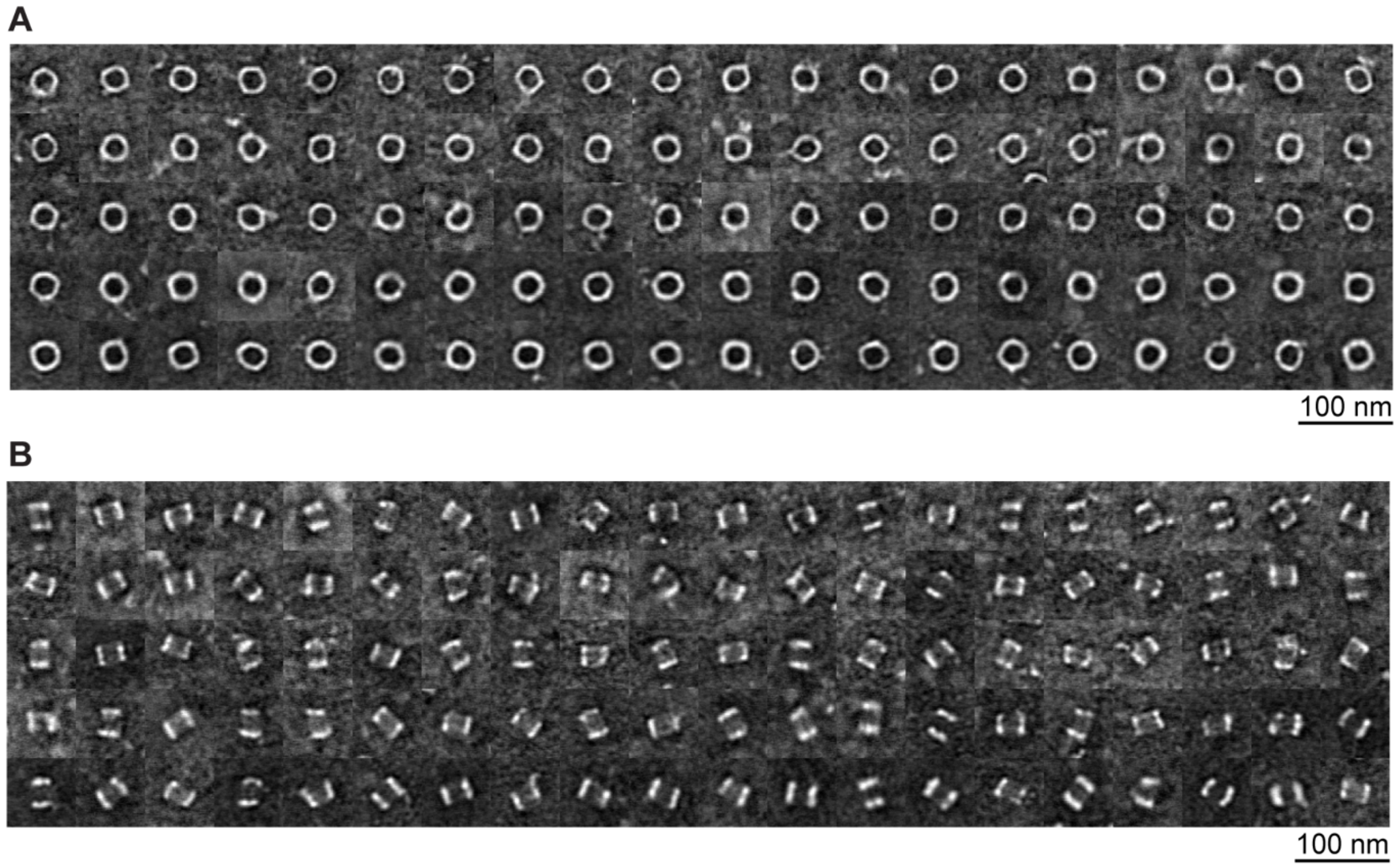

Fig. S39 | High-magnification TEM images used for individual barrel-dimension measurements. Montages of 100 individual high-magnification TEM images showing (**A**) top views and (**B**) side views of Au-NS-free barrel DNA origami. Inner and outer diameters were measured from the top-view images, and heights were measured from the side-view images before averaging. These images were subsequently used to generate the class averages shown in **Figure 1C** of the main manuscript.

Table S11 | Individual-particle dimensions of Au-NS-free barrel DNA origami.

Dimension	View used for measurement	N	Mean ± SD (nm)
Height	Side view	100	20.9 ± 1.5
Outer diameter	Top view	100	30.9 ± 1.7
Inner diameter	Top view	100	17.6 ± 1.0

31. Instrument-associated fixed-pattern background in TEM imaging

A recurrent broad background feature in some high-magnification Au NS galleries was evaluated using uncropped source acquisitions and a blank TEM control. Across independent source images, random Formvar-support contrast varies, while the broad dark shadow remains near the same detector coordinate (**Figure S40**). A similar feature is present in a blank acquisition without a sample or TEM grid (**Figure S41**), indicating an instrument-associated origin. Its detector-centered location and dependence on accumulated focused-beam exposure are consistent with localized sensitivity variation/fatigue of the scintillator–detector system. Because targets were routinely centered before high-magnification acquisition, the fixed-pattern feature recurs at a similar position in identically sized crops. Particle-edge and gap measurements were based on the high-contrast Au NS boundaries. Image processing was limited to whole-image cropping and rotation; no local intensity adjustment, local filtering, or background subtraction was applied.

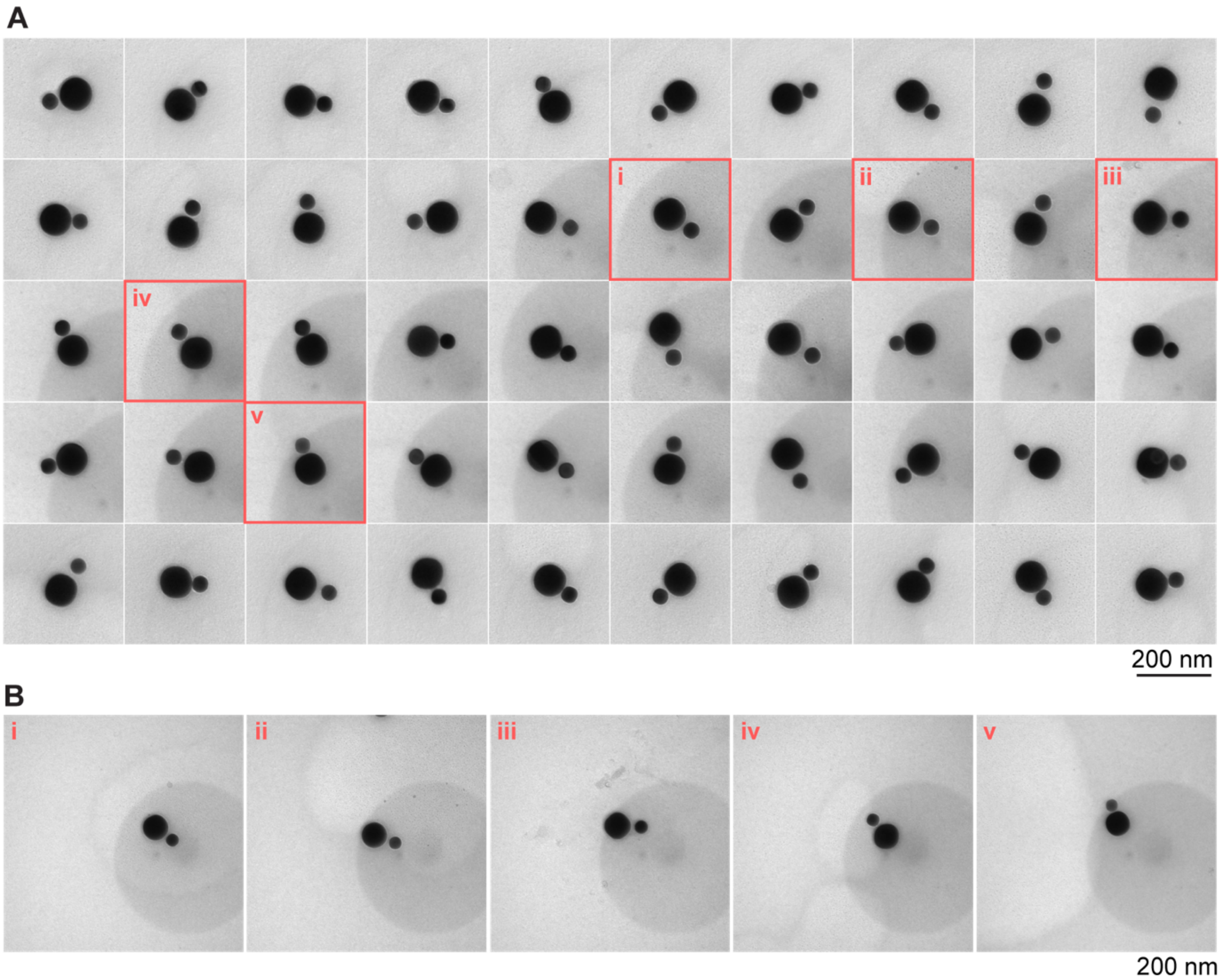

Fig. S40 | Uncropped source images demonstrating the instrument-associated fixed-pattern background. (**A**) Independent high-magnification crops of asymmetric dimers consisting of 100 nm and 40 nm Au NSs; red boxes i-v mark representative examples. (**B**) Corresponding uncropped full-field source images. Although random Formvar-film features vary among fields, the broad dark shadow remains at a similar detector-relative position, indicating a detector-fixed rather than specimen-associated origin.

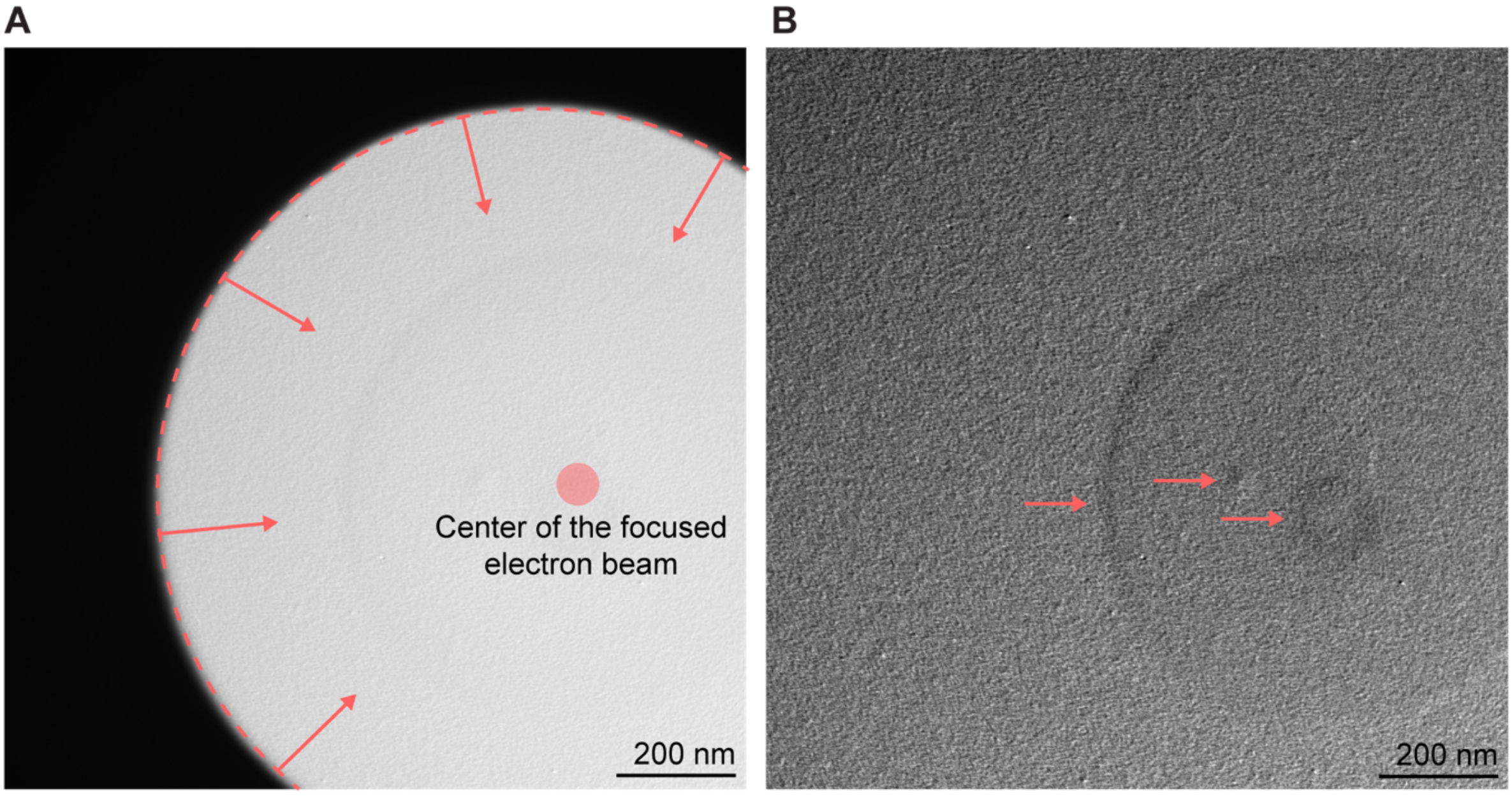

Fig. S41 | Blank TEM control for the instrument-associated fixed-pattern response. (**A**) Image of the strongly converged electron beam, indicating the detector-centered region exposed to the highest electron dose during focusing and magnification changes. (**B**) Uncropped image acquired without a sample or TEM grid. A similar broad dark feature is visible near the detector center (arrows), supporting an instrumental origin.

32. Sensitivity of nanocircuit optical resonances to gap geometry and dielectric environment

To systematically investigate the sensitivity of nanocircuit optical resonances to physical nanogap dimensions versus surrounding dielectric parameters, and to verify that the spectral correspondence does not stem from compensating parameter artifacts, numerical simulations were performed on a representative 2 × 100 nm + 2 × 40 nm tetramer motif (**Figure S42**).

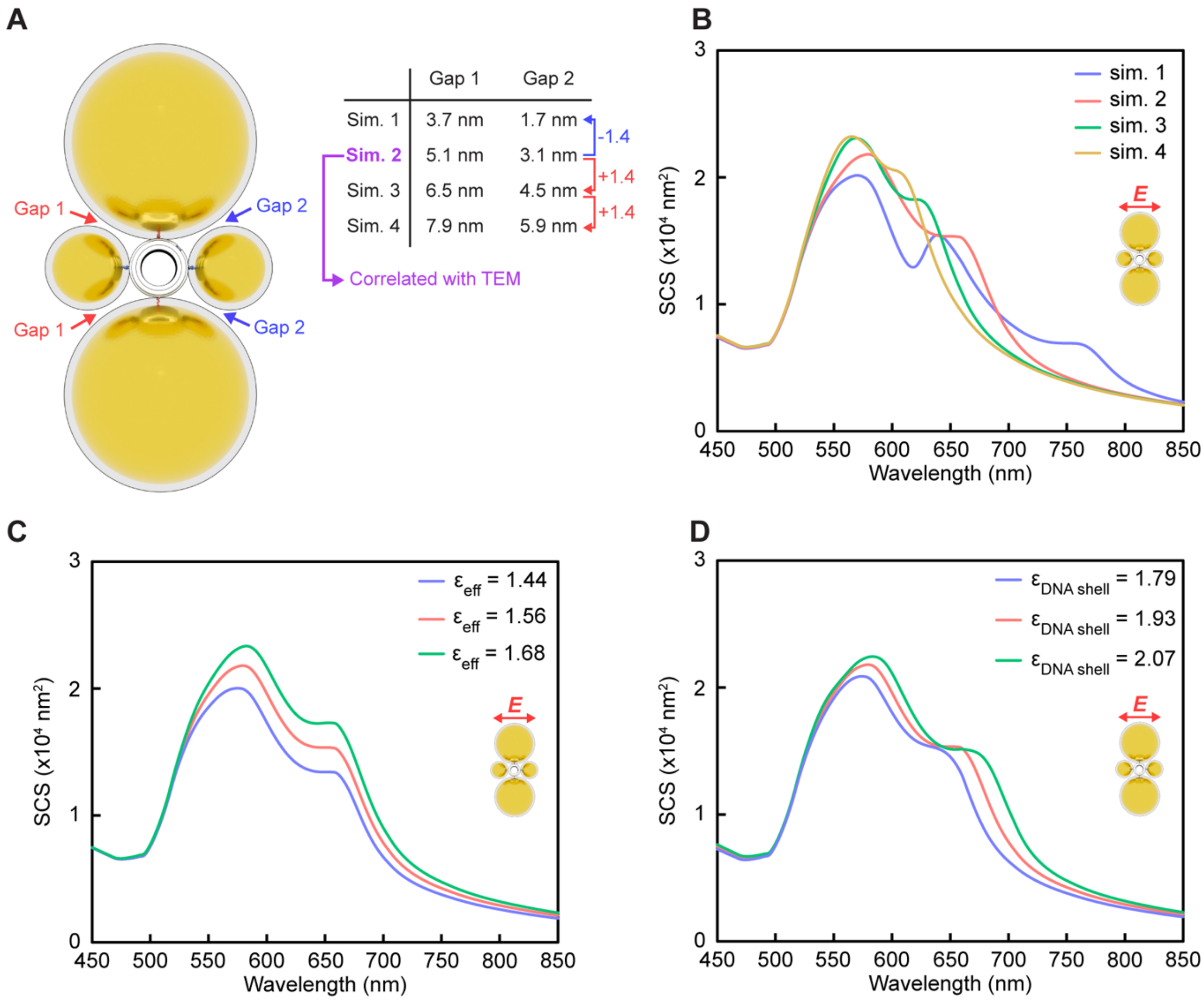

Fig. S42 | Sensitivity analysis of nanocircuit optical resonances. (A) Structural schematic and parameter table for gap distance variations of the tetrameric nanocircuit. Sim. 2 closely reflects the correlative TEM geometry. **(B)** Calculated SCS spectra under varying gap distances (Sim. 1–4). **(C, D)** Calculated SCS spectra based on the Sim. 2 gap geometry under (C) varying effective background permittivity and (D) varying ssDNA shell permittivity.

First, we evaluated the effect of varying the interparticle gap distances relative to the reference geometry (Sim. 2: Gap 1 = 5.1 nm, Gap 2 = 3.1 nm), which closely reflects the correlative TEM measurement (**Figure S42A**). Relative to the TEM-correlated reference geometry of Sim. 2, both gaps were varied in 1.4 nm increments: −1.4 nm in Sim. 1, +1.4 nm in Sim. 3, and +2.8 nm in Sim. 4. Across the full tested range, the SCS spectra undergo pronounced modifications, including resonance shifts exceeding 100 nm and substantial changes in the multi-peak coupled plasmon profile (**Figure S42B**).

Second, using the fixed gap geometry of Sim. 2, we examined the influence of potential uncertainty in the substrate dielectric environment. In our standard modeling, the effective refractive index was approximated by averaging the refractive indices of the Formvar support film (n_{Formvar} = 1.50) and air (n_{air} = 1.00), giving n_{eff} = 1.25 and $\varepsilon_{\text{eff}} = n_{\text{eff}}^2$ = 1.56. To assess a broad model range without conflating refractive-index and permittivity averaging, εeff was varied directly from 1.44 to 1.68 around the baseline value of 1.56 (**Figure S42C**); this range brackets plausible variation in the Formvar/air effective environment. Across this broad range, the calculated spectra exhibit only minor wavelength shifts (< 5 nm) and slight intensity scaling, while the overall spectral lineshape and resonance features remain well preserved.

Third, we evaluated the sensitivity to the local permittivity of the ssDNA shell within the nanogaps, also using the Sim. 2 gap geometry. While our standard calculations consistently set $\varepsilon_{\text{DNA shell}}$ = 1.93 based on the optically quantified grafting density (35), we evaluated extreme boundary conditions ranging from $\varepsilon_{\text{DNA shell}}$ = 1.79 (< 0.02 oligos/nm^2) to 2.07 (> 0.30 oligos/nm^2) based on our previous study (**Figure S42D**). Even under these extreme limits, the resulting spectral shift remains minimal (~10-20 nm) without altering the characteristic resonance mode profile.

Together, these comparisons demonstrate that the optical resonances are substantially more sensitive to nanometer-scale gap variations than to the tested dielectric-environment variations. The dielectric variations produce comparatively modest wavelength shifts while preserving the characteristic modal profile, whereas the gap perturbations substantially alter both peak positions and spectral lineshapes. Over the tested parameter ranges, the dielectric variations do not reproduce the spectral changes caused by the structural gap perturbations.

REFERENCES

1. H. Xin, B. Namgung, L. P. Lee, Nanoplasmonic optical antennas for life sciences and medicine. *Nat. Rev. Mater.* **3**, 228–243 (2018).

2. M. Kauranen, A. V. Zayats, Nonlinear plasmonics. *Nat. Photonics* **6**, 737–748 (2012).

3. J. Butet, P.-F. Brevet, O. J. F. Martin, Optical second harmonic generation in plasmonic nanostructures: From fundamental principles to advanced applications. *ACS Nano* **9**, 10545–10562 (2015).

4. C. Lee, B. Lawrie, R. Pooser, K. G. Lee, C. Rockstuhl, M. Tame, Quantum plasmonic sensors. *Chem. Rev.* **121**, 4743–4804 (2021).

5. J.-M. Nam, J.-W. Oh, H. Lee, Y. D. Suh, Plasmonic nanogap-enhanced raman scattering with nanoparticles. *Acc. Chem. Res.* **49**, 2746–2755 (2016).

6. J. Langer, D. Jimenez De Aberasturi, J. Aizpurua, R. A. Alvarez-Puebla, B. Auguié, J. J. Baumberg, G. C. Bazan, S. E. J. Bell, A. Boisen, A. G. Brolo, J. Choo, D. Cialla-May, V. Deckert, L. Fabris, K. Faulds, F. J. García De Abajo, R. Goodacre, D. Graham, A. J. Haes, C. L. Haynes, C. Huck, T. Itoh, M. Käll, J. Kneipp, N. A. Kotov, H. Kuang, E. C. Le Ru, H. K. Lee, J.-F. Li, X. Y. Ling, S. A. Maier, T. Mayerhöfer, M. Moskovits, K. Murakoshi, J.-M. Nam, S. Nie, Y. Ozaki, I. Pastoriza-Santos, J. Perez-Juste, J. Popp, A. Pucci, S. Reich, B. Ren, G. C. Schatz, T. Shegai, S. Schlücker, L.-L. Tay, K. G. Thomas, Z.-Q. Tian, R. P. Van Duyne, T. Vo-Dinh, Y. Wang, K. A. Willets, C. Xu, H. Xu, Y. Xu, Y. S. Yamamoto, B. Zhao, L. M. Liz-Marzán, Present and future of surface-enhanced raman scattering. *ACS Nano* **14**, 28–117 (2020).

7. A. Alu, N. Engheta, The quest for magnetic plasmons at optical frequencies. *Opt. Express* **17**, 5723–5730 (2009).

8. J. A. Fan, C. Wu, K. Bao, J. Bao, R. Bardhan, N. J. Halas, V. N. Manoharan, P. Nordlander, G. Shvets, F. Capasso, Self-assembled plasmonic nanoparticle clusters. *Science* **328**, 1135–1138 (2010).

9. J. Lee, J.-H. Huh, K. Kim, S. Lee, DNA origami-guided assembly of the roundest 60-100 nm gold nanospheres into plasmonic metamolecules. *Adv. Funct. Mater.* **28**, 1707309 (2018).

10. J. H. Huh, K. Kim, E. Im, J. Lee, Y. D. Cho, S. Lee, Exploiting colloidal metamaterials for achieving unnatural optical refractions. *Adv. Mater.* **32**, e2001806 (2020).

11. G. L. Liu, Y.-T. Long, Y. Choi, T. Kang, L. P. Lee, Quantized plasmon quenching dips nanospectroscopy via plasmon resonance energy transfer. *Nat. Methods* **4**, 1015–1017 (2007).

12. Y. Choi, T. Kang, L. P. Lee, Plasmon resonance energy transfer (PRET)-based molecular imaging of cytochrome C in living cells. *Nano Lett.* **9**, 85–90 (2009).

13. G. M. Akselrod, C. Argyropoulos, T. B. Hoang, C. Ciracì, C. Fang, J. Huang, D. R. Smith, M. H. Mikkelsen, Probing the mechanisms of large Purcell enhancement in plasmonic nanoantennas. *Nat. Photonics* **8**, 835–840 (2014).

14. N. Engheta, A. Salandrino, A. Alù, Circuit elements at optical frequencies: Nanoinductors, nanocapacitors, and nanoresistors. *Phys. Rev. Lett.* **95**, 095504 (2005).

15. N. Engheta, Circuits with light at nanoscales: Optical nanocircuits. *Science* **317**, 1698–1702 (2007).

16. Y. Sun, B. Edwards, A. Alù, N. Engheta, Experimental realization of optical lumped nanocircuits at infrared wavelengths. *Nat. Mater.* **11**, 208–212 (2012).

17. J. Shi, F. Monticone, S. Elias, Y. Wu, D. Ratchford, X. Li, A. Alù, Modular assembly of optical nanocircuits. *Nat. Commun.* **5**, 3896 (2014).

18. P. W. K. Rothemund, Folding DNA to create nanoscale shapes and patterns. *Nature* **440**, 297–302 (2006).

19. S. M. Douglas, H. Dietz, T. Liedl, B. Högberg, F. Graf, W. M. Shih, Self-assembly of DNA into nanoscale three-dimensional shapes. *Nature* **459**, 414–418 (2009).

20. S. Dey, C. Fan, K. V. Gothelf, J. Li, C. Lin, L. Liu, N. Liu, M. A. D. Nijenhuis, B. Saccà, F. C. Simmel, H. Yan, P. Zhan, DNA origami. *Nat. Rev. Methods Primer* **1**, 13 (2021).

21. S. F. J. Wickham, A. Auer, J. Min, N. Ponnuswamy, J. B. Woehrstein, F. Schueder, M. T. Strauss, J. Schnitzbauer, B. Nathwani, Z. Zhao, S. D. Perrault, J. Hahn, S. Lee, M. M. Bastings, S. W. Helmig, A. L. Kodal, P. Yin, R. Jungmann, W. M. Shih, Complex multicomponent patterns rendered on a 3D DNA-barrel pegboard. *Nat. Commun.* **11**, 5768 (2020).

22. J. Lee, J. Kim, G. Posnjak, A. Ershova, D. Hayakawa, W. M. Shih, W. B. Rogers, Y. Ke, T. Liedl, S. Lee, DNA origami colloidal crystals: Opportunities and challenges. *Nano Lett.* **25**, 16–27 (2025).

23. F. N. Gür, F. W. Schwarz, J. Ye, S. Diez, T. L. Schmidt, Toward self-assembled plasmonic devices: High-yield arrangement of gold nanoparticles on DNA origami templates. *ACS Nano* **10**, 5374–5382 (2016).

24. C. Hartl, K. Frank, H. Amenitsch, S. Fischer, T. Liedl, B. Nickel, Position accuracy of gold nanoparticles on DNA origami structures studied with small-angle X-ray Scattering. *Nano Lett.* **18**, 2609–2615 (2018).

25. Z. Zhao, E. L. Jacovetty, Y. Liu, H. Yan, Encapsulation of gold nanoparticles in a DNA origami cage. *Angew. Chem. Int. Ed. Engl.* **50**, 2041–2044 (2011).

26. R. Yaadav, K. Trofymchuk, M. Dass, V. Behrendt, B. Hauer, J. Schütz, C. Close, M. Scheckenbach, G. Ferrari, L. Mäurer, S. Sebina, V. Glembockyte, T. Liedl, P. Tinnefeld, Bringing attomolar detection to the point-of-care with nanopatterned DNA origami nanoantennas. *Adv. Mater.* **37**, e07407 (2025).

27. N. Siegel, M. Sanz-Paz, J. González-Colsa, G. Serrera, F. Zhu, A. M. Szalai, K. Kołątaj, M. Fujii, H. Sugimoto, P. Albella, G. P. Acuna, Distance-dependent interaction between a single emitter and a single dielectric nanoparticle using DNA origami. *Small Struct.* **6**, 2500299 (2025).

28. K. Trofymchuk, V. Glembockyte, L. Grabenhorst, F. Steiner, C. Vietz, C. Close, M. Pfeiffer, L. Richter, M. L. Schütte, F. Selbach, R. Yaadav, J. Zähringer, Q. Wei, A. Ozcan, B. Lalkens, G. P. Acuna, P. Tinnefeld, Addressable nanoantennas with cleared hotspots for single-molecule detection on a portable smartphone microscope. *Nat. Commun.* **12**, 950 (2021).

29. K. Trofymchuk, K. Kołątaj, V. Glembockyte, F. Zhu, G. P. Acuna, T. Liedl, P. Tinnefeld, Gold nanorod DNA origami antennas for 3 orders of magnitude fluorescence enhancement in NIR. *ACS Nano* **17**, 1327–1334 (2023).

30. B. Shen, M. A. Kostiainen, V. Linko, DNA origami nanophotonics and plasmonics at interfaces. *Langmuir* **34**, 14911–14920 (2018).

31. Y. Zhang, A. C. Allen, Z. J. Petrek, H. H. Cao, D. Kumar, M. C. Goodlad, V. G. Martinez, J. Singh, J. Z. Zhang, T. Ye, Formation of linear plasmonic heterotrimers using nanoparticle docking to DNA Origami cages. *J. Phys. Chem. C* **128**, 11699–11708 (2024).

32. W. Sun, E. Boulais, Y. Hakobyan, W. L. Wang, A. Guan, M. Bathe, P. Yin, Casting inorganic structures with DNA molds. *Science* **346**, 1258361 (2014).

33. J. Lee, S. Y. Lee, D.-K. Lim, D. J. Ahn, S. Lee, Antifreezing gold colloids. *J. Am. Chem. Soc.* **141**, 18682–18693 (2019).

34. T. M. Nguyen, Y. Cho, J.-H. Huh, H. Ahn, N. Kim, K. H. Rho, J. Lee, M. Kwon, S. H. Park, C. Kim, K. Kim, Y.-S. Kim, S. Lee, Ultralow-loss substrate for nanophotonic dark-field microscopy. *Nano Lett.* **23**, 1546–1554 (2023).

35. J. Lee, S. Lee, Non-invasive, reliable, and fast quantification of DNA loading on gold nanoparticles by a one-step optical measurement. *Anal. Chem.* **95**, 1856–1866 (2023).

36. S. K. Balasubramanian, L. Yang, L.-Y. L. Yung, C.-N. Ong, W.-Y. Ong, L. E. Yu, Characterization, purification, and stability of gold nanoparticles. *Biomaterials* **31**, 9023–9030 (2010).

37. C. Ciracì, F. Vidal-Codina, D. Yoo, J. Peraire, S.-H. Oh, D. R. Smith, Impact of surface roughness in nanogap plasmonic systems. *ACS Photonics* **7**, 908–913 (2020).

38. N. Tanjeem, C. Chomette, N. B. Schade, S. Ravaine, E. Duguet, M. Tréguer-Delapierre, V. N. Manoharan, Polyhedral plasmonic nanoclusters through multi-step colloidal chemistry. *Mater. Horiz.* **8**, 565–570 (2021).

39. F. Shafiei, F. Monticone, K. Q. Le, X. X. Liu, T. Hartsfield, A. Alù, X. Li, A subwavelength plasmonic metamolecule exhibiting magnetic-based optical Fano resonance. *Nat. Nanotechnol.* **8**, 95–99 (2013).

40. K. J. Park, J. H. Huh, D. W. Jung, J. S. Park, G. H. Choi, G. Lee, P. J. Yoo, H. G. Park, G. R. Yi, S. Lee, Assembly of "3D" plasmonic clusters by "2D" AFM nanomanipulation of highly uniform and smooth gold nanospheres. *Sci. Rep.* **7**, 6045 (2017).

41. W. Fang, S. Jia, J. Chao, L. Wang, X. Duan, H. Liu, Q. Li, X. Zuo, L. Wang, L. Wang, N. Liu, C. Fan, Quantizing single-molecule surface-enhanced Raman scattering with DNA origami metamolecules. *Sci. Adv.* **5**, eaau4506 (2019).

42. K. F. Wagenbauer, C. H. Wachauf, H. Dietz, Quantifying quality in DNA self-assembly. *Nat. Commun.* **5**, 3691 (2014).

43. T. Gerling, K. F. Wagenbauer, A. M. Neuner, H. Dietz, Dynamic DNA devices and assemblies formed by shape-complementary, non–base pairing 3D components. *Science* **347**, 1446–1452 (2015).

44. J. J. Funke, H. Dietz, Placing molecules with Bohr radius resolution using DNA origami. *Nat. Nanotechnol.* **11**, 47–52 (2016).

45. D. R. Smith, J. B. Pendry, M. C. K. Wiltshire, Metamaterials and Negative Refractive Index. *Science* **305**, 788–792 (2004).

46. M. F. Limonov, M. V. Rybin, A. N. Poddubny, Y. S. Kivshar, Fano resonances in photonics. *Nat. Photonics* **11**, 543–554 (2017).

47. J. A. Fan, K. Bao, C. Wu, J. Bao, R. Bardhan, N. J. Halas, V. N. Manoharan, G. Shvets, P. Nordlander, F. Capasso, Fano-like interference in self-assembled plasmonic quadrumer clusters. *Nano Lett.* **10**, 4680–4685 (2010).

48. J. B. Woehrstein, M. T. Strauss, L. L. Ong, B. Wei, D. Y. Zhang, R. Jungmann, P. Yin, Sub–100-nm metafluorophores with digitally tunable optical properties self-assembled from DNA. *Sci. Adv.* **3**, e1602128 (2017).

49. K. Gunther, M. Mertig, R. Seidel, Mechanical and structural properties of YOYO-1 complexed DNA. *Nucleic Acids Res.* **38**, 6526–6532 (2010).

50. S. G. Lopez, M. J. Ruedas-Rama, S. Casares, J. M. Alvarez-Pez, A. Orte, Bulk and single-molecule fluorescence studies of the saturation of the DNA double helix using YOYO-3 intercalator dye. *J. Phys. Chem. B* **116**, 11561–11569 (2012).

51. E. M. Roller, L. K. Khorashad, M. Fedoruk, R. Schreiber, A. O. Govorov, T. Liedl, DNA-assembled nanoparticle rings exhibit electric and magnetic resonances at visible frequencies. *Nano Lett.* **15**, 1368–1373 (2015).

52. N. Liu, T. Liedl, DNA-assembled advanced plasmonic architectures. *Chem. Rev.* **118**, 3032–3053 (2018).

53. A. Kuzyk, R. Jungmann, G. P. Acuna, N. Liu, DNA origami route for nanophotonics. *ACS Photonics* **5**, 1151–1163 (2018).

54. J. Ma, M. X. Gao, H. Zuo, Y. F. Li, P. F. Gao, C. Z. Huang, Distance-dependence study of plasmon resonance energy transfer with DNA spacers. *Anal. Chem.* **92**, 14278–14283 (2020).

55. S. H. Park, J. Lee, C. Kim, B. Kang, A. Gopinath, S. Lee, Selective binding of DNA origami on self-assembled monolayer-patterned gold substrate. *ACS Mater. Lett.* **7**, 2565–2573 (2025).

56. Y. Cho, S. H. Park, J.-H. Huh, A. Gopinath, S. Lee, DNA as grabbers and steerers of quantum emitters. *Nanophotonics* **12**, 399–412 (2023).

57. A. Gopinath, C. Thachuk, A. Mitskovets, H. A. Atwater, D. Kirkpatrick, P. W. K. Rothemund, Absolute and arbitrary orientation of single-molecule shapes. *Science* **371**, eabd6179 (2021).

58. A. Gopinath, E. Miyazono, A. Faraon, P. W. K. Rothemund, Engineering and mapping nanocavity emission via precision placement of DNA origami. *Nature* **535**, 401–405 (2016).

59. B. Y. Lee, J. Lee, D. J. Ahn, S. Lee, M. K. Oh, Optimizing protein V untranslated region sequence in M13 phage for increased production of single-stranded DNA for origami. *Nucleic Acids Res.* **49**, 6596–6603 (2021).

60. B. Liu, P. Wu, Z. Huang, L. Ma, J. Liu, Bromide as a robust backfiller on gold for precise control of DNA conformation and high stability of spherical nucleic acids. *J. Am. Chem. Soc.* **140**, 4499–4502 (2018).

61. J. Lee, J.-H. Huh, S. Lee, DNA base pair stacking crystallization of gold colloids. *Langmuir* **36**, 5118–5125 (2020).

62. P. B. Johnson, R. W. Christy, Optical constants of the noble metals. *Phys. Rev. B* **6**, 4370–4379 (1972).

63. J. Schnitzbauer, M. T. Strauss, T. Schlichthaerle, F. Schueder, R. Jungmann, Super-resolution microscopy with DNA-PAINT. *Nat. Protoc.* **12**, 1198–1228 (2017).

64. Z. Qian, S. P. Hastings, C. Li, B. Edward, C. K. McGinn, N. Engheta, Z. Fakhraai, S.-J. Park, Raspberry-like metamolecules exhibiting strong magnetic resonances. *ACS Nano* **9**, 1263–1270 (2015).

65. E. Poppleton, M. Matthies, D. Mandal, F. Romano, P. Šulc, L. Rovigatti, oxDNA: Coarse-grained simulations of nucleic acids made simple. *J. Open Source Softw.* **8**, 4693 (2023).

66. E. Poppleton, J. Bohlin, M. Matthies, S. Sharma, F. Zhang, P. Šulc, Design, optimization and analysis of large DNA and RNA nanostructures through interactive visualization, editing and molecular simulation. *Nucleic Acids Res.* **48**, e72 (2020).

67. E.-M. Roller, L. V. Besteiro, C. Pupp, L. K. Khorashad, A. O. Govorov, T. Liedl, Hotspot-mediated non-dissipative and ultrafast plasmon passage. *Nat. Phys.* **13**, 761–765 (2017).

68. E. Ringe, J. M. McMahon, K. Sohn, C. Cobley, Y. Xia, J. Huang, G. C. Schatz, L. D. Marks, R. P. Van Duyne, Unraveling the effects of size, composition, and substrate on the localized surface plasmon resonance frequencies of gold and silver nanocubes: A systematic single-particle approach. *J. Phys. Chem. C* **114**, 12511–12516 (2010).

69. G. Kewes, F. Binkowski, S. Burger, L. Zschiedrich, O. Benson, Heuristic modeling of strong coupling in plasmonic resonators. *ACS Photonics* **5**, 4089–4097 (2018).
